# Genomics of high molecular weight plasmids isolated from an on-farm biopurification system

**DOI:** 10.1038/srep28284

**Published:** 2016-06-20

**Authors:** María C. Martini, Daniel Wibberg, Mauricio Lozano, Gonzalo Torres Tejerizo, Francisco J. Albicoro, Sebastian Jaenicke, Jan Dirk van Elsas, Alejandro Petroni, M. Pilar Garcillán-Barcia, Fernando de la Cruz, Andreas Schlüter, Alfred Pühler, Mariano Pistorio, Antonio Lagares, María F. Del Papa

**Affiliations:** 1 Instituto de Biotecnología y Biología Molecular (IBBM), CONICET- Departamento de Ciencias Biológicas, Facultad de Ciencias Exactas, Universidad Nacional de La Plata, Calles 47 y 115 (1900) La Plata, Argentina; 2 Center for Biotechnology (CeBiTec), Bielefeld University, Institute for Genome Research and Systems Biology, D-33615 Bielefeld, Germany; 3 Microbial Ecology, GELIFES, University of Groningen, The Netherland; 4 Servicio Antimicrobianos, Departamento de Bacteriología, Instituto Nacional de Enfermedades Infecciosas-ANLIS Dr. Carlos G. Malbrán, Buenos Aires, Argentina; 5 Instituto de Biomedicina y Biotecnología de Cantabria (IBBTEC), Universidad de Cantabria-Consejo Superior de Investigaciones Científicas (CSIC), 39011 Santander, Spain

## Abstract

The use of biopurification systems (BPS) constitutes an efficient strategy to eliminate pesticides from polluted wastewaters from farm activities. BPS environments contain a high microbial density and diversity facilitating the exchange of information among bacteria, mediated by mobile genetic elements (MGEs), which play a key role in bacterial adaptation and evolution in such environments. Here we sequenced and characterized high-molecular-weight plasmids from a bacterial collection of an on-farm BPS. The high-throughput-sequencing of the plasmid pool yielded a total of several Mb sequence information. Assembly of the sequence data resulted in six complete replicons. Using *in silico* analyses we identified plasmid replication genes whose encoding proteins represent 13 different Pfam families, as well as proteins involved in plasmid conjugation, indicating a large diversity of plasmid replicons and suggesting the occurrence of horizontal gene transfer (HGT) events within the habitat analyzed. In addition, genes conferring resistance to 10 classes of antimicrobial compounds and those encoding enzymes potentially involved in pesticide and aromatic hydrocarbon degradation were found. Global analysis of the plasmid pool suggest that the analyzed BPS represents a key environment for further studies addressing the dissemination of MGEs carrying catabolic genes and pathway assembly regarding degradation capabilities.

Biopurification systems (BPS) were developed to mitigate the direct contamination of surface water bodies with pesticides. BPS operate as biofilter systems in which pesticides are removed from the wastewater by sorption and biodegradation in the filter matrix^1^. BPS receive high loads of pesticides at relatively high concentrations for long periods of time, thus creating a strong and long-term selective pressure for the development and growth of pesticide-tolerant or -degrading bacteria^2^. Despite the increasing application of on-farm BPS worldwide, information on the involved microbiology is still scarce. Exposure of the indigenous bacteria to mixtures of pollutants might have fostered adaptational responses *via* horizontally acquired mobile genetic elements (MGEs). Microbial activities that promote the occupancy of a particular ecological niche may be encoded on MGEs that can move across a microbial community. Accordingly, approaches targeting the mobilome^3^ provide access to this yet-unknown genetic resource.

It is well known that horizontal genetic transfer (HGT) plays an important role in bacterial adaptation and evolution. Plasmids are significant contributors to HGT to a considerably extent^4^. However, knowledge on the incidence and diversity of plasmids in bacteria from different environments is still limited to date.

To broaden our view of the entire plasmid pools present in bacteria from environmental habitats, modern approaches utilize different methods.Among them, 1) Cesium Chloride Ethidium Bromide -gradient ultracentrifugation^5^, 2) transposon aided capture (TRACA^6^), 3) bioinformatical derivation of plasmid associated contigs/genes^7^,^8^, and 4) degradation of linear DNA with exonuclease followed by multiple displacement amplification^9^,^10^,^11^ are the most employed (see revision of Jorgensen *et al*.^12^ and Smalla *et al*.^13^). Although all methods used for plasmid isolation have several limitations they have been successfully used as strategies for plasmid targeting.

We recently reported the characterization of a collection of 35 high molecular weight (HMW) plasmids harboured by bacteria obtained from a BPS contaminated with several pesticides^14^. The sampled BPS was suggested to constitute a suitable source for the isolation of genes encoding novel catabolic enzymes and resistances to toxic elements/compounds based on the high abundance and diversity of MGEs detected in this habitat^15^,^16^. In particular, the isolate collection characterized by Martini *et al*.^14^ harbors more than 50 HMW plasmids amounting to several megabases of sequence information.

In this study, we address the isolation and genetic characterization of large circular plasmids from the bacterial collection originated from a BPS. First, extrachromosomal DNA was isolated and purified, followed by high-throughput sequencing (Illumina sequencing technology, MiSeq system). The sequence data were annotated using the GenDB platform^17^ facilitating different BLAST searches against defined databases to get an overview of plasmid-specific modules and plasmid accessory genetic elements. The analysis was focused on the detection of plasmid-specific sequences, antibiotic resistance genes and pesticide degradation genes within the plasmid DNA pool. The results presented herein provide insights into the genetic diversity and structure of these circular replicons and their accessory genetic elements. Moreover, the role of such plasmids for adaptation of the community members to the specific conditions of the BPS habitat (representing a model environment of agricultural interest) is discussed.

## Results and Discussion

### Plasmid DNA sequencing and global analysis of plasmid content

A collection of plasmid-containing bacteria isolated from an on-farm BPS used for pesticide removal was previously characterized at the taxonomic and phenotypic level^14^. To gain deeper insight into the genetic content of large plasmids harbored by these bacteria, 35 plasmid-containing isolates were chosen for plasmid sequencing. The purified plasmidic DNA was pooled and sequenced using Illumina MiSeq shotgun sequencing technology. The output of the sequencing approach amounted to a total of 2,065,817 reads with an average sequence length of 250 bp. Assembly of the 516 Mb of sequence data using the Newbler assembler (version 2.6) generated 19,962 contigs larger than 500 bp accounting for 20.1 Mb of non-redundant sequence information. The average contig size was 1,513 bp with the largest contig being 76.6 Kb in length. Automatic gene prediction using Prodigal resulted in a total of 24,069 coding sequences (CDS) in contigs larger than 500 bp.

Usually, plasmid-DNA preparations are contaminated with chromosomal DNA. To get rid of contigs representing chromosomal sequences, the assembled dataset was filtered *in silico* for contigs originating from chromosomal DNA. For this purpose, the contigs were compared to host genome sequences available in publicly-accessible databases. Taxonomic assignments of plasmid-containing host bacteria have previously been determined by sequencing of their 16S rRNA gene regions^14^. Contigs that were more than 95% identical to the chromosomes of reference strains for more than 90% of their lengths were assumed to represent chromosomal contamination and therefore were discarded. As a result, 9,386,079 bp of non-redundant sequence information remained which in first instance was supposed to originate from plasmids (hereafter designated the plasmid dataset). Thus, plasmid contigs comprised a total of 11,839 CDSs with an average GC content of 56.24%. Interestingly, a large proportion of sequences (~36%) did not match (e value: 1 × 10^−10^) to any known sequences deposited in the public nucleotide and genome databases. A similar observation was reported in other plasmid metagenomes studies^5^. Therefore, plasmids may not only supply their prokaryotic hosts with known auxiliary functions of ecological and adaptative value, but also have to be considered as a resource of so far unknown genetic information.

### Diversity of genes involved in plasmid replication, mobilization and stabilization

Plasmid-related functions were analyzed by comparison of the amino acid sequences deduced for all predicted CDSs to the domains and reference proteins deposited in the protein family (Pfam) database. The analysis was carried out using the advanced metagenomics analysis platform MGX that allows processing of large datasets such as those generated on Illumina sequencing platforms. Of the 2,065,817 reads generated by Illumina sequencing, 2,055,622 yielded hits against the Pfam database of which 368,055 were related to plasmid functions, indicating that at least 18% of the sequences represented plasmid-related genes. The protein families related to plasmid functions, i.e., replication (*rep*), stabilization/partition (*sta-par*) and mobilization (*mob*)/conjugative transfer genes found in the dataset are listed in Supplementary Tables S1, S2 and S3, respectively.

#### Classification of genes encoding proteins involved in replication

Plasmid incompatibility has been the most traditional way to classify replicons (for a review see Taylor *et al*.^18^). Replication proteins identical to those of the IncP-1, IncP-7, IncP-9 and IncW plasmids (BF_Rep48, BF_Rep13, BF_Rep52, and BF_Rep24, respectively (Supplementary Table S1)), as well as rolling-circle replication initiators (proteins of the Pfam groups Rep_1 and Rep_trans in Supplementary Table S1) are present in our plasmid dataset. But most of the replication initiation proteins identified in this work cannot be assigned to the historical incompatibility groups. Since each Inc group includes plasmids with high nucleotide identity, the Pfam database was used in this work as a more inclusive approach for the replication protein classification.

Thirteen of the fourteen families related to plasmid replication deposited in the Pfam database were identified in the plasmid dataset, illustrating the wide diversity of plasmids present in the BPS isolates. The identified Pfams were assigned to 48 different *rep* genes (Table 1). In addition, six Rep sequences which did not contain a Pfam domain strictly associated with plasmid replication were identified in the assembled data using the GenDB annotation platform (included in Supplementary Table S1). These putative Rep proteins contained Pfams domains belonging to the helix-turn-helix DNA binding domain families (HTH_36 and HTH_38) as well as to other domains. In addition, they were more than 87% identical to reference replication proteins deposited in the NCBI database. Most of the identified *rep* genes could be assigned to plasmids that were previously found in different species of the Alpha-, Beta- and Gamma-proteobacteria, *Firmicutes* and *Actinobacteria* (Table 1). These results are in concordance with the taxonomic assignments of the plasmid-containing host bacteria from the BPS as determined by 16S rRNA gene sequencing^14^. Some of the reference plasmids encoding the identified Rep proteins, such as pCAR1^19^, pND6-1^20^, pSTY^21^, and pDK1^22^ contain degradative genes, and others such as pMET-1^23^, RP4^24^, pAHH01^25^, pIE321^26^, and pTB11^27^ contain antibiotic resistance genes. Identity values between the identified Rep proteins and the best BLASTp hit in the NCBI database ranged from 46% to 100% (Table 1). Out of the 54 replication associated proteins, 40 were between 80 and 100% identical to the closest protein deposited in the NCBI database, while the identity values of the remaining 14 ranged from 46 to 79% (Supplementary Table S1). Identification of replication proteins that are not very closely related to known ones extends our understanding of the diversity of plasmid replication modules and highlights such diversity.

#### Classification of genes involved in plasmid stability and partitioning

Stabilization and partitioning functions concern genes involved in plasmid maintenance and inheritance during the process of cell division. The two main mechanisms responsible for plasmid stabilization/partition, i.e. post-segregational killing and active partitioning systems, were highly represented in the dataset. A final amount of 128 genes associated with these functions were found. In total, 21 of 23 known *par-sta* related Pfam families were identified, including ParBc (PF02195) and CbiA (PF01656) as being the most abundant ones (see Supplementary Table S2).

#### Analysis of the mobilization gene set in the BPS plasmid dataset

The spread of antimicrobial-resistance, virulence and biodegradation traits are outstanding examples of the impact of HGT on the adaptation of bacterial communities under stress, with relevant consequences for the anthropogenic activities^28^. Plasmids have been pinpointed as the main DNA vehicles that transfer genes between bacterial chromosomes, with conjugation as the preponderant transfer mechanism.

Bacterial plasmids that are transmissible by conjugation can be either conjugative (i.e., self-transmissible) or mobilizable. The former plasmids encode all genes required for transfer (namely genes for a relaxase, a coupling protein (T4CP) and a mating-pair formation system (MPF), while the latter generally encode only the relaxase gene but lack VirB4 and MPF and then need a helper plasmid for transfer^29^,^30^,^31^,^32^,^33^,^34^. Analysis of the phylogenetic relationships among the relaxases allows the classification of transmissible plasmids into eight (MOB) families^29^,^30^,^32^,^33^,^34^. Self-transmissible plasmids were in turn also grouped into eight MPF families^34^,^35^.

**Figure 1:**
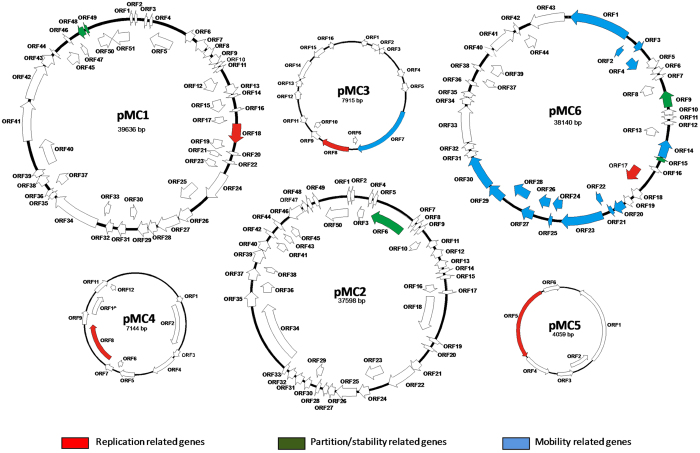
Physical maps of the six closed replicons obtained from sequence assemblies. Maps of complete nucleotides sequences of the plasmids pMC1, pMC2, pMC3, pMC4, pMC5 and pMC6 indicating locations of predicted open reading frames (ORFs)are depicted. ORFs are colour coded according to their predicted function as indicated in the associated key. Predicted products and more details regarding putative functions of annotated ORFs are provided in Table S4.

Through the classification of plasmid mobility types the diversity of plasmids and their propagation routes in complex ecosystems can be addressed^36^. In our dataset 12% of the predicted gene products matched a hit involved in bacterial conjugation. Such hits correspond to plasmid sequences whose taxonomic distribution mainly represents the phyla Proteobacteria (Alpha, Beta and Gamma classes), Firmicutes and Actinobacteria. Regarding the completely sequenced plasmids, only two of the six fulfilled the mentioned requirements that allowed the prediction that are transmissible via conjugation. Plasmid pMC3 encodes only a relaxase, and would therefore be only mobilizable, while pMC6 encodes a relaxase next to the T4CP and the MPF system, thus being conjugative (Fig. 1).

**Figure 2:**
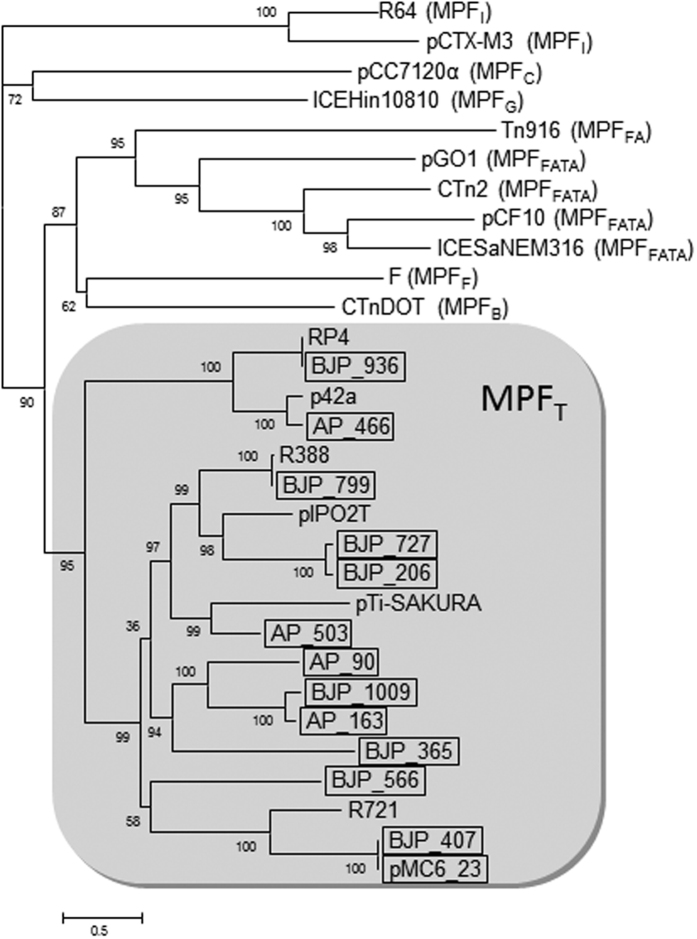
Phylogenetic analysis of the VirB4-like proteins of the plasmid dataset. The analysis includes 13 proteins belonging to the CagE-TrbE-VirB pfam ID in Table S3 and representatives of the eight mating pair formation systems defined in Guglielmini *et al*.^35^. The MPF_T_ cluster is shadowed in grey. VirB4-like proteins of the plasmid dataset are boxed.

Here we analyzed the phylogenetic relationships of the predicted VirB4-like proteins and relaxases of the plasmid dataset to study the diversity of the transmissible plasmids in a BPS environment. VirB4-like proteins are descriptors of the MPF systems and thus serve as an indication of the presence of conjugative plasmids, while relaxases are indicators of either self-transmissible or mobilizable plasmids. Thirteen VirB4 proteins and nineteen relaxases were identified in the plasmid dataset (Supplementary Table S3). On the basis of the enumerations, it appears that the proportion of conjugative versus mobilizable plasmids in our plasmid dataset was higher than that in other metagenome studies of environmental plasmid communities^37^. As can be seen in Fig. 2 all predicted VirB4 proteins identified in this work belong to the MPF_T_ type. This MPF type is characterized for including up to eleven proteins (VirB1-VirB11) which are involved in the secretion channel assembly and pilus biogenesis^38^,^39^. Plasmids encoding MPF_T_ systems are broadly distributed in bacterial hosts of all classes of the phylum Proteobacteria^34^.

A total of 19 relaxases were identified in this plasmid dataset: two MOB_F_, four MOB_Q_, one MOB_V_ and twelve MOB_P_. The MOB_F_ relaxases were placed in the MOB_F11_ subfamily (Supplementary Fig. S2). Relaxase BJP_1037 was identical to those of IncW plasmids (e.g. R388 and R7K), while BJP_380 was highly similar to relaxases of the IncP-9 plasmids, which were found to be prevalent in polluted environments^40^,^41^ and generally encode xenobiotic-degradation genes (e.g. pWW0 and NAH7). MOB_F11_ subfamily only encompasses auto-transmissible (conjugative, non- mobilizable) members^32^, which exhibit a high synteny in their conjugation genes^42^.

The MOB_Q_ relaxases that were predicted in the BPS plasmid dataset grouped in two monophyletic clusters (Supplementary Fig. S3). Protein AP_265 grouped with the conjugative relaxases of *Rhizobium/Agrobacterium* plasmids in subfamily MOB_Q2_. The MOB_Q2_ relaxases are involved in the plasmid conjugation process being different from the MOB_P2_ VirD2-like proteins that perform the T-DNA transfer from bacteria to plant cells^32^. The predicted proteins BJP_6246, BJP_4415 and BJP_1723 clustered in a clade that has not been classified^32^. It includes plasmid relaxases from diverse hosts belonging to the Alpha*-* and Gamma-proteobacteria. A gene for a MOB_V_ relaxase was found in one of the completely sequenced plasmids, pMC3. This predicted protein, pMC3_7, clustered with MOB_V4_ relaxases (Supplementary Fig. S4). Such relaxases are generally encoded on small mobilizable plasmids hosted in *Bacillus* and *Streptococcus*. MOB_V_ mobilizable plasmids are transferred by a wide variety of helper systems^43^.

MOB_P_ is the most abundant and diverse relaxase family^32^,^34^. Accordingly, most predicted relaxases found in our metamobilome belonged to this family (proteins BJP_1498, AP_669, BJP_441, BJP_847, BJP_634, BJP_2740, AP_394, AP_113, BJP_237, BJP_387, BJP_198 and pMC6_1) (Supplementary Fig. S4). The first three constitute MOB_P1_ relaxases. BJP_1498 and AP_669 were practically identical to the MOB_P11_ relaxase TraI of the IncP-1 prototype RP4. IncP-1 plasmids have previously been found to be abundantly present in pesticide-polluted environments^15^,^40^,^44^. Relaxase BJP_441 belonged to another MOB_P1_ subfamily, the MOB_P13_ cluster. Other previously classified MOB_P_ subfamilies were also represented by genes in the BPS plasmid dataset: MOB_P6_ includes proteins BJP_387, BJP_198 and pMC6_1, the relaxase of the completely sequenced plasmid pMC6, while MOB_P7_ subfamily contained protein BJP_237 (Supplementary Fig. S4). Placed in different uncharacterized MOB_P_ clades were proteins BJP_847, AP_113, BJP_2740 and AP_394 (these two last in the same clade). As can be observed in Supplementary Fig. S5a, protein BJP_634 is a singular relaxase with no close homologs. All MOB_P_ relaxases of this plasmid dataset were included in subfamilies populated by self-transmissible instead of mobilizable plasmids.

The high diversity of predicted MOB relaxase families found in this collection revealed a significantly larger diversity in the plasmid pool in the on-farm BPS than had been anticipated from the previous analyses by DNA hybridization^14^. The results of this study, based on the DNA sequencing of a BPS mobilome, also revealed that the plasmids types found in this environment differed from those present in other complex environments such as clinical settings (see for instance^45^). This observation suggests the occurrence of plasmid specialization emphasizing the importance of these mobile elements in the dynamics of local bacterial communities.

### Analysis of six completely assembled plasmid replicons

Assembly of plasmid replicons was particularly challenging due to the short read length of the Illumina sequence reads and the high abundance of insertion sequence elements, transposons, and other repetitive elements in plasmid genomes. Read assembly generated six complete circular replicons which ranged in size from 4 to approximately 40 kb. The main characteristics of the plasmids and their genomic maps are shown in Table 2 and Fig. 1, respectively. The closest matches of the plasmid-encoded gene products as identified by BLASTp analysis are listed in Supplementary Table S4. Sequence analysis of plasmid replicons showed a large number of CDSs coding for hypothetical proteins when compared to the NCBI database, suggesting that these replicons carry many uncharacterized functions. *In silico* analysis of the replicon-encoded accessory modules uncovered (i) replicon-like prophages (pMC1, pMC2), and (ii) toxin-antitoxin–like components (pMC1, pMC3, pMC6).

Accordingly, four of the six assembled replicons could be classified as plasmids (pMC3, pMC4, pMC5 and pMC6) whereas two of them were considered to represent phages (pMC1 and pMC2). Concerning the latter, the majority of their CDSs were predicted to encode phage proteins, although pMC1 contains a hypothetical protein with low identity to a plasmid replication protein. The three smaller plasmids (pMC3, pMC4 and pMC5) contained one *rep* gene each and most of their CDSs code for hypothetical proteins. Plasmid pMC6 harbors *tra* and *mob* genes including a relaxase gene and MPF class T genes (according to^35^), suggesting that it is conjugative. This plasmid has a chimeric composition, mostly associated with S*almonella enterica* sequences, with a high percentage of identity, next to a smaller fragment that is more similar to *Pseudomonas* species sequences (see Supplementary Table S4).

Plasmid pMC1 harboured genes predicted to encode proteins with high similarity to HicA/HicB. In particular, the HicA/B toxin-antitoxin pair belongs to the type II of TA systems. Most predicted gene products of pMC3 showed homology to hypothetical proteins from *Bacillus cereus*. This replicon contains a CDS with high similarity to barstar antitoxin gene. Barstar plays a role as inhibitor of barnase, representing a ribonuclease that cleaves RNA yielding 3′-nucleotides through a 2,3-cyclic intermediate^46^. In plasmid pMC3 is not encoded a barnase homologous protein. However, it is conceivable that presence of a barstar gene may protect a host bacterium from deleterious effects of a corresponding barnase toxin. Replicons pMC1 and pMC2 carry eight and nine phage related genes, respectively. Although pMC1 also contains a CDS that potentially encodes an integrase, no putative replication initiation genes were detected.

Putative replication initiation genes were only identified in pMC3, pMC4, pMC5 and pMC6. The Rep protein from pMC4 and three other proteins encoded in this replicon are related to corresponding *Acidovorax* proteins. In particular the average GC-content of *Acidovorax* sp. approximately is 64% and that of the pMC4 replicon is 57.5%.

The *rep* gene from pMC6 is most closely related to that of *Salmonella enterica* subsp. enterica serovar Agona str. SL483 plasmid (GenBank accession number CP001137.1) (99% identity at amino acid level). In addition, further 29 from 44 CDSs are related to *Salmonella* sp. genes. The average GC-content of this genus is approximately 52%, which is higher than GC-content of pMC6 (approx. 45.7%) (see Supplementary Table S4). In this regard, based on genomic data Nishida^47^ and Shintani *et al*.^31^ reveled that the GC contents of the majority of plasmids is lower than the GC contents of its host chromosome. Plasmid pMC6 also carries genes for three cysteinyl-tRNA synthetase. Although vague, presence of these genes may be beneficial for growth of the host bacterium under cysteine-limiting conditions.

Genes of direct adaptive value such as antibiotic and heavy metal resistance and catabolic genes were not evident in these six closed circular replicons.

### Functional predictions based on assembled contigs from the sequence dataset

In order to investigate the genomic content of the plasmids, antibiotic and metal resistance genes as well as genes involved in the degradation of pesticides and other toxic compounds were studied. To address the question whether identified accessory genes are plasmid-related, context information represented on assembled contigs was used.

#### Presence of antibiotic resistance genes

**Figure 3:**
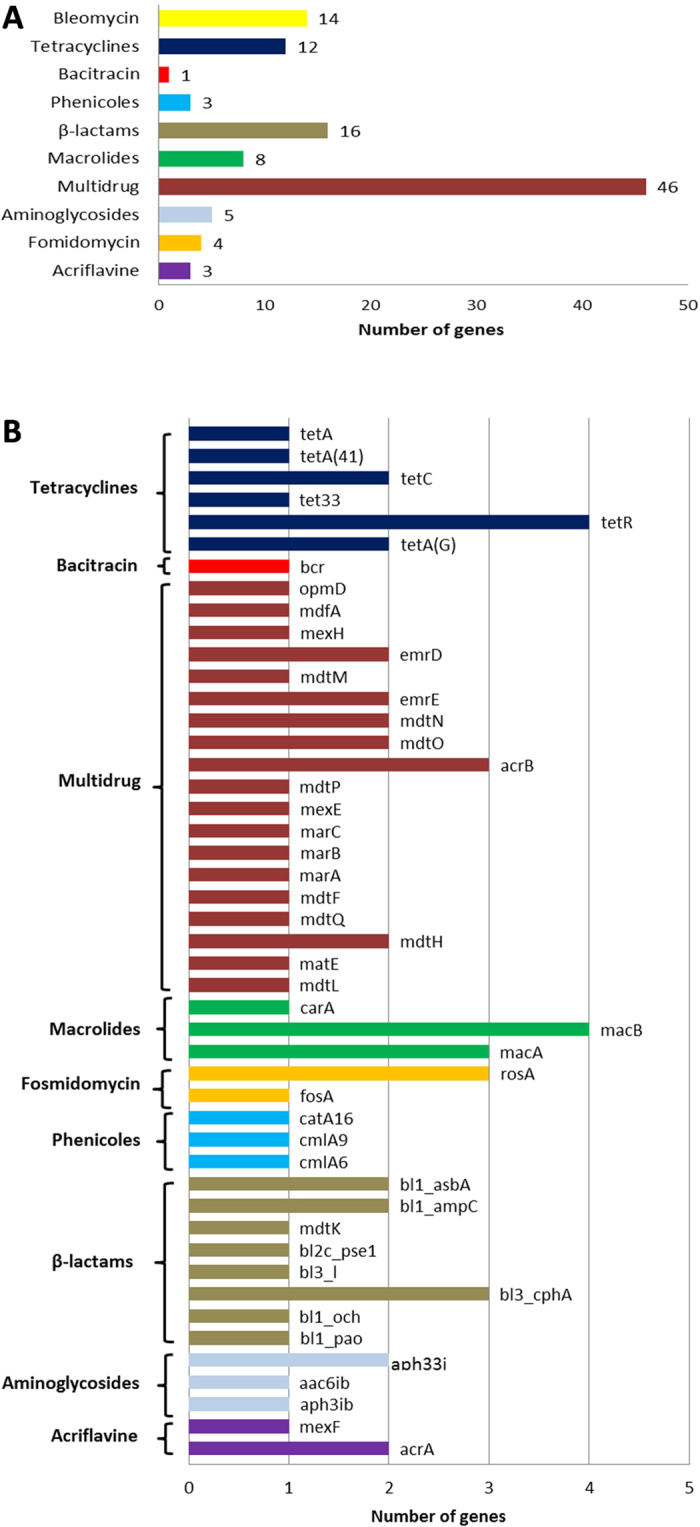
(**A**) Classes of antibiotic resistance genes presents in the plasmid dataset. (**B**) Antibiotic resistance genes present in the BPS plasmid dataset.

Antibiotic resistance genes (ARGs) are ubiquitous in bacterial communities in various ecological niches. In a previous report^14^ we showed that the isolates from the BPS collection were able to grow in the presence of different antibiotics and feature a great variability in their resistance levels. It was further demonstrated that different resistance genes are encoded on plasmids. Automatic annotation within the annotation tool GenDB revealed the presence of 112 ARGs predicted to confer resistance to tetracyclines, macrolides, β-lactams, aminoglycosides, bleomycin, fosmidomycin, bacitracin, phenicols and also to acriflavine, (Fig. 3A and Supplementary Table S5), indicating that a large number and different types of resistance genes are present in the plasmid dataset. In order to identify and classify the ARGs present in the sequenced plasmids, a BLAST search was performed against the Antibiotic Resistance Database (ARDB) (http://ardb.cbcb.umd.edu/)^48^. In total, 72 CDS were assigned to an antibiotic gene (Fig. 3).

The prevalent ARGs belong to the multidrug resistance/efflux pump systems (41% of all ARGs). Multidrug efflux systems contribute significantly to the increased resistance to multiple antibiotics^49^ as well as to other compounds used as antimicrobial agents, such as quaternary ammonium compouds^50^ in bacteria. In the assembled sequence data representing the BPS plasmids, three contigs encode multidrug efflux systems. The operons *mar*ABCR, *mdt*IJ and *mdt*ON were identified (shown in Supplementary Table S5).

Concerning to β-lactamases, 16 corresponding genes were identified and classified in eight gene variants (see Fig. 3B). Identified β-lactamases show 40 to 100% identity to reference enzymes. These results suggest the presence of putative novel lactamases. Lactamases are of particular interest since β-lactams are the most widely used class of antibiotics, and MGEs, mainly plasmids, are the vectors mediating their dissemination^51^.

Aminoglycosides are among the most commonly used broad-spectrum antibiotics. Resistance is mainly conferred by enzymes that modify aminoglycosides through phosphorylation, adenylation or acetylation^52^. Two putative genes for phosphotransferases were found, one of which corresponds to *aph*A that is present in the plasmid pRK2 (GenBank accession number CP002187). Next, the adjacent CDSs in the contig were analyzed and the presence of *tra*ABC genes from pRK2 was confirmed.

Three putative chloramphenicol resistance genes were detected. Two of them (*cml*A9 and *cml*A6) are identical to corresponding genes located on the variable region of class 1 integrons identified in different species. On one contig, the typical structure *cmlA*9–*tet*R–*tet*A–*lys*R was found. Neither the *int*I1 gene nor any other elements associated with class 1 integrons were detected on this contig.

Macrolide and glycopeptide resistance genes are homologous to previously described ones in environmental samples. The gene *mac*B, which encodes a macrolide-specific efflux system, was detected. These genes can easily be transferred from one host to another since they are usually located on mobile elements such as plasmids^53^ and transposons^54^. Finally, tetracycline resistance genes were detected and all of them show a high percentage of identity with the already described ones (*tet*A, *tet*C, *tet*33, and *tet*R) in plant-associated bacteria, human and animal pathogens.

Overall, *in silico* analysis showed that BPS bacteria are a reservoir of novel and unknown genes that have not yet been isolated from other specimens and environments. The advantage that resistance genes confer to microorganisms exposed to anthropogenic antibiotic pressure is undisputed. However, the role of resistance genes in bacteria in environments that are not directly affected by the application of antibiotics is less clear^55^, and not limited to antimicrobial defense. Bacteria can have proteins that provide antibiotic resistance beyond their primary function^56^.

#### Presence of heavy metal resistance genes

Transposons carrying mercury resistance determinants (*mer*) are widely distributed in clinical and environmental bacteria^57^. A contig that carries a truncated narrow spectrum *mer* resistance transposon inserted upstream of a *tnp*A gene was detected in the plasmid dataset. The *mer* gene cluster encodes a regulatory protein (MerR), a mercuric ion transport protein (MerT), a periplasmic mercuric ion binding protein (MerP), mercuric reductase (MerA), a secondary regulatory protein (MerD), and proteins of unknown functions (MerE and ORF-2). This cluster is similar to the corresponding operons present on Tn*4378* of the *Cupriavidus metallidurans* CH34 megaplasmid pMOL28, on Tn*501* of the *Pseudomonas aeruginosa*, the *Pseudomonas stutzeri* mercury resistance plasmid pPB, and the Tn*501* remnant in pJP4.

In addition, genes related to transport/resistance to arsenic, cobalt, zinc, copper, cadmium, iron, nickel, tellurite, selenium and manganese were detected in the plasmid contigs (see Supplementary Table S6).

#### Detection of genes involved in xenobiotic and other organic toxic compounds degradation

Since our sample was derived from a BPS exposed to pesticides, the microorganisms that inhabit this niche were under a strong selective pressure, may have developeded degradation abilities that allow them to survive in this environment. Since plasmids and other MGEs play a key role in bacterial adaptation to environmental changes, could be possible to associate the genes responsible for the degradation of xenobiotic compounds to MGEs. Recently, Dunon *et al*.^15^ reported that the increased prevalence of MGEs in a BPS microcosm was accompanied by an increase in the capacity to mineralize the applied pesticides. In general, functions involved in pesticide and other xenobiotic compound resistances are difficult to attribute to particular genes. The biodegradation of specific compounds often is complex requiring a series of biochemical reactions that involve different enzymes whose encoding genes remain unknown. However, preliminary identification of some genes involved in xenobiotic degradation was possible. Genes coding for the main enzymes implicated in the degradation of linuron, 2,4-dichlorophenoxyacetic acid (2,4-D), 3-fluorobenzoate, 4-fluorobenzoate, hexachlorocyclohexane (HCH), dichlorodiphenyltrichloroethane (DDT), atrazine (ATZ), 1,4-dichlorobenzene, dibenzofuran, parathion, endosulfan and methyl viologens were investigated. BLASTp analyses were done using the reference sequences listed in Table S7 against the assembled plasmid database within the GenDB platform. Two putative genes encoding the two main enzymes for 2,4-D degradation (*tfd*A and *tfd*B), the gene for the enzyme responsible for the first step of atrazine degradation (*atz*A), two genes encoding the alpha- and beta-subunits of benzoate 1,2-dioxygenase for 3-fluorobenzoate degradation, and two genes whose products participate in dibenzofuran degradation were identified (see Table S8). The major facilitator system (MFS) transporter for methyl viologen resistance as well as the enzyme required for parathion degradation were also predicted to be encoded in the plasmid dataset.

In addition, a search for aromatic hydrocarbon degrading enzymes was carried out using GenDB annotations. Different putative genes that could participate in xylene, toluene, and/or benzene utilization were detected (Table S8). Two contigs show high similarity to regions of the TOL plasmid pWW0 of *Pseudomonas putida*. This plasmid encodes the metabolic pathways for degradation of toluene, xylene and their alcohol and carboxylate derivatives^58^. A 9 kb contig contained four genes involved in the TOL upper degradation pathway (*xyl*C-*xyl*M-*xyl*A-*xyl*B) and another 2 kb contig contained three genes involved in the TOL lower degradation pathway (*xyl*I-*xyl*H-*xyl*S).

#### Presence of other MGEs in the plasmid metagenome

Plasmids often carry other MGEs such as IS elements and/or transposons. IS elements are simple MGE consisting of the gene required for transposition of the element and inverted repeats at the ends of the element. Transposons are more complex, in that they also harbor accessory genes not involved in translocation of the element. BLAST analyses of contigs against the transposon and IS database IS finder^59^ revealed a total of 120 hits. Among the transposable elements present in the plasmid contigs, those belonging to the Tn*3* family were the most abundant ones, followed by IS*3* and IS*110* family members (Table S9 and Supplementary Fig. S1).

## Conclusions

Plasmid and HGT studies on bacteria colonizing natural or semi-natural environments are important to foster our understanding of the mechanisms that facilitate bacterial adaptation to specific environmental conditions^60^,^61^. Due to recent developments in high-throughput sequencing technologies, different plasmid pool sequencing approaches have been conducted to investigate and characterize diverse soil microbial communities^12^. In the present study, we isolated and characterized the pool of large plasmids from a bacterial collection previously obtained from an on-farm BPS^14^. This biofilter imposes a strong and long-lasting pesticide selective pressure on the associated microbiome^2^. Moreover, previous reports have shown a high abundance of IncP-like plasmids and other MGEs^15^,^16^ making this environment a highly relevant target site to address bacterial adaptation processes. A limiting factor of large plasmids is the plasmid DNA purification step. Here, we used a successful method comprising cell lysis followed by CsCl density gradient centrifugation. Application of this method enabled the isolation of large amounts of HMW plasmid DNA.

Plasmid sequencing and subsequent *in silico* analysis allowed us to determine replicon types and the genetic potential regarding plasmid mobility. Moreover, information on accessory plasmid functions being of adaptive value for the host bacteria was obtained. In order to explore the host range of plasmids as well as their ability to be mobilized and maintained in different bacteria, plasmid functions were studied in detail. As mentioned above, most of the identified *rep* genes in the dataset (grouped into 13 Pfam families) corresponded to reference genes present on plasmids whose hosts represent different species of the Alpha-, Beta- and Gamma-proteobacteria, *Firmicutes* and *Actinobacteria*. This indicates that a large diversity of plasmid replicons is present in the BPS microbiome. Additionally, the detection of transfer and mobility modules suggested that this bacterial community actively exchanged information *via* plasmid transfer. Experimental evidence for transfer of plasmids of the BPS community was obtained recently^14^. Moreover, other MGEs, i.e. IS-elements and transposons, were identified in the plasmid contigs, suggesting that the flow of information among community members of the BPS can occur by HGT mechanisms.

We analyzed the presence of catabolic genes involved in degradation pathways regarding xenobiotic and other toxic compounds, since such genes are often located on MGEs^62^,^63^. Different genes whose products could participate in the degradation of 2,4-D, atrazine, 3-fluorobenzoate, dibenzofuran, parathion, methyl viologen and xylene/toluene/bencene were identified. Although many enzymes that potentially catalyze the degradation of pesticides were detected, full understanding of the underlying biodegradation pathways requires additional investigations. The present study revealed the genetic potential of biodegradation pathways for persistent pesticides/xenobiotics. However, the activity of the genes at the transcript and/or proteome levels remains to be determined. Therefore, further research involving more promising integrated metatranscriptome analyses should be conducted in order to shed light on the ecological and metabolic significance of the identified genes. Discovery of new genes of biotechnological interest is expected from such metatranscriptome studies. Future work will address functional analyses also comprising genes that currently were annotated as hypothetical ones.

Finally, our study shows the technical possibilities to conduct specific HMW plasmid metagenome analyses and highlights the importance of plasmids for local adaptation of complex microbial communities. The plasmid metagenome of the BPS community now provides the basis for continuing efforts to further understand the role of large plasmids under local selective conditions.

## Materials and Methods

### Sampling and plasmid DNA extraction

Plasmids were obtained from 35 plasmid-containing bacterial isolates previously characterized by Martini *et al*.^14^. The list of all pesticides added to the BPS (Kortrijk, Belgium) and their quantification have been previously reported^16^. Plasmids were obtained from independent 250 ml bacterial-Luria-Bertani (LB) cultures^64^ incubated at 28 °C to late exponential phase (approximately 10^8^ cells/ml). Plasmids were extracted using a modified alkaline lysis method as previously described by Jouanin *et al*.^65^. Plasmid DNA purification was performed individually to avoid some technical issues, such as differential DNA extraction efficiency from different microorganisms. At least 50 HMW plasmids were purified from other forms of nucleic acids using isopycnic density centrifugation in a classical CsCl-ethidium bromide gradient or QIAGEN Large Construction Kit following the manufacturer’s instructions. The concentration of DNA sample was measured with Quant-iT™ PicoGreen^®^ dsDNA (Invitrogen). Then the samples were pooled considering the number and size of the plasmids in each isolate in order to add approximately equal amounts of each plasmid. Finally, plasmidic DNA preparations were pooled together for sequencing.

### High-throughput sequencing and computational analysis

Sequencing was performed at CeBiTec (Bielefeld, Germany) using the Illumina Miseq technology. The reads obtained from the sequencing were assembled using Newbler 2.6. Contigs were imported into the genome annotation system GenDB version 2.0^17^ for gene analysis. Individual (short) sequences were analyzed and functionally annotated. An automated function prediction was computed using a combination of standard bioinformatic tools such as Blast and InterPro^66^. After the automatic annotation, the sequence information was refined manually. This approach leads to consistent gene annotation, assigning gene names, EC numbers, COG and GO numbers. Protein identity values were determined using BLASTp.

Plasmid finishing was accomplished using the CONSED^67^ software package as described recently^68^. Briefly, reads protruding contig ends were used to identify contigs that flank a certain source contig. These manually approved reads were used for an in silico based gap closure approach. Based on this approach, the completeness of the plasmids was established. Complete replicons were first automatically annotated using the GenDB system^17^. Then circular contigs were manually curated and genes were annotated using the best BLASTp hit at National Center for Biotechnology Information (NCBI) database (http://www.ncbi.nlm.nih.gov/). The sequences of these replicons were submitted to GenBank under the following accession numbers: pMC1 (LT158601), pMC2 (LT158602), pMC3 (LT158603), pMC4 (LT158604), pMC5 (LT158605) and pMC6 (LT158606).

For Pfam analysis, unassembled data was uploaded to the MGX platform (Jaenicke, unpublished). Pfam annotations were created using a BlastX search against seed sequences of the Pfam using an e-value cutoff of 1 × 10^−5^ with disabled sequence complexity filter; the Pfam family of the best blast hit was used to annotate the query sequence.

Plasmid dataset relaxases BJP_1037, BJP_380 AP_265, BJP_6246, BJP_4415, BJP_1723, pMC3_7, BJP_1498, AP_669, BJP_441, BJP_847, BJP_634, BJP_2740, AP_394, AP_113, BJP_237, BJP_387, BJP_198, and pMC6_1 were used as queries in BLAST searches^69^. For each MOB family, queries and hits were trimmed to keep only the N-terminal relaxase domain (300 amino acids for MOB_F_, MOB_Q_ and MOB_V_ families and 500 amino acid for MOB_P_) and aligned with MUSCLE^70^. The phylogenetic reconstruction was carried out by maximum likelihood (ML), using RAxML version 7.2.7^71^. In total 20 ML trees were executed using the JTTGAMMA model. Bootstrap trees were then inferred using the autoFC stop criterion to obtain the confidence values for each node of the best ML tree. MOB families and subfamilies were defined as in Garcillan-Barcia *et al*.^32^.

The analysis of VirB4-like proteins of the plasmid dataset included 13 proteins belonging to the CagE-TrbE-VirB pfam ID (recorded in Supplementary Table S3) and representatives of the eight mating pair formation systems defined in http://conjdb.web.pasteur.fr/conjdb/_design/conjdb/index.html^35^ (GenBank Acc. No. BAA78009.1: TraU_R64 and AAN87691.1: TraU_pCTX-M3 for MPF_I_; BAB78290.1: Alr7206_pCC7120alpha for MPF_C_; WP_014326569.1: Tfc16_ICEHin10810 for MPF_G_; AAB60017.1: Orf16_Tn916 for MPF_FA_; YP_002790923.1: TrsE_pGO1, YP_195783.1: PrgJ_pCF10, YP_001086885.1: CD418_CTn2 and CAD47019.1: Gbs1360_ICESaNEM316 for MPF_FATA_; AAC44180.1: TraC_F for MPF_F_; NP_809006.1: TraG_CTnDOT for MPF_B_; AAO43554.1: TrbE_p42a, YP_009077466.1: TrwK_R388, CAC82751.1: TraE_pIPO2T, NP_065362.1: TraE_R721; CAJ85691.1: TrbE_RP4; NP_053384.1: VirB4_pTi for MPF_T_). All proteins were aligned with MUSCLE^70^ and the maximum-likelihood phylogenetic reconstruction was performed with FastTree 2^72^.

For analysis of antibiotic resistance genes, those genes that were automatically annotated as resistance genes at the GenDB platform were confirmed and annotated according to the best BLASTp hit at NCBI and then blasted against the ARDB database^48^ for their classification at gene level. All insertion sequences (IS) were downloaded from ISfinder^59^ in order to generate a database. For such analysis, assembled contigs were used as query for BLAST against such database, using an e-value cutoff of 1e-20. The IS of the best BLAST hit was used to annotate the query sequence. For the analysis of genes involved in pesticide degradation, a BLASTp search was carried out using reference sequences for such genes (listed in Supplementary Table S1) against contigs and the GenDB platform BLASTp tool. Identification of genes involved in metal resistance and degradation of xenobiotics^73^ and aromatic hydrocarbons was done with the GenDB platform using automatic annotation and confirmed using the best BLASTp hit at NCBI.

## Additional Information

**How to cite this article**: Martini, M. C. *et al*. Genomics of high molecular weight plasmids isolated from an on-farm biopurification system. *Sci. Rep.* **6**, 28284; doi: 10.1038/srep28284 (2016).

## Supplementary Material

Supplementary Information

## Figures and Tables

**Table 1:** List of genes and classification of encoded proteins according to replication- related Pfam numbers.

**Pfam number**	**Pfam ID**	**Description**	**Different proteins**	***Organism***	**% Identity to the closest protein**
pfam00436	SSB	Single-strand binding protein family	10	*Legionella pneumophila*	50%
				*Legionella shakespearei*	49%
				*Ottowia thiooxydans*	96%
				*Pseudomonas putida*	99%
				*Ochrobactrum intermedium*	93%
				*Microbacterium yannicii*	52%
				*Pseudomonas syringae*	86%
				*Acinetobacter baumannii*	99%
				*Pseudomonas putida*	59%
				*Salmonella enterica*	100%
pfam01051	Rep_3	Initiator Replication protein	6	*Providencia alcalifaciens*	64%
				*Salmonella enterica*	99%
				*Pseudomonas putida*	100%
				*Pseudomonas taiwanensis*	71%
				*Salmonella enterica*	99%
				*Pseudomonas taiwanensis*	99%
pfam02486	Rep_trans	Replication initiation factor	5	*Acidovorax citrulli*	56%
				*Streptococcus parasanguinis*	97%
				*Acidovorax delafieldii*	48%
				*Streptococcus parasanguinis*	97%
				*Acidovorax citrulli*	56%
pfam11740	KfrA_N	Plasmid replication region DNA-binding N-term	5	*Pseudomonas aeruginosa*	100%
				*Pseudomonas putida*	46%
				*Serratia marcescens*	89%
				*Salmonella enterica*	100%
				*Pseudomonas* sp.	100%
pfam11800	RP-C_C	Replication protein C C-terminal region	5	*Ochrobactrum anthropi*	83%
				*Ochrobactrum anthropi*	89%
				*Ochrobactrum rhizosphaerae*	99%
				*Sinorhizobium meliloti*	94%
				*Ochrobactrum anthropi*	88%
pfam03428	RP-C	Replication protein C N-terminal domain	4	*Ochrobactrum rhizosphaerae*	88%
				*Sinorhizobium meliloti*	94%
				*Ochrobactrum rhizosphaerae*	95%
				*Ochrobactrum rhizosphaerae*	94%
pfam04796	RepA_C	Plasmid encoded RepA protein	3	uncultured bacterium	62%
				uncultured bacterium	53%
				*Enterobacteriaceae*	100%
pfam07042	trfA	TrfA protein	2	*Thauera terpenica*	68%
				*Pseudomonadales*	100%
pfam05472	Ter	DNA replication terminus site-binding protein (Ter protein)	2	*Escherichia coli*	99%
				*Pseudomonas* sp.	100%
pfam01446	Rep_1	Replication protein	2	*Bacillus pumilus*	94%
				*Bacillus pumilus*	98%
pfam03090	Replicase	Replicase family	2	*Pseudomonas* sp.	90%
				*Aeromonas hydrophila*	99%
pfam06504	RepC	Replication protein C (RepC)	1	*Pseudomonas aeruginosa*	96%
pfam02387	IncFII_repA	IncFII RepA protein family	1	*Klebsiella pneumoniae*	96%

**Table 2:** Main characteristics of the six closed replicons.

**Replicon Name**	**Replicon type**	***rep*** **gene**	***rep*** **gene ID (see** Table S5)	***rep*** **Pfam**	**Lenght**	**Number of CDSs**	**% GC content**
pMC1	phage*	yes	CDS_18	No detected	39.636 bp	51	51,94
pMC2	phage*	no	–	–	37.598 bp	50	60,9
pMC3	plasmid	yes	CDS_8	No detected	7.915 bp	16	31,78
pMC4	plasmid	yes	CDS_8	pfam02486	7.144 bp	12	57,5
pMC5	plasmid	yes	CDS_6	pfam02486	4.059 bp	6	30,92
pMC6	plasmid	yes	CDS_17	pfam01051	38.140 bp	44	45,69

^*^These replicons were considered to represent phages, since the majority of their CDSs encode phage proteins.
