# Supplementary material for: Genomics of high molecular weight plasmids isolated from an on-farm biopurification system
Source: Sci Rep. 2016 Jun 20;6:28284. doi: 10.1038/srep28284 (PMC4913263; doi:10.1038/srep28284)
Supplement: Supplementary Information [file srep28284-s1.pdf]

Supplementary Information:

Genomics of high molecular weight plasmids isolated from an on-farm biopurification system

María C. Martini , Daniel Wibberg, Mauricio Lozano, Gonzalo Torres Tejerizo, Francisco J. Albicoro, Sebastian Jaenicke, Jan Dirk van Elsas, Alejandro Petroni, M. Pilar Garcillán-Barcia, Fernando de la Cruz, Andreas Schlüter, Alfred Pühler, Mariano Pistorio, Antonio Lagares*, María F. Del Papa*.

***Correspondence to authors**: floppy@biol.unlp.edu.ar, lagares@biol.unlp.edu.ar

Supplementary information:

Figure S1. IS families identified in the plasmid sequence data set.

Figure S2. Phylogeny of MOB_F relaxases of the plasmid dataset.

Figure S3. Phylogeny of MOB_Q relaxases of the plasmid dataset.

Figure S4. Phylogeny of MOB_V relaxases of the plasmid dataset.

Figure S5: a) Phylogeny of MOB_P relaxases of the plasmid dataset. **b)** Simplified representation of the tree presented in Fig._S5a.

Table S1. Replication genes identified in the plasmid sequence dataset.

Table S2. Stabilization and partition genes identified in the plasmid sequence dataset.

Table S3. Mobilization and conjugative transfer genes identified in the plasmid sequence dataset.

Table S4. Genes encoded on complete replicons. If the best BLAST hit corresponds to a hypothetical protein, the next closest hit (if available) featuring a functional annotation was assigned.

Table S5. List of antibiotic resistance genes identified in the plasmid sequence dataset.

Table S6. List of metal resistance genes identified in the plasmid sequence dataset.

Table S7. Genes potentially involved in degradation of specific pesticides as identified by BLASTp analyses.

Table S8. List of putative genes involved in degradation of xenobiotic and aromatic hydrocarbon compounds identified in the plasmid sequence dataset.

Table S9. Transposon and IS identified in the plasmid dataset **.**

Figure S1. IS families identified in the plasmid sequence data set.

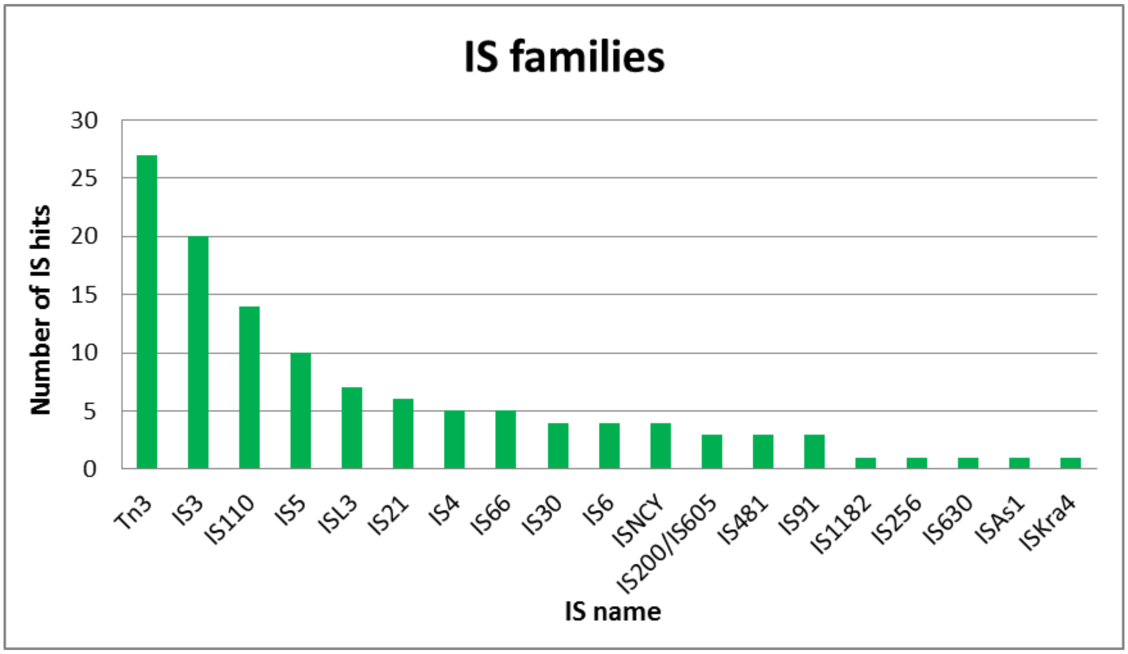

Figure S2. Phylogeny of MOB_F relaxases of the plasmid dataset. Highly related clusters are compressed and prototype members are indicated. MOB families and subfamilies defined in [22] are shadowed in grey. Only bootstrap values >50% are indicated in the corresponding nodes of the ML tree. The MOB_{F2} relaxase TraA of plasmid pNG2 was used as outgroup. MOB_F proteins of the plasmid dataset BJP_1037 and BJP_380) are boxed.

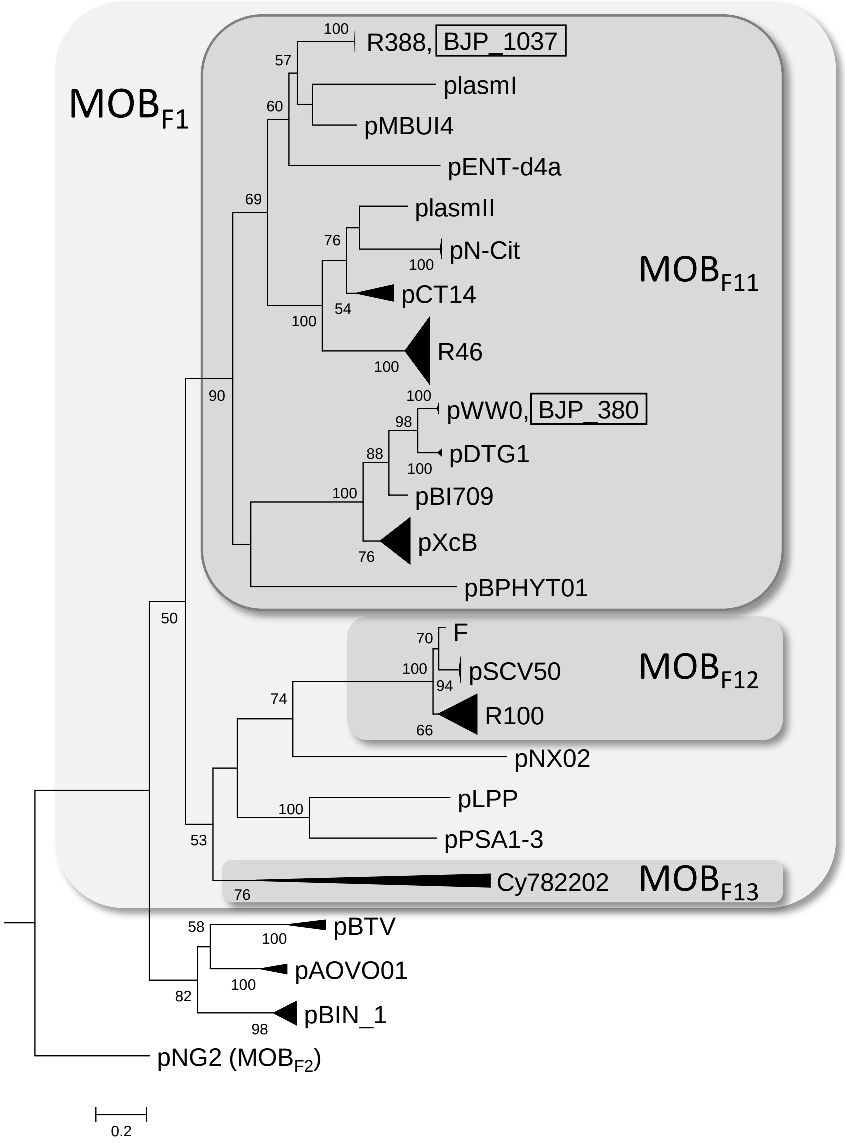

Figure S3. Phylogeny of MOB_Q relaxases of the plasmid dataset. Highly related clusters are compressed and prototype members are indicated. MOB families and subfamilies defined in Garcillán-Barcia (2009) are shadowed in grey. Only bootstrap values > 50% are indicated in the corresponding nodes of the ML tree. The MOB_{Q1} relaxase MobA of plasmid RSF1010 was used as outgroup. MOB_Q proteins of the plasmid dataset (AP_265, BJP_6246, BJP_4415 and BJP_1723) are boxed.

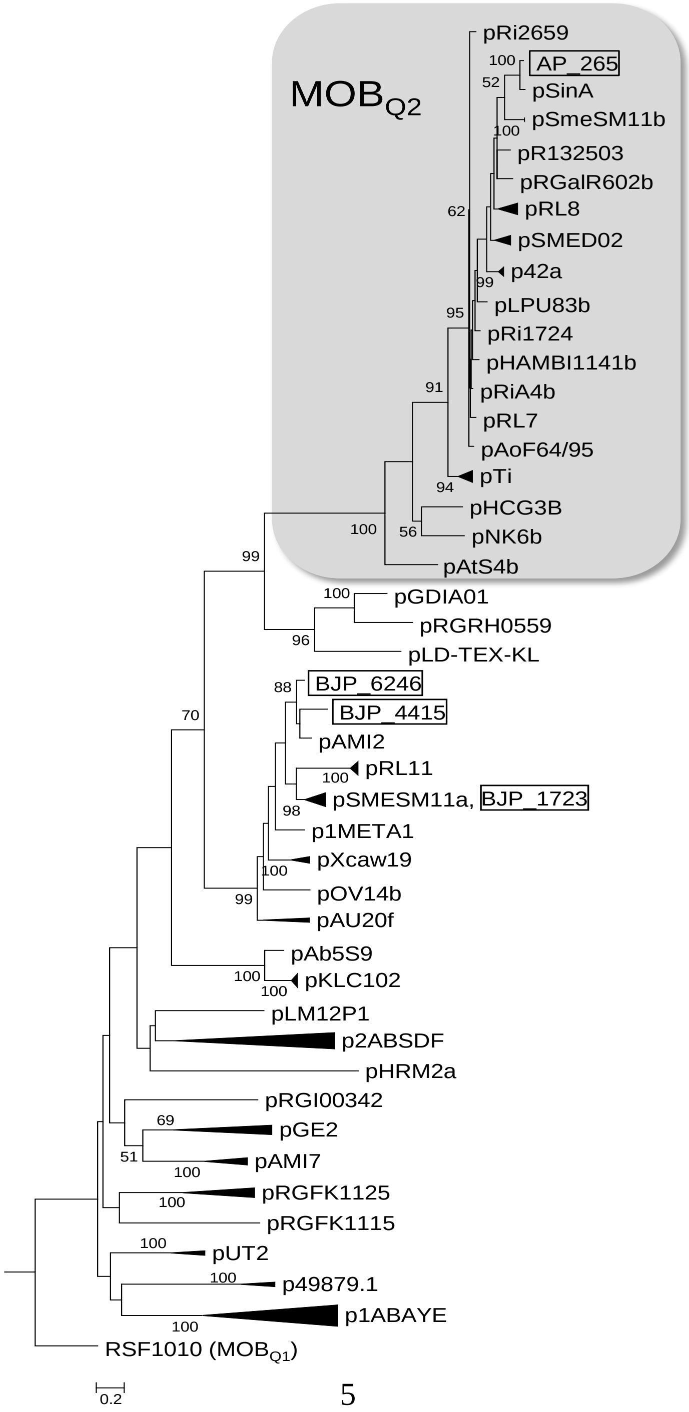

Figure S4. Phylogeny of MOB_V relaxases of the plasmid dataset. Highly related clusters are compressed and prototype members are indicated. MOB families and subfamilies defined in [22] are shadowed in grey. Only bootstrap values > 50% are indicated in the corresponding nodes of the ML tree. The MOB_{P1} relaxase TraI of plasmid RP4 was used as outgroup. The MOB_V protein of the plasmid dataset (pMC3_7) is boxed.

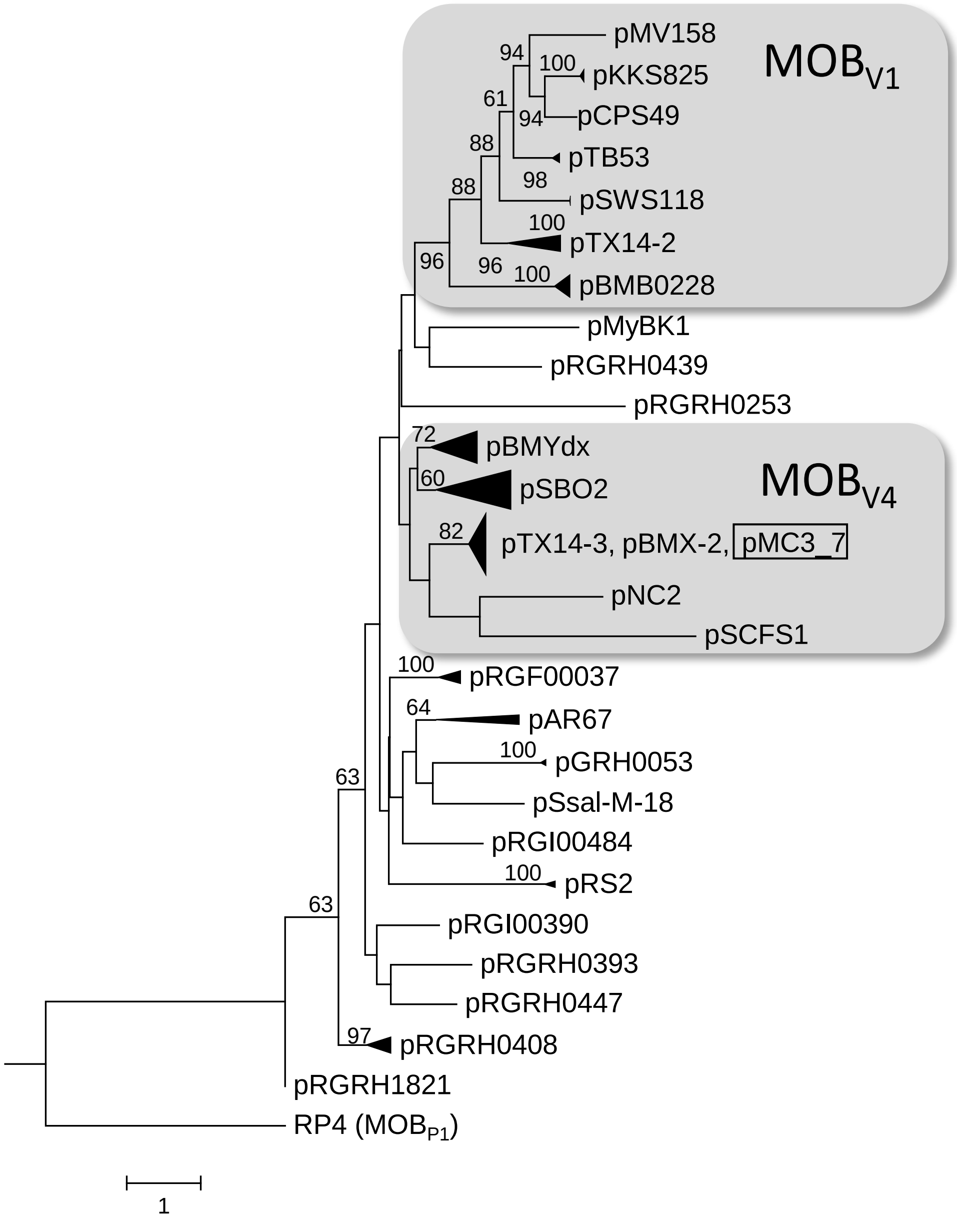

Figure S5: a) Phylogeny of MOB$_P$ relaxases of the plasmid dataset. Highly related clusters are compressed and prototype members are indicated. MOB families and subfamilies defined in [22] are shadowed in grey. Only bootstrap values > 50% are indicated in the corresponding nodes of the ML tree. The MOB$_V$ relaxase MobM of plasmid pMV158 was used as outgroup. The MOB$_P$ proteins of the plasmid dataset (BJP_1498, AP_669, BJP_441, BJP_847, BJP_634, BJP_2740, BJP_634, AP_394, AP_113, BJP_237, BJP_387, and pMC6_1) are boxed

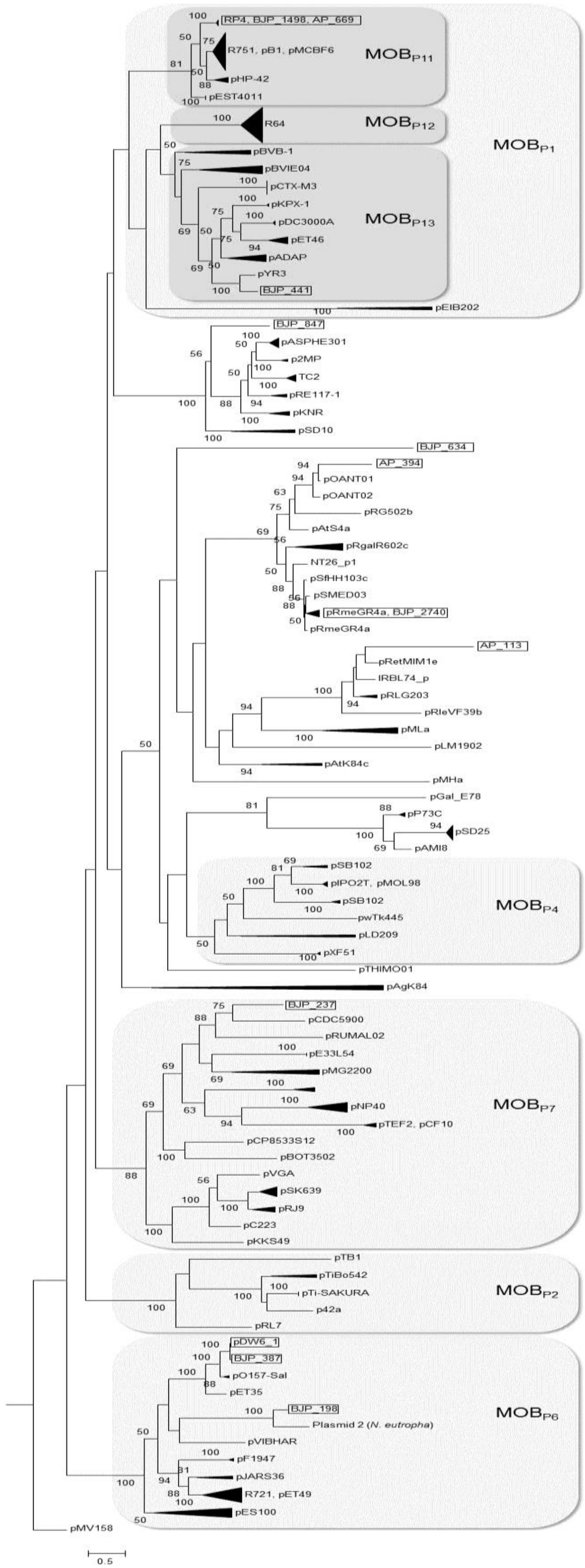

Figure S5. b) Phylogeny of MOB_P relaxases of the plasmid dataset. Simplified representation of the tree presented in Fig._S5a.

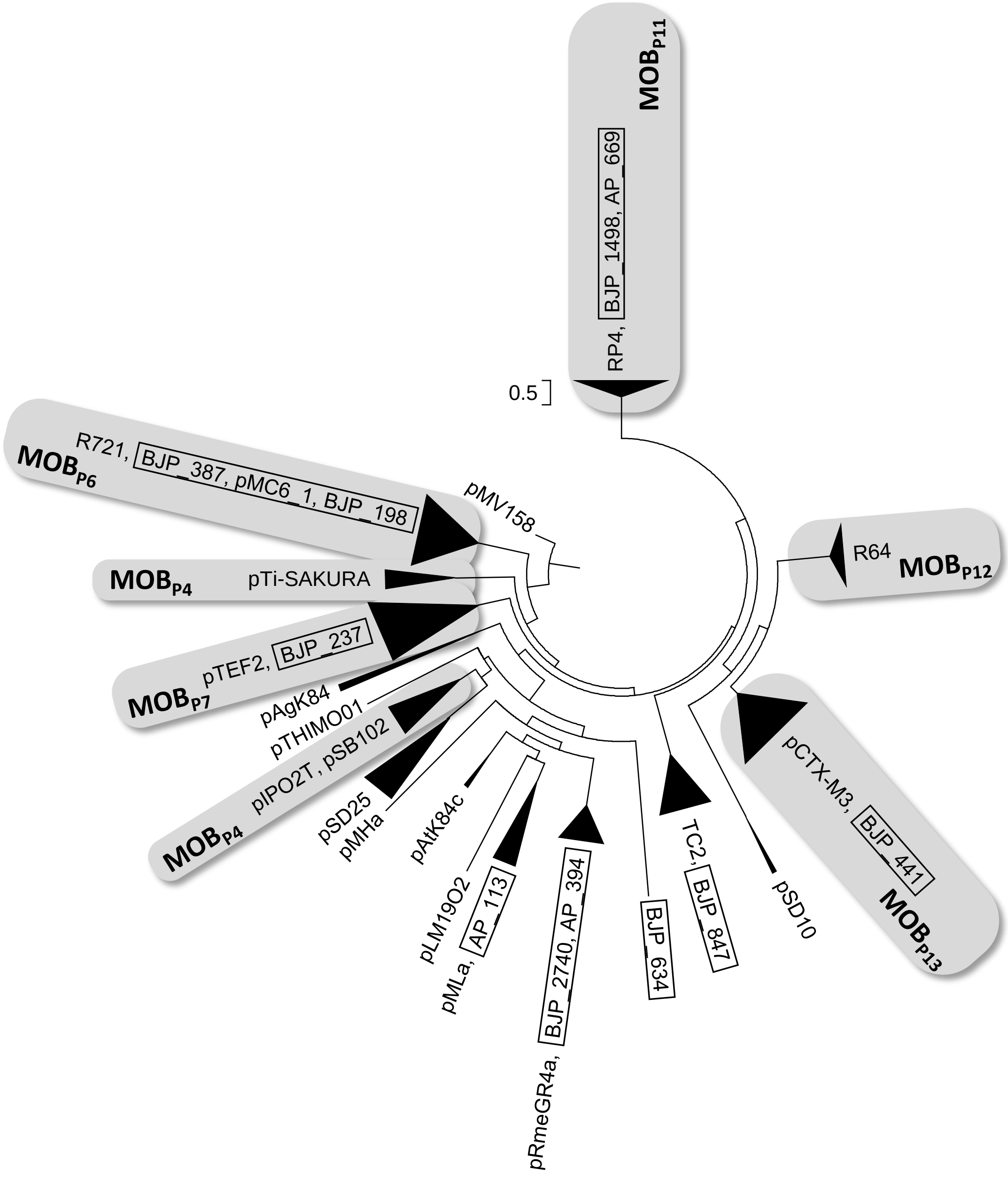

Table S1. Replication genes identified in the plasmid sequence dataset.

Name	Description (best BLASTp hit at NCBI)	Organism	Max score	Total score	Query cover	E value	Max ident	Accession number	Protein lenght (aminoacid)	Pfam ID	Pfam
BF_Rep1	single-stranded DNA-binding protein	*Legionella pneumophila*	132	132	91%	5,00E-36	50%	WP_027223436.1	139	SSB	pfam00436
BF_Rep2	single-stranded DNA-binding protein	*Legionella shakespearei*	130	130	97%	2,00E-35	49%	WP_018577135.1	139	SSB	pfam00436
BF_Rep3	single-stranded DNA-binding protein	*Ottowia thiooxydans*	229	229	83%	9,00E-74	96%	WP_028604336.1	135	SSB	pfam00436
BF_Rep4	single-strand DNA-binding protein	*Pseudomonas putida*	289	289	99%	1E-96	99%	WP_013100890.1	171	SSB	pfam00436
BF_Rep5	single-stranded DNA-binding protein	*Ochrobactrum intermedium*	317	317	99%	6E-108	93%	WP_025089737.1	164	SSB	pfam00436
BF_Rep6	single-stranded DNA-binding protein	*Microbacterium yannicii*	137	137	90%	5E-38	52%	WP_019181135.1	147	SSB	pfam00436
BF_Rep7	single-stranded DNA-binding protein, partial	*Pseudomonas syringae*	85.9	85.9	93%	2E-20	86%	EFW77643.1	46	SSB	pfam00436
BF_Rep8	single-strand DNA-binding protein	*Acinetobacter baumannii*	206	206	84%	1E-65	99%	WP_029642435.1	116	SSB	pfam00436
BF_Rep9	single-strand DNA-binding protein	*Pseudomonas putida*	112	112	98%	7E-29	59%	WP_013100890.1	91	SSB	pfam00436
BF_Rep10	single-stranded DNA-binding protein	*Salmonella enterica*	237	237	99%	5E-78	100%	WP_012196443.1	117	SSB	pfam00436
BF_Rep11	initiator RepB protein	*Providencia alcalifaciens*	303	303	88%	1,00E-99	64%	EUD00416.1	259	Rep_3	pfam01051
BF_Rep12	initiator RepB protein	*Salmonella enterica*	657	657	95%	0.0	99%	WP_000810866.1	354	Rep_3	pfam01051
BF_Rep13	hypothetical protein	*Pseudomonas putida*	509	509	99%	8,00E-180	100%	WP_011154372.1	288	Rep_3	pfam01051
BF_Rep14	hypothetical protein	*Pseudomonas taiwanensis*	808	808	99%	0.0	99%	ESW38296.1	427	Rep_3	pfam01051
BF_Rep15	initiator RepB protein	*Salmonella enterica*	658	658	90%	0.0	99%	WP_000810866.1	373	Rep_3	pfam01051

BF_Rep16	hypothetical protein	*Pseudomonas taiwanensis*	652	652	94%	0.0	71%	ESW38296.1	484	Rep_3	pfam01051
BF_Rep17	replication initiation factor	*Acidovorax citrulli*	322	322	86%	4,00E-104	56%	ABM32965.1	349	Rep_trans	pfam02486
BF_Rep18	hypothetical protein	*Streptococcus parasanguinis*	730	730	99%	0.0	97%	WP_023920333.1	370	Rep_trans	pfam02486
BF_Rep19	replication initiation factor	*Acidovorax delafieldii*	219	219	97%	2E-65	48%	WP_005794747.1	268	Rep_trans	pfam02486
BF_Rep20	hypothetical protein	*Streptococcus parasanguinis*	730	730	99%	0.0	97%	WP_023920333.1	370	Rep_trans	pfam02486
BF_Rep21	replication initiation factor	*Acidovorax citrulli*	322	322	86%	4E-104	56%	WP_011795501.1	350	Rep_trans	pfam02486
BF_Rep22	RepA: plasmid replication protein	uncultured bacterium	444	444	91%	9,00E-152	62%	WP_031943359.1	353	RepA_C	pfam04796
BF_Rep23	RepA: plasmid replication protein	uncultured bacterium	353	353	90%	1,00E-115	53%	WP_031943359.1	387	RepA_C	pfam04796
BF_Rep24	replication initiation protein	*Enterobacteriaceae*	635	635	99%	0.0	100%	WP_012196453.1	323	RepA_C	pfam04796
BF_Rep25	transcriptional regulator	*Pseudomonas aeruginosa*	318	318	99%	1E-103	100%	WP_011205825.1	503	KfrA_N	pfam11740
BF_Rep26	hypothetical protein	*Pseudomonas putida*	202	202	90%	4E-58	46%	WP_004574783.1	332	KfrA_N	pfam11740
BF_Rep27	hypothetical protein	*Serratia marcescens*	246	246	99%	9E-79	89%	KFF80982.1	203	KfrA_N	pfam11740
BF_Rep28	KfrA	*Salmonella enterica*	383	383	99%	1E-132	100%	WP_012443523.1	209	KfrA_N	pfam11740
BF_Rep29	hypothetical protein	*Pseudomonas* sp.	490	490	99%	3E-171	100%	WP_004577307.1	320	KfrA_N	pfam11740
BF_Rep30	Rep	*Bacillus pumilus*	464	464	99%	7,00E-162	94%	AAB71488.1	252	Rep_1	pfam01446
BF_Rep31	rep	*Bacillus pumilus*	507	507	98%	5,00E-180	98%	WP_011116799.1	252	Rep_1	pfam01446
BF_Rep32	replication protein A	*Pseudomonas* sp.	171	171	96%	8,00E-49	90%	WP_032881486.1	90	Replicase	pfam03090
BF_Rep33	RepA	*Aeromonas hydrophila*	266	266	99%	2,00E-86	99%	WP_014343694.1	130	Replicase	pfam03090
BF_Rep34	replication protein C	*Ochrobactrum rhizosphaerae*	716	716	99%	0.0	88%	WP_024899937.1	423	RP-C	pfam03428

BF_Rep35	Replication protein C	*Sinorhizobium meliloti*	781	781	99%	0.0	94%	WP_015241539.1	425	RP-C	pfam03428
BF_Rep36	hypothetical protein	*Ochrobactrum rhizosphaerae*	286	286	98%	9,00E-93	94%	WP_024899920.1	153	RP-C	pfam03428]
BF_Rep37	hypothetical protein	*Ochrobactrum rhizosphaerae*	254	254	87%	2E-80	95%	WP_036568391.1	153	RP-C	pfam03428
BF_Rep38	replication initiation protein	*Ochrobactrum anthropi*	687	687	99%	0.0	83%	WP_011982934.1	404	RP-C_C	pfam11800
BF_Rep39	replication initiation protein	*Ochrobactrum anthropi*	707	707	99%	0.0	89%	WP_011982878.1	409	RP-C_C	pfam11800
BF_Rep40	plasmid replication protein RepC	*Ochrobactrum rhizosphaerae*	698	698	99%	0.0	99%	WP_024897954.1	389	RP-C_C	pfam11800
BF_Rep41	replication initiation protein	*Sinorhizobium meliloti*	692	692	99%	0.0	94%	WP_017270868.1	399	RP-C_C	pfam11800
BF_Rep42	replication initiation protein	*Ochrobactrum anthropi*	409	409	99%	2,00E-139	88%	WP_011982878.1	236	RP-C_C	pfam11800
BF_Rep43	replication protein C	*Pseudomonas aeruginosa*	541	541	96%	0.0	96%	ETD44099.1	305	RepC	pfam06504
BF_Rep44	RepA	*Klebsiella pneumoniae*	337	337	96%	2,00E-113	79%	WP_012457107.1	237	IncFII_repA	pfam02387
BF_Rep45	DNA replication terminus site-binding protein	*Escherichia coli*	263	263	97%	2E-87	99%	WP_001444587.1	133	Ter	pfam05472
BF_Rep46	replication terminus site binding protein	*Pseudomonas sp.*	659	659	99%	0.0	100%	WP_011077913.1	323	Ter	pfam05472
BF_Rep47	TrfA family protein	*Thauera terpenica*	417	417	91%	6,00E-143	68%	WP_021250588.1	298	trfA	pfam07042
BF_Rep48	hypothetical protein	*Pseudomonadales*	741	741	99%	0.0	100%	WP_001082279.1	382	trfA	pfam07042
BF_Rep49	putative replicating protein RepA	*Agrobacterium tumefaciens*	491	491	100%	2,00E-173	100%	CDN96155.1	241	HTH_36	pfam13730
BF_Rep50	Rep	*Ochrobactrum intermedium*	395	395	100%	2,00E-137	99%	WP_006473476.1	194	HTH_36	pfam13730
BF_Rep51	replication protein	*Ochrobactrum rhizosphaerae*	660	660	99%	0.0	80%	WP_024899920.1	407	HTH_36	pfam13730
BF_Rep52	putative Rep	Plasmid pM3	348	348	99%	1,00E-119	100%	AAD46123.1	184	HTH_36	pfam13730

BF_Rep53	plasmid replication protein RepB	*Aeromonas hydrophila*	96.3	96.3	71%	6,00E-24	87%	WP_024944124.1	73	No putative conserved domains
BF_Rep54	hypothetical protein	*Bacillus cereus*	441	441	100%	3,00E-155	99%	WP_000170172.1	217	No putative conserved domains

Table S2. Stabilization and partition genes identified in the plasmid sequence dataset.

GenDB ID	Description (best BLASTp hit at NCBI)	Organism	Max score	Total score	Query cover	E value	Max ident	Accession number	Pfam ID	Pfam
BJP_459	cobyrinic acid a,c-diamide synthase	*Nitrosomonas eutropha*	317	317	99%	1E-104	65%	WP_011630666.1		
BJP_247	hypothetical protein	*Paenibacillus* sp.	496	496	99%	9,00E-176	94%	GAK43422.1		
BJP_9586	partitioning protein	*Salmonella enterica*	378	378	99%	7,00E-131	91%	YP_002332254.1		
BJP_65	partitioning protein	*Pseudomonas*	606	606	99%	0.0	83%	WP_004574780.1		
BJP_5270	hypothetical protein	*Sinorhizobium meliloti*	413	413	93%	5,00E-143	95%	WP_032492664.1		
AP_397	chromosome partitioning protein	*Ochrobactrum rhizosphaerae*	464	464	99%	2,00E-163	97%	WP_024899911.1		
BJP_928	partitioning protein	*Pseudomonas*	772	772	99%	0.0	100%	WP_004574780.1		
BJP_194	cobyrinic acid a,c-diamide synthase	*Nitrosomonas eutropha*	355	355	99%	8,00E-120	72%	WP_011630666.1		
AP_16	chromosome partitioning protein ParA	*Ochrobactrum anthropi*	792	792	99%	0.0	95%	EXL01616.1		
BJP_882	ParAF protein	*Frankia* sp.	166	166	83%	4,00E-47	47%	WP_032490082.1	CbiA	01656
BJP_6594	chromosome partitioning protein	*Cellulosimicrobium cellulans*	561	561	99%	0.0	90%	WP_024841573.1		
BJP_1333	chromosome partitioning protein ParA	*Ochrobactrum intermedium*	802	802	99%	0.0	96%	WP_025091213.1		
BJP_784	plasmid replication protein	*Thauera terpenica*	292	292	99%	2,00E-95	60%	WP_021250593.1		
AP_413	chromosome partitioning protein ParA	*Ochrobactrum rhizosphaerae*	782	782	99%	0.0	99%	WP_024899918.1		
AP_462	RepA	*Sinorhizobium* sp.	863	863	99%	0.0	99%	WP_015647669.1		
BJP_1253	hypothetical protein	*Klebsiella oxytoca*	431	431	99%	4,00E-151	97%	WP_023321975.1		
AP_658	IncC partitioning protein, short form	*Pseudomonas aeruginosa*	511	511	99%	0.0	100%	WP_011645034.1		
BJP_3797	hypothetical protein	*Pseudomonas veronii*	203	203	93%	2,00E-63	88%	WP_032804263.1		
BJP_361	putative ParA	Plasmid pM3	459	459	99%	6,00E-161	99%	AAD46128.1		

BJP_1033	StbB	*Enterobacteriaceae*	472	472	99%	8,00E-167	100%	WP_012443534.1		
BJP_384	plasmid stabilization protein	*Pseudomonas putida*	484	484	99%	2,00E-171	99%	WP_028691950.1		
BJP_2596	chromosome partitioning protein ParA	*Mesorhizobium alhagi*	555	555	99%	0.0	76%	WP_008840498.1		
BJP_624	hypothetical protein	*Pseudomonas putida*	774	774	99%	0.0	100%	WP_011154370.1		
BJP_201	hypothetical protein	*Nitrosomonas eutropha*	420	420	99%	3,00E-146	85%	WP_011630708.1		
BJP_5126	plasmid stability protein StbB	*Pseudomonas* sp.	355	355	99%	2,00E-120	70%	WP_032881477.1		
BJP_3572	putative plasmid stability/partitioning protein	*Providencia stuartii*	296	296	94%	4,00E-98	71%	WP_036944392.1		
BJP_732	hypothetical protein	*Nitrosomonas eutropha*	366	366	95%	6,00E-125	79%	WP_011630708.1		
BJP_554	hypothetical protein	*Burkholderia phytofirmans*	236	236	96%	2,00E-73	52%	WP_012430992.1		
AP_110	chromosome partitioning protein	*Ochrobactrum anthropi*	499	499	99%	4,00E-177	97%	WP_036588675.1		
BJP_6544	hypothetical protein	*Cellulosimicrobium cellulans*	312	312	99%	9,00E-105	97%	WP_034605270.1		
BJP_1829	plasmid stabilization protein	*Photorhabdus luminescens*	149	149	99%	6,00E-44	81%	WP_036780593.1	ParE_toxin	05016
BJP_3240	hypothetical protein	*Ochrobactrum* sp.	178	178	98%	3,00E-55	87%	WP_021585839.1		
	plasmid stabilization protein ParE	*Sinorhizobium meliloti*	116	116	94%	3,00E-31	55%	WP_017270034.1		
AP_402	RelE toxin	*Ochrobactrum*	196	196	98%	3,00E-62	100%	WP_011982866.1		
BJP_1830	hypothetical protein	*Photorhabdus luminescens*	239	239	98%	2,00E-77	82%	WP_036780591.1		
BJP_8969	hypothetical protein	*Xanthomonas campestris*	192	192	99%	3,00E-60	99%	WP_010378961.1		
BJP_8847	RelE toxin	*Aeromonas hydrophila*	177	177	94%	4,00E-55	97%	WP_017411297.1		
BJP_2512	hypothetical protein	*Pseudomonas aeruginosa*	116	116	98%	2,00E-31	96%	WP_023102300.1		
BJP_5251	hypothetical protein	*Serratia marcescens*	90.5	90.5	61%	4,00E-21	88%	WP_033645615.1		
BJP_8968	hypothetical protein	*Proteobacteria*	96.7	96.7	98%	7,00E-24	100%	WP_010378963.1	PtaRNA1	12703
BJP_8969	hypothetical protein	*Xanthomonas campestris*	192	192	99%	3,00E-60	99%	WP_010378961.1		

BJP_360	putative parB	Plasmid pM3	601	601	99%	0.0	98%	AAD46127.1		
pMC6_9	hypothetical protein	*Escherichia coli*	580	580	99%	0.0	99%	AEB22186.1		
BJP_395	hypothetical protein	*Escherichia coli*	580	580	99%	0.0	99%	AEB22186.1		
BJP_64	parB-like partition protein	*Pseudomonas*	476	476	98%	8,00E-165	69%	WP_023662218.1		
AP_783	chromosome partitioning protein ParB	*Sinorhizobium fredii*	1058	1058	99%	0.0	95%	WP_014330980.1		
BJP_257	parB-like partition protein	*Acidovorax citrulli*	432	485	76%	1,00E-133	46%	WP_011794736.1		
96	parB-like partition protein	*Acidovorax citrulli*	432	485	76%	1,00E-133	46%	WP_011794736.1		
BJP_96	hypothetical protein	*Pseudomonas*	341	341	97%	4,00E-112	56%	WP_023662083.1		
BJP_1833	hypothetical protein	*Xenophilus azovorans*	617	617	93%	0.0	56%	WP_038215288.1		
AP_461	chromosome partitioning protein ParB	*Ochrobactrum rhizosphaerae*	670	670	99%	0.0	99%	WP_024897953.1		
BJP_1332	chromosome partitioning protein ParB	*Ochrobactrum rhizosphaerae*	670	670	99%	0.0	99%	WP_024897953.1	ParBc	02195
AP_17	replication protein B	*Ochrobactrum anthropi*	558	558	99%	0.0	87%	WP_011983024.1		
BJP_3886	plasmid stabilization protein	*Sinorhizobium meliloti*	209	209	66%	1,00E-60	80%	CDH80775.1		
BJP_2597	chromosome partitioning protein ParB	*Mesorhizobium alhagi*	327	327	99%	2,00E-106	54%	WP_040589407.1		
BJP_192	parB-like partition proteins	*Nitrosomonas eutropha*	469	469	99%	9,00E-161	71%	WP_011630668.1		
BJP_461	parB-like partition proteins	*Nitrosomonas eutropha*	508	508	99%	1,00E-175	70%	WP_011630668.1		
AP_412	chromosome partitioning protein ParB	*Ochrobactrum rhizosphaerae*	662	662	99%	0.0	98%	WP_024899919.1		
BJP_783	plasmid-partitioning protein ParB	*Xylella fastidiosa*	228	228	71%	3,00E-67	50%	WP_010895251.1		
BJP_929	chromosome	*Pseudomonas*	694	694	99%	0.0	100%	WP_023662218.1		

	partitioning protein ParB								
BJP_619	putative replication/partitioning-related protein	*Pseudomonas putida*	586	586	99%	0.0	100%	WP_011600728.1		
BJP_1227	putative chromosome partitioning protein	*Pseudomonas putida*	1101	1101	97%	0.0	99%	WP_011600744.1		
AP_48	peptide transporter	*Rhizobiales*	1027	1027	99%	0.0	93%	WP_011982954.1		
AP_249	ParBC	*Sinorhizobium* sp.	1094	1094	97%	0.0	94%	WP_015647731.1		
BJP_2957	Rac prophage; conserved protein	*Escherichia coli*	181	181	98%	1,00E-56	100%	WP_001350510.1		
BJP_5052	hypothetical protein	*Cellulosimicrobium cellulans*	609	609	92%	0.0	87%	WP_034605271.1		
BJP_360	putative ParB	Plasmid pM3	601	601	99%	0.0	98%	AAD46127.1		
BJP_96	hypothetical protein	*Pseudomonas*	341	341	97%	4,00E-112	56%	WP_023662083.1		
BJP_783	plasmid partitioning protein parB	*Xylella fastidiosa*	228	228	71%	3,00E-67	50%	WP_010895251.1		
BJP_192	parB-like partition proteins	*Nitrosomonas eutropha*	469	469	99%	9,00E-161	71%	WP_011630668.1	KorB	08535
BJP_929	chromosome partitioning protein ParB	*Pseudomonas*	694	694	99%	0.0	100%	WP_023662218.1		
BJP_623	putative partitioning protein	*Pseudomonas putida*	657	657	99%	0.0	100%	WP_011600726.1		
BJP_1252	hypothetical protein	*Hafnia paralvei*	565	565	99%	0.0	83%	WP_039187478.1		
BJP_39	hypothetical protein	*Pseudomonas* sp.	543	543	99%	0.0	74%	WP_039614031.1		
BJP_2857	hypothetical protein	*Pseudomonas* sp.	676	676	99%	0.0	100%	WP_039614031.1	StbA	06406
BJP_10603	recombinase	*Yokenella regensburgei*	295	295	99%	2,00E-98	95%	WP_038255478.1		
BJP_5808	recombinase	*Kosakonia radicincitans*	261	261	99%	1,00E-84	93%	WP_007374358.1		
BJP_5697	recombinase	*Enterobacter cloacae*	159	159	98%	1,00E-45	94%	AHE72662.1		
BJP_805	DNA-binding protein, h-ns family	*Enterobacteriaceae*	188	188	98%	2,00E-59	99%	WP_012414174.1	Histone_HNS	00816
BJP_617	putative transcriptional regulator protein	*Pseudomonas putida*	244	244	99%	1,00E-80	100%	WP_011600730.1		

pMC6_34	DNA-binding protein	*Enterobacteriaceae*	217	217	99%	3,00E-69	100%	WP_000004210.1		
BJP_417	DNA-binding protein	*Enterobacteriaceae*	217	217	99%	3,00E-69	100%	WP_000004210.1		
BJP_777	histone	*Comamonas aquatica*	79.7	79.7	66%	2,00E-16	48%	EXU80104.1		
BJP_1146	transcriptional regulator	*Pseudomonas* sp.	182	182	99%	2,00E-56	86%	WP_007989755.1		
BJP_6898	transcriptional regulator	*Pseudomonas*	214	214	99%	1,00E-68	99%	WP_004577632.1		
pMC6_35	hypothetical protein	*Salmonella enterica*	226	226	99%	7,00E-74	99%	WP_000083087.1		
BJP_418	hypothetical protein	*Salmonella enterica*	226	226	99%	7,00E-74	99%	WP_000083087.1		
BJP_806	inner membrane protein	*Providencia rettgeri*	203	203	99%	4,00E-64	99%	WP_012414175.1		
BJP_5187	transcriptional regulator	*Pseudomonas* sp.	205	205	99%	3,00E-65	94%	WP_039615182.1		
BJP_5705	DNA-binding protein	*Shigella*	231	231	99%	5,00E-75	99%	WP_000115373.1		
BJP_10657	histone	*Ottowia thiooxydans*	136	136	98%	6,00E-39	79%	WP_028603560.1		
BJP_1332	hypothetical protein	*Ochrobactrum intermedium*	577	577	99%	0.0	85%	WP_025091214.1		
AP_461	chromosome partitioning protein ParB	*Ochrobactrum rhizosphaerae*	670	670	99%	0.0	99%	WP_024897953.1		
AP_412	chromosome partitioning protein ParB	*Ochrobactrum rhizosphaerae*	662	662	99%	0.0	98%	WP_024899919.1	RepB	07506
BJP_1533	Putative plasmid partitioning protein	*Pseudomonas putida*	361	361	99%	2,00E-122	85%	WP_004576822.1		
AP_17	replication protein B	*Ochrobactrum anthropi*	558	558	99%	0.0	87%	WP_011983024.1		
BJP_2597	chromosome partitioning protein ParB	*Mesorhizobium alhagi*	327	327	99%	2,00E-106	54%	WP_040589407.1		
BJP_427	antitoxin MazE	*Escherichia coli*	146	146	98%	4,00E-43	84%	WP_040062758.1		
BJP_770	hypothetical protein	*Ottowia thiooxydans*	94.0	94.0	86%	4,00E-22	68%	WP_036595224.1		
BJP_8860	antitoxin of the SohA(PrlF)-YhaV toxin-antitoxin system	*Enterobacteriaceae*	231	231	99%	6,00E-76	100%	WP_001307405.1	MazE_antitoxin	04014
BJP_2392	AbrB family transcriptional regulator	*Comamonas testosteroni*	141	141	85%	3,00E-41	100%	WP_003049911.1		
BJP_4543	AbrB family transcriptional regulator	*Pseudomonas fluorescens*	96.7	96.7	94%	7,00E-24	63%	WP_034096509.1		

BJP_9587	plasmid stabilization protein parB	*Pseudomonas aeruginosa*	112	112	98%	4,00E-30	74%	WP_034015122.1	ParG	09274
AP_456	hypothetical protein	*Sinorhizobium* sp.	288	288	99%	5,00E-97	100%	WP_015647674.1	PemK_toxin	02452
BJP_428	toxin MazF	*Escherichia coli*	197	197	99%	1,00E-62	85%	WP_000254736.1		
AP_599	hypothetical protein	*Xanthomonas*	172	172	97%	1,00E-52	79%	WP_005917856.1		
BJP_8130	antitoxin of the YafO-YafN toxin-antitoxin system	*Enterobacteriaceae*	197	197	98%	5,00E-63	100%	WP_000554758.1	PhdYeFM_antitox	02604
AP_46	prevent-host-death family protein	*Rhizobiales*	166	166	98%	7,00E-51	98%	WP_011982952.1		
BJP_764	hypothetical protein	*Xenophilus azovorans*	122	122	94%	6,00E-34	73%	WP_038214821.1		
AP_401	antitoxin	*Ochrobactrum rhizosphaerae*	159	159	98%	2,00E-48	99%	WP_024899915.1		
AP_457	prevent-host-death family protein	*chrobactrum rhizosphaerae*	154	154	98%	8,00E-46	100%	WP_024897956.1		
AP_257	hypothetical protein	*Ochrobactrum rhizosphaerae*	174	174	98%	3,00E-54	98%	WP_024897924.1		
AP_628	prevent-host-death protein	*Rhizobium* sp.	130	130	98%	3,00E-37	81%	WP_037200943.1		
BJP_2510	antitoxin	*Pseudomonas* sp.	159	159	98%	3,00E-48	95%	WP_021444922.1		
BJP_3551	prevent-host-death protein	*Paracoccus halophilus*	123	123	98%	9,00E-35	88%	WP_036744045.1		
BJP_760	toxin	*Verminephrobacter eiseniae*	238	238	81%	7,00E-72	56%	WP_011799399.1	Zeta_toxin	06414
BJP_241	hypothetical protein	*Paenibacillus peoriae*	332	332	83%	2,00E-112	85%	WP_039774836.1		
BJP_843	hypothetical protein	*Cellulosimicrobium cellulans*	318	318	70%	1,00E-103	87%	WP_034605280.1		
BJP_761	antitoxin	*Verminephrobacter aporrectodeae*	86.7	86.7	89%	1,00E-19	48%	WP_040891555.1		
BJP_1251	plasmid stability protein	*Hafnia paralvei*	158	158	99%	1,00E-46	60%	WP_039187476.1	Plasmid_stab_B	10784
BJP_10604	plasmid stabilization protein	*Yokenella regensburgei*	219	219	99%	7,00E-71	92%	WP_038255628.1		
BJP_489	plasmid maintenance system killer	*Nitrosomonas eutropha*	179	179	98%	8,00E-56	90%	WP_011630682.1	HigB-like_toxin	05015
BJP_490	XRE family plasmid maintenance system antidote protein	*Nitrosomonas eutropha*	185	185	81%	2,00E-57	88%	WP_011630683.1		

BJP_6484	ATPase	*Serratia marcescens*	356	356	99%	5,00E-120	100%	WP_016928441.1	ParA	10609
BJP_4288	antitoxin of YafQ-DinJ toxin-antitoxin system	*Enterobacteriaceae*	145	145	82%	9,00E-43	100%	WP_000729703.1	RelB	04221
BJP_165	hypothetical protein	*Pseudogulbenkiania ferrooxidans*	144	144	89%	1,00E-37	39%	WP_008955570.1		
BJP_7584	hypothetical protein	*Escherichia coli*	103	103	98%	3,00E-27	100%	WP_000554641.1	HOK_GEF	01848
BJP_2511	toxin of toxin-antitoxin (TA) system	*Pseudomonas* sp.	159	159	98%	2,00E-48	90%	WP_021444923.1	YoeB_toxin	06769

Table S3. Mobilisation and conjugative transfer genes found in the plasmid sequence dataset.

GenDB ID	Description (best BLASTp hit at NCBI)	Organism	Max score	Total score	Query cover	E value	Max ident	Accession number	Pfam ID	Pfam
BJP_441	hypothetical protein	*Yersinia ruckeri*	949	949	99%	0.0	73%	WP_004720847.1	Relaxase	03432
BJP_1498	hypothetical protein	*Sphingobium* sp.	1238	1238	99%	0.0	100%	KFL47726.1		
pMC6_1	relaxase	*Escherichia coli*	2439	2439	99%	0.0	99%	AEB22177.1		
BJP_387	relaxase	*Escherichia coli*	2439	2439	99%	0.0	99%	AEB22177.1		
AP_669	TraI	Cloning vector pDMK3	290	290	97%	3,00E-97	99%	ACO50702.1		
BJP_2740	conjugal transfer protein TraA	*Sinorhizobium fredii*	1597	1597	97%	0.0	91%	WP_014330857.1		
BJP_237	hypothetical protein	*Paenibacillus* sp.	835	835	99%	0.0	85%	GAK43394.1		
BJP_198	hypothetical protein	*Nitrosomonas eutropha*	1454	1454	99%	0.0	79%	WP_011630664.1		
BJP_634	endonuclease	*Rhodobacteraceae bacterium*	150	215	61%	3,00E-34	33%	WP_023849101.1		
AP_394	relaxase	*Ochrobactrum anthropi*	1788	1788	97%	0.0	94%	WP_011982859.1		
BJP_847	hypothetical protein	*Rhodococcus fascians*	246	246	78%	2,00E-70	45%	WP_032393683.1		
AP_113	hypothetical protein	*Agrobacterium tumefaciens*	1191	1191	99%	0.0	95%	WP_035226598.1		
BJP_796	TrwI	*Salmonella enterica*	402	402	82%	1,00E-135	99%	WP_012443542.1	TrbL	04610
BJP_369	type VI secretion protein	*Pseudomonas mosselii*	496	496	99%	1,00E-174	99%	WP_028691936.1		
BJP_4586	conjugal transfer protein TrbL	*Sinorhizobium medicae*	631	631	99%	0.0	99%	WP_018210781.1		
pMC6_14	conjugal transfer protein traA	*Salmonella enterica*	652	652	99%	0.0	100%	WP_000421118.1		
BJP_400	conjugal transfer protein traA	*Salmonella enterica*	652	652	99%	0.0	100%	WP_000421118.1		
AP_166	conjugal transfer protein TrbL	*Ochrobactrum anthropi*	556	556	99%	0.0	99%	WP_011982853.1		

AP_469	TrbL	*Sinorhizobium* sp.	492	575	82%	1,00E-169	98%	WP_015647759.1		
AP_94	TrbL/VirB6 plasmid conjugal transfer protein	*Rhizobium* sp.	565	565	99%	0.0	91%	WP_022557206.1		
BJP_724	TrbL/VirB6 plasmid conjugal transfer protein	*Nitrosomonas eutropha*	557	557	99%	0.0	78%	WP_011630700.1		
BJP_209	TrbL/VirB6 plasmid conjugal transfer protein	*Nitrosomonas eutropha*	463	463	98%	3,00E-158	63%	WP_011630700.1		
BJP_942	TrbL protein	*Proteobacteria*	407	506	87%	3,00E-132	100%	WP_011205794.1		
AP_672	conjugal transfer protein TrbL	*Ochrobactrum rhizosphaerae*	500	500	98%	3,00E-175	90%	WP_024896844.1		
BJP_569	hypothetical protein	*Xenophilus azovorans*	151	151	89%	4,00E-39	42%	WP_038214259.1		
BJP_4240	No significant similarity found.									
BJP_798	TrwJ	*Salmonella enterica*	455	455	99%	3,00E-160	99%	WP_012443544.1		
BJP_366	hypothetical protein	*Pseudomonas mosselii*	345	345	99%	5,00E-117	98%	WP_036986392.1		
AP_165	conjugal transfer protein	*Ochrobactrum anthropi*	423	423	99%	4,00E-147	99%	WP_011982852.1		
BJP_4588	conjugal transfer protein	*Sinorhizobium medicae*	355	355	99%	2,00E-121	98%	WP_018210782.1		
BJP_941	conjugal transfer protein TrbJ	*Proteobacteria*	322	405	69%	2,00E-105	100%	WP_011205792.1		
BJP_567	hypothetical protein	*Xenophilus azovorans*	219	219	80%	4,00E-67	53%	WP_038214266.1	T4SS	07996
pMC6_24	Putative conjugal transfer protein TraF	*Escherichia coli*	426	426	99%	7,00E-148	100%	AEB22148.1		
BJP_408	Putative conjugal transfer protein TraF	*Escherichia coli*	448	448	99%	3,00E-156	99%	AEB22148.1		
BJP_726	type IV secretion system family protein	*Nitrosomonas eutropha*	376	376	99%	6,00E-129	75%	WP_011630702.1		
AP_92	conjugal transfer protein	*Ochrobactrum anthropi*	472	472	99%	9,00E-166	94%	WP_036588726.1		
BJP_207	type IV secretion system family protein	*Nitrosomonas eutropha*	330	330	98%	1,00E-110	75%	WP_011630702.1		

BJP_4587	hypothetical protein	*Sinorhizobium medicae*	126	126	80%	2,00E-35	98%	WP_011971091.1		
BJP_1012	conjugal transfer protein	*Sinorhizobium meliloti*	58.5	58.5	91%	4,00E-09	95%	WP_028004229.1		
BJP_797	Eex (entry exclusion)	*Salmonella enterica*	158	158	98%	3,00E-48	100%	WP_012443543.1		
BJP_1010	lytic transglycosylase	*Sinorhizobium medicae*	741	741	99%	0.0	99%	WP_018210783.1		
AP_467	TrbJ	*Sinorhizobium* sp.	465	465	99%	5,00E-163	96%	WP_015647760.1		
AP_502	P-type DNA transfer protein VirB5	*Ochrobactrum* sp.	323	323	98%	5,00E-109	86%	WP_029928324.1		
BJP_1038	conjugal transfer protein	*Providencia rettgeri*	741	741	99%	0.0	100%	WP_012414161.1	T2SSE	00437
BJP_91	general secretion pathway protein E	*Pseudomonas putida*	696	696	95%	0.0	64%	WP_020190101.1		
BJP_2881	P-type DNA transfer ATPase VirB11	*Sinorhizobium meliloti*	652	652	99%	0.0	97%	WP_015241453.1		
BJP_373	type VI secretion protein	*Pseudomonas mosselii*	682	682	99%	0.0	97%	WP_028691940.1		
BJP_574	hypothetical protein	*Xenophilus azovorans*	410	410	83%	9,00E-138	59%	WP_038200604.1		
pMC6_29	secretion system protein E	*Enterobacteriaceae*	736	736	99%	0.0	100%	WP_001005926.1		
BJP_412	secretion system protein E	*Enterobacteriaceae*	736	736	99%	0.0	100%	WP_001005926.1		
BJP_713	type II/IV secretion system family protein	*Yersinia ruckeri*	636	636	98%	0.0	61%	KGA44905.1		
BJP_719	type II secretion system protein E	*Nitrosomonas eutropha*	665	665	99%	0.0	84%	WP_011630696.1		
BJP_705	conjugal transfer protein	*Yersinia ruckeri*	637	637	99%	0.0	85%	WP_004720814.1		
AP_170	type VI secretion protein	*Ochrobactrum anthropi*	672	672	96%	0.0	98%	WP_011982857.1		
AP_677	type IV secretion protein VirB11	*Ochrobactrum intermedium*	595	595	98%	0.0	86%	WP_025091591.1		
AP_463	conjugal transfer protein TrbB	*Rhizobium giardinii*	569	569	99%	0.0	99%	WP_018326380.1		
BJP_214	type II secretion system protein E	*Nitrosomonas eutropha*	694	694	99%	0.0	90%	WP_011630696.1		

BJP_4489	general secretory pathway component, cryptic	*Enterobacteriaceae*	1008	1008	99%	0.0	100%	WP_001219894.1
BJP_597	phytochrome sensor protein	*Pseudomonas*	1118	1118	99%	0.0	99%	WP_023662235.1
AP_6	Phosphonate ABC transporter ATP-binding protein	*Rhizobiales*	545	545	99%	0.0	100%	WP_006473175.1
AP_98	type VI secretion protein	*Ochrobactrum anthropi*	666	666	99%	0.0	98%	WP_036588717.1
BJP_4418	type II secretion system protein E	*Ottowia thiooxydans*	850	850	99%	0.0	90%	WP_028605692.1
BJP_8409	conjugal transfer ATPase TrbB	*Proteobacteria*	525	525	99%	0.0	100%	WP_011205784.1
BJP_4419	protein transporter hofB	*Escherichia coli*	872	872	99%	0.0	100%	WP_016241104.1
BJP_374	putative mating pair formation protein	*Pseudomonas mosselii*	536	536	99%	0.0	91%	WP_028691941.1
BJP_2966	phosphate ABC transporter ATP-binding protein	*Pseudomonas*	543	543	99%	0.0	99%	WP_003118432.1
BJP_2882	conjugal transfer protein TraG	*Sinorhizobium meliloti*	258	258	99%	3,00E-79	97%	WP_017265125.1
AP_223	PhnC	*Sinorhizobium* sp.	521	521	91%	0.0	100%	WP_015647706.1
BJP_9333	twitching motility protein PilT	*Ottowia thiooxydans*	224	224	99%	1,00E-69	96%	WP_028601942.1
BJP_9603	hypothetical protein	*Serratia marcescens*	332	332	99%	2,00E-110	99%	WP_016927991.1
AP_742	ABC transporter related protein	*Acidovorax* sp.	450	450	97%	1,00E-156	85%	EHL22328.1
BJP_2707	ABC transporter	*Comamonas* sp.	868	868	94%	0.0	81%	WP_027011233.1
BJP_10165	cell division protein FtsE	*Paenibacillus*	466	466	99%	2,00E-164	99%	WP_024631580.1
BJP_4134	sugar ABC transporter ATP-binding protein	*Delftia* sp.	320	320	96%	4,00E-106	65%	WP_034364857.1
BJP_10723	branched-chain amino acid ABC transporter ATP-binding protein	*Acidovorax* sp.	218	218	98%	9,00E-68	67%	WP_008905448.1

BJP_5636	nickel ABC transporter ATPase	*Enterobacteriaceae*	543	543	99%	0.0	100%	WP_000173631.1		
BJP_5231	conjugal transfer protein TraG	*Sinorhizobium* sp.	757	757	99%	0.0	96%	WP_037425374.1	T4SS-DNA_transf	02534
AP_171	conjugal transfer protein TraG	*Ochrobactrum rhizosphaerae*	1324	1324	99%	0.0	97%	WP_024899892.1		
BJP_9254	conjugal transfer protein TraG	*Paracoccus* sp.	501	501	98%	1,00E-171	73%	WP_010399298.1		
pMC6_30	conjugal transfer protein TraK	*Salmonella enterica*	1269	1269	99%	0.0	100%	WP_001177100.1		
BJP_413	conjugal transfer protein TraK	*Salmonella enterica*	1269	1269	99%	0.0	100%	WP_001177100.1		
BJP_718	TRAG family protein	*Nitrosomonas eutropha*	1216	1216	99%	0.0	90%	WP_011630695.1		
BJP_215	TRAG family protein	*Nitrosomonas eutropha*	1193	1193	99%	0.0	91%	WP_011630695.1		
BJP_231	hypothetical protein	*Paenibacillus* sp.	1047	1047	99%	0.0	92%	GAK43401.1		
BJP_7004	conjugal transfer protein TraG	*Agrobacterium tumefaciens*	791	791	99%	0.0	94%	WP_011199111.1		
BJP_744	hypothetical protein	*Leifsonia aquatica*	565	565	79%	0.0	62%	WP_039921200.1		
AP_262	conjugal transfer protein TraG	*Ochrobactrum rhizosphaerae*	1228	1228	97%	0.0	97%	WP_024897928.1		
BJP_2882	conjugal transfer protein TraG	*Sinorhizobium meliloti*	258	258	99%	3,00E-79	97%	WP_017265125.1		
BJP_2000	hypothetical protein	*Sphingobium*	235	235	97%	1,00E-76	85%	WP_020817825.1		
AP_99	conjugal transfer protein TraG	*Ochrobactrum anthropi*	1655	1655	99%	0.0	89%	WP_036588715.1		
BJP_630	conjugal transfer protein TraG	*Ochrobactrum rhizosphaerae*	120	203	98%	1,00E-28	75%	WP_024899892.1		
BJP_7005	hypothetical protein	*Rhizobium* sp.	75.9	75.9	92%	3,00E-16	65%	WP_018900447.1		
BJP_1499	conjugal transfer coupling protein TraG	*Proteobacteria*	1320	1320	99%	0.0	100%	WP_011205817.1		
BJP_7753	hypothetical protein	*Bacillus* sp.	493	675	96%	0.0	92%	WP_021483182.1		
BJP_381	trwB protein	*Pseudomonas putida group*	1017	1017	99%	0.0	99%	WP_028691947.1	TrwB_AAD_bind	10412

BJP_1036	TrwB	*Salmonella enterica*	949	949	99%	0.0	100%	WP_012443537.1		
BJP_137	conjugal transfer protein TraG	*Pseudomonas* sp.	1046	1046	99%	0.0	88%	WP_039614010.1		
BJP_1262	conjugal transfer protein TraG	*Pseudomonas*	987	987	99%	0.0	100%	WP_028699870.1		
BJP_558	hypothetical protein	*Xenophilus azovorans*	657	657	93%	0.0	64%	WP_038213399.1		
BJP_136	hypothetical protein	*Pseudomonas*	429	429	96%	4,00E-144	53%	WP_023662248.1		
BJP_2265	hypothetical protein	*Pseudomonas*	859	859	99%	0.0	100%	WP_023662248.1		
BJP_794	TrwG	*Salmonella enterica*	480	480	99%	5,00E-170	100%	WP_012443541.1		
BJP_370	mating pair formation protein	*Pseudomonas mosselii*	449	449	99%	5,00E-158	96%	WP_028691937.1		
BJP_722	VirB8 family protein	*Nitrosomonas eutropha*	311	311	98%	3,00E-103	66%	WP_011630699.1		
pMC6_26	conjugal transfer protein	*Enterobacteriaceae*	486	486	99%	1,00E-171	100%	WP_000476773.1		
BJP_409	conjugal transfer protein	*Enterobacteriaceae*	486	486	99%	1,00E-171	100%	WP_000476773.1		
AP_95	type VI secretion protein	*Agrobacterium tumefaciens*	448	448	99%	4,00E-157	96%	WP_035226642.1		
BJP_211	VirB8 family protein	*Nitrosomonas eutropha*	373	373	98%	8,00E-128	81%	WP_011630699.1	VirB8	04335
BJP_571	hypothetical protein	*Xenophilus azovorans*	258	258	69%	2,00E-80	51%	WP_038214256.1		
AP_470	TrbF	*Sinorhizobium* sp.	444	444	99%	5,00E-156	99%	WP_015647758.1		
AP_674	conjugal transfer protein TraJ	*Ochrobactrum anthropi*	369	369	99%	2,00E-126	92%	WP_010660269.1		
BJP_792	conjugal transfer protein	*Enterobacteriaceae*	92.0	92.0	93%	5,00E-21	100%	WP_012414163.1		
BJP_795	trwH	*Enterobacteriaceae*	95.5	95.5	97%	2,00E-24	98%	WP_012196427.1		
BJP_937	conjugal transfer protein TrbF	*Proteobacteria*	475	475	99%	2,00E-167	100%	WP_011205788.1		
BJP_2879	conjugal transfer protein TrbG	*Sinorhizobium* sp.	483	483	99%	8,00E-170	97%	WP_037425383.1	CagX	03524

BJP_1040	conjugal transfer protein	*Enterobacteriaceae*	399	399	99%	6,00E-138	100%	WP_012414163.1		
BJP_371	hypothetical protein	*Pseudomonas mosselii*	536	536	99%	0.0	99%	WP_028691938.1		
AP_168	conjugal transfer protein TrbG	*Ochrobactrum rhizosphaerae*	447	447	99%	8,00E-156	97%	WP_024899889.1		
pMC6_27	conjugal transfer protein TraH	*Enterobacteriaceae*	542	542	99%	0.0	100%	WP_000722128.1		
BJP_410	conjugal transfer protein TraH	*Enterobacteriaceae*	542	542	99%	0.0	100%	WP_000722128.1		
BJP_721	conjugal transfer protein TrbG/VirB9/CagX	*Nitrosomonas eutropha*	349	349	91%	7,00E-118	72%	WP_011630698.1		
BJP_212	conjugal transfer protein TrbG/VirB9/CagX	*Nitrosomonas eutropha*	395	395	91%	9,00E-136	82%	WP_011630698.1		
AP_471	Conjugal transfer proteinTrbG	*Rhizobium giardinii*	501	501	99%	4,00E-177	99%	WP_026203418.1		
BJP_572	hypothetical protein	*Xenophilus azovorans*	297	297	89%	5,00E-97	58%	WP_038214254.1		
AP_96	Conjugal transfer protein TrbG	*Ochrobactrum anthropi*	558	558	99%	0.0	97%	EXL02641.1		
AP_675	type IV secretion protein VirB9	*Ochrobactrum*	457	457	97%	2,00E-159	88%	WP_006468255.1		
BJP_938	conjugal transfer protein TrbG	*Proteobacteria*	579	579	99%	0.0	100%	WP_011645028.1		
BJP_792	conjugal transfer protein	*Enterobacteriaceae*	92.0	92.0	93%	5,00E-21	100%	WP_012414163.1		
BJP_799	TrwK	*Salmonella enterica*	1692	1692	99%	0.0	100%	WP_012443545.1		
BJP_365	putative mating pair formation protein	*Pseudomonas putida*	1809	1809	99%	0.0	97%	WP_011005966.1		
BJP_1009	type VI secretion protein	*Sinorhizobium medicae*	1624	1624	99%	0.0	99%	WP_018210784.1		
AP_163	type VI secretion protein	*Ochrobactrum anthropi*	1588	1588	99%	0.0	98%	WP_011982850.1	CagE_TrbE_VirB	03135
BJP_727	type IV secretion/conjugal transfer ATPase	*Nitrosomonas eutropha*	1505	1505	99%	0.0	89%	WP_011630703.1		
BJP_407	hypothetical protein	*Escherichia coli*	1684	1684	97%	0.0	99%	EQV00987.1		

pMC6_23	conjugal transfer protein traE	*Salmonella enterica*	1678	1678	97%	0.0	99%	WP_000106190.1		
BJP_206	type IV secretion/conjugal transfer ATPase	*Nitrosomonas eutropha*	1499	1499	99%	0.0	93%	WP_011630703.1		
AP_466	conjugal transfer protein TrbE	*Agrobacterium tumefaciens*	1603	1603	99%	0.0	98%	WP_035227071.1		
BJP_566	hypothetical protein	*Xenophilus azovorans*	785	785	98%	0.0	47%	WP_038214269.1		
AP_90	ATPase	*Ochrobactrum anthropi*	1637	1637	99%	0.0	98%	WP_036588729.1		
AP_503	transporter	*Ochrobactrum anthropi*	1397	1397	99%	0.0	88%	WP_010660274.1		
BJP_936	TrbE protein	*Ralstonia pickettii*	1543	1543	96%	0.0	89%	YP_006963073.1		
BJP_1037	TrwC	*Salmonella enterica*	1806	1806	98%	0.0	99%	WP_012443538.1	TrwC	08751
BJP_380	ATPase AAA	*Pseudomonas mosselii*	1969	1969	99%	0.0	99%	WP_028691946.1		
BJP_1039	conjugal transfer protein	*Providencia rettgeri*	684	684	99%	0.0	99%	WP_012414162.1	TrbI	03743
BJP_2880	conjugal transfer protein TrbI	*Sinorhizobium medicae*	642	642	99%	0.0	99%	WP_011971095.1		
AP_169	conjugal transfer protein TrbI	*Ochrobactrum anthropi*	674	674	99%	0.0	95%	WP_011982856.1		
AP_97	conjugal transfer protein TrbI	*Ochrobactrum anthropi*	659	659	99%	0.0	92%	WP_036588719.1		
pMC6_28	conjugal transfer protein traI	*Salmonella enterica*	643	643	99%	0.0	99%	WP_000999413.1		
BJP_411	conjugal transfer protein traI	*Salmonella enterica*	643	643	99%	0.0	99%	WP_000999413.1		
BJP_372	putative mating pair formation protein	*Pseudomonas mosselii*	726	726	99%	0.0	97%	WP_028691939.1		
BJP_573	hypothetical protein	*Xenophilus azovorans*	249	249	52%	1,00E-76	59%	WP_038200862.1		
BJP_143	hypothetical protein	*Pseudomonas putida*	482	482	99%	2,00E-165	65%	WP_016486961.1		
BJP_720	conjugation TrbI family protein	*Nitrosomonas eutropha*	494	494	99%	7,00E-171	72%	WP_011630697.1		
AP_676	type IV secretion protein VirB10	*Shinella zoogloeoides*	617	617	99%	0.0	78%	WP_023517144.1		
BJP_213	conjugation TrbI family	*Nitrosomonas eutropha*	504	504	99%	1,00E-	82%	WP_011630697.1		

	protein					174				
BJP_1268	conjugal transfer protein TraB	*Pseudomonas taiwanensis*	748	748	99%	0.0	100%	WP_023662243.1		
BJP_940	conjugal transfer protein TrbI	*Proteobacteria*	793	793	99%	0.0	100%	WP_011205791.1		
BJP_647	proQ/FINO family protein	*Ochrobactrum anthropi*	193	193	92%	2,00E-59	62%	AIK40840.1	ProQ	04352
BJP_5540	FinO	*Pectobacterium* sp.	100	100	50%	4,00E-23	54%	WP_014701991.1		
BJP_363	putative TrbC	Plasmid pM3	305	305	99%	2,00E-103	98%	AAD46129.1	TrbC	04956
BJP_1007	VIRB2 type IV secretion	*Sinorhizobium meliloti*	210	210	99%	2,00E-67	100%	WP_017265136.1		
BJP_801	TrwL	*Salmonella enterica*	170	170	99%	5,00E-52	100%	WP_012443547.1		
BJP_802	KorA	*Salmonella enterica*	181	181	98%	1,00E-56	100%	WP_012443548.1		
AP_161	VIRB2 type IV secretion fmaily protein	*Ochrobactrum anthropi*	182	182	99%	2,00E-56	97%	WP_011982848.1		
BJP_204	hypothetical protein	*Nitrosomonas eutropha*	145	145	82%	3,00E-42	89%	WP_011630705.1		
BJP_1006	lytic transglycosylase	*Sinorhizobium meliloti*	619	619	99%	0.0	92%	WP_013845317.1		
BJP_8408	conjugal transfer protein TrbC	*Proteobacteria*	175	175	84%	1,00E-52	100%	WP_011205785.1		
BJP_730	lytic transglycosylase, catalytic	*Nitrosomonas eutropha*	326	326	98%	1,00E-109	75%	WP_011630706.1		
BJP_203	lytic transglycosylase, catalytic	*Nitrosomonas eutropha*	328	328	89%	2,00E-110	79%	WP_011630706.1		
BJP_728	type IV secretory pathway, VirB3 family protein	*Nitrosomonas eutropha*	122	122	98%	2,00E-33	91%	WP_011630704.1		
BJP_800	trwM protein	*Salmonella enterica*	205	205	99%	1,00E-65	97%	WP_012196422.1	VirB3	05101
BJP_364	putative mating pair formation protein	*Pseudomonas putida*	192	192	98%	8,00E-61	100%	WP_011005967.1		
AP_162	type IV secretory pathway VirB3 family	*chrobactrum anthropi*	177	177	99%	6,00E-55	99%	WP_011982849.1		

	protein									
BJP_205	type IV secretory pathway, VirB3 family protein	*Nitrosomonas eutropha*	174	174	99%	2,00E-53	93%	WP_011630704.1		
BJP_728	type IV secretory pathway, VirB3 family protein	*Nitrosomonas eutropha*	122	122	98%	2,00E-33	91%	WP_011630704.1		
BJP_1008	type IV secretory pathway, VirB3	*Sinorhizobium meliloti*	200	200	99%	4,00E-64	100%	WP_020479309.1		
AP_465	conjugal transfer protein TrbD	*Ochrobactrum rhizosphaerae*	162	162	99%	8,00E-49	98%	WP_024897950.1		
AP_88	conjugal transfer protein TrbC	*Ochrobactrum anthropi*	229	229	99%	1,00E-74	100%	EXL02649.1		
AP_504	type IV secretion protein VirB3	*Ochrobactrum*	211	211	99%	1,00E-67	89%	WP_006468248.1		
BJP_1723	Ti-type conjugative transfer relaxase TraA	*Rhizobium leguminosarum*	1864	1864	94%	0.0	81%	EJC77937.1	MobA_MobL	03389
BJP_6246	conjugal transfer protein TraA	*Ochrobactrum intermedium*	789	789	99%	0.0	99%	WP_036566255.1		
AP_265	Dtr system oriT relaxase	*Agrobacterium tumefaciens*	2058	2058	99%	0.0	95%	WP_035227113.1		
BJP_4415	conjugal transfer protein TraA	*Agrobacterium tumefaciens*	1155	1155	99%	0.0	78%	WP_026109554.1		
BJP_2253	TraG family bacterial conjugation protein	*Arthrobacter arilaitensis*	564	564	80%	0.0	64%	WP_013350309.1	TraG-D_C	12696
BJP_444	hypothetical protein	*Yersinia ruckeri*	1315	1315	99%	0.0	81%	EEP98795.1		
BJP_744	hypothetical protein	*Leifsonia aquatica*	565	565	79%	0.0	62%	WP_039921200.1		
BJP_3931	ATPase involved in chromosome partitioning-like protein	*Rhizobium* sp.	377	377	99%	1,00E-129	84%	CCF22397.1	PRKCSH	07915
BJP_152	hypothetical protein	*Pseudomonas*	1445	1445	98%	0.0	67%	WP_023662237.1	TraG_N	07916
BJP_4168	hypothetical protein	*Pseudomonas*	1269	1269	99%	0.0	100%	WP_023662237.1		
BJP_2547	membrane protein	*Parvibaculum lavamentivorans*	947	947	99%	0.0	95%	WP_012112346.1		
BJP_6103	2,3-dehydroadipyl-CoA hydratase	*Serratia marcescens*	378	378	99%	5,00E-129	96%	WP_033639967.1		

BJP_588	hypothetical protein	*Pseudomonas* sp.	853	853	97%	0.0	90%	WP_039614051.1		
BJP_84	conjugal transfer protein traN	*Pseudomonas syringae*	753	753	95%	0.0	73%	WP_032708792.1	TraN	06986
BJP_1942	mating pair stabilization protein	*Pseudomonas putida*	1782	1782	99%	0.0	100%	WP_033040477.1		
BJP_150	conjugal transfer protein	*Pseudomonas monteilii*	748	748	99%	0.0	78%	WP_028699874.1		
BJP_7057	conjugal transfer protein traH	*Pseudomonas*	225	225	99%	5,00E-68	99%	WP_023662238.1	TraH	06122
BJP_4167	conjugal transfer protein traH	*Pseudomonas*	486	486	99%	2,00E-168	100%	WP_023662238.1		
BJP_7055	conjugal transfer protein traH	*Pseudomonas*	138	138	55%	9,00E-36	100%	WP_023662238.1		
BJP_790	hypothetical protein	*Thauera terpenica*	160	160	80%	1,00E-45	54%	WP_021250285.1		
BJP_458	TrbM family protein	*Nitrosomonas eutropha*	248	248	83%	5,00E-80	70%	WP_011630665.1		
BJP_195	TrbM family protein	*Nitrosomonas eutropha*	281	281	83%	4,00E-93	74%	WP_011630665.1		
BJP_375	hypothetical protein	*Pseudomonas mosselii*	197	197	99%	2,00E-62	96%	WP_028691942.1		
BJP_804	KikA	*Salmonella enterica*	413	413	99%	8,00E-145	99%	WP_012443550.1		
pMC6_31	arginine transporter	*Enterobacteriaceae*	204	204	99%	1,00E-64	100%	WP_000848516.1		
BJP_414	arginine transporter	*Enterobacteriaceae*	204	204	99%	1,00E-64	100%	WP_000848516.1	TrbM	07424
BJP_376	endonuclease	*Pseudomonas mosselii*	352	352	95%	2,00E-120	94%	WP_028691943.1		
BJP_943	conjugal transfer protein TrbM	*Pseudomonas aeruginosa*	373	373	99%	7,00E-129	100%	WP_011205795.1		
AP_507	hypothetical protein	*Ochrobactrum rhizosphaerae*	276	276	99%	3,00E-91	72%	WP_024899142.1		
BJP_791	hypothetical protein	*Alicycliphilus* sp. *CRZ1*	244	244	99%	5,00E-80	81%	WP_019372870.1		
pMC6_32	hypothetical protein	*Salmonella enterica*	274	274	99%	8,00E-92	100%	WP_000368397.1		
BJP_415	hypothetical protein	*Salmonella enterica*	274	274	99%	4,00E-	100%	YP_002144947.1		

						92				
AP_155	hypothetical protein	*Ochrobactrum anthropi*	380	380	99%	1,00E-132	97%	YP_001373086.1		
AP_103	hypothetical protein	*Ochrobactrum anthropi*	332	332	99%	7,00E-114	92%	WP_036588691.1		
BJP_146	F-pilin subunit assembly into extended F pili	*Pseudomonas monteilii*	1488	1488	99%	0.0	85%	WP_028699872.1	TraC_F_IV	11130
BJP_1271	sex pilus assembly protein	*Pseudomonas*	567	567	97%	0.0	99%	WP_023662242.1		
BJP_227	TrsE protein	*Paenibacillus peoriae*	1473	1473	99%	0.0	96%	WP_010349924.1		
BJP_734	mobilisation protein	*Nitrosomonas eutropha*	215	215	99%	4,00E-69	85%	WP_011630663.1	MobC	05713
pMC6_2	hypothetical protein	*Salmonella enterica*	258	258	99%	9,00E-86	100%	WP_001157192.1		
BJP_388	hypothetical protein	*Salmonella enterica*	258	258	99%	9,00E-86	100%	WP_001157192.1		
BJP_199	hypothetical protein	*Nitrosomonas eutropha*	214	214	99%	8,00E-69	83%	WP_011630663.1		
AP_395	mobilisation protein	*Ochrobactrum anthropi*	255	255	99%	4,00E-84	95%	WP_011982860.1		
BJP_2739	mobilization protein	*Sinorhizobium/Ensifer group*	148	148	98%	1,00E-43	97%	WP_034859608.1		
BJP_848	putative mobilisation protein	*Propionibacterium freudenreichii*	122	122	94%	3,00E-32	59%	CEH05296.1		
BJP_454	hypothetical protein	*Nitrosomonas eutropha*	1189	1189	99%	0.0	77%	WP_011630664.1		
BJP_5273	mobilisation protein	*Sinorhizobium fredii*	52.8	52.8	95%	3,00E-07	82%	WP_037461848.1		
BJP_140	hypothetical protein	*Pseudomonas*	137	137	97%	2,00E-39	90%	WP_016486958.1	TraL	07178
BJP_1265	hypothetical protein	*Pseudomonas*	149	149	98%	6,00E-44	99%	WP_016486958.1		
BJP_139	hypothetical protein	*Pseudomonas putida*	214	214	87%	2,00E-66	66%	WP_020190088.1		
BJP_1264	flagellar motor protein MotB	*Pseudomonas*	349	349	99%	2,00E-119	100%	WP_023662245.1		

BJP_141	hypothetical protein	*Pseudomonas*	314	314	99%	3,00E-105	69%	WP_016486959.1	TraE	05309
BJP_1266	hypothetical protein	*Pseudomonas*	435	435	99%	4,00E-153	100%	WP_016486959.1		
BJP_894	mobilization protein	*Bacillus cereus*	978	978	99%	0.0	98%	WP_000901534.1	Mob_Pre	01076
pMC3_7	mobilization protein	*Bacillus cereus*	976	976	99%	0.0	98%	WP_000901534.1		
AP_477	hypothetical protein	*Rhizobium giardinii*	191	191	70%	1,00E-59	95%	WP_018326248.1	Prok-TraM	09228
AP_478	TraR	*Rhizobiaceae*	480	480	99%	4,00E-170	100%	WP_015647751.1		
AP_263	conjugal transfer protein TraD	*Agrobacterium tumefaciens*	129	129	98%	7,00E-37	94%	WP_035227115.1	TraD	06412
AP_264	conjugal transfer protein TraC	*Agrobacterium tumefaciens*	135	135	87%	1,00E-38	93%	WP_035227185.1	TraC	07820
BJP_7783	MbeD/MobD	*Serratia*	137	137	89%	6,00E-39	100%	WP_004928976.1	MbeD_MobD	04899
BJP_6195	mobilisation protein	*Proteobacteria*	149	149	98%	2,00E-44	99%	WP_000137445.1		
BJP_7784	membrane protein	*Serratia marcescens*	277	277	99%	1,00E-92	100%	WP_033639556.1		
BJP_4419	protein transporter hofB	*Escherichia coli*	872	872	99%	0.0	100%	WP_016241104.1	T2SSE_N	05157
BJP_144	membrane lipoprotein lipid attachment site	*Pseudomonas* sp.	284	284	81%	8,00E-94	82%	WP_023383789.1		
BJP_1269	membrane lipoprotein lipid attachment site	*Pseudomonas* sp.	335	335	88%	4,00E-114	96%	WP_023383789.1	TraV	09676
BJP_149	conjugal transfer protein	*Pseudomonas putida*	487	487	91%	6,00E-170	84%	WP_029615335.1	TraF	13728
BJP_7056	conjugal transfer protein	*Pseudomonas*	491	491	99%	2,00E-174	100%	WP_031325012.1		

Table S4. Genes encoded on complete replicons. If the best BLAST hit corresponds to a hypothetical protein, the next closest hit (if available) featuring a functional annotation was assigned.

Replicon name	CDS num ber	Description (best BLASTp hit at NCBI)	Organism	Query lenght	Subject lenght	Query coverage	e-value	Max ident	Accession number	Observations
**pMC1**	1	No significant similarity found								
**pMC1**	2	hypothetical protein	*Ochrobactrum anthropi*	127	127	98%	5,00E-36	79%	WP_036587968.1	phage related protein
**pMC1**	3	hypothetical protein	*Ochrobactrum rhizosphaerae*	117	117	98%	2,00E-32	80%	WP_036568669.1	phage-related hypothetical protein
**pMC1**	4	hypothetical protein	*Ochrobactrum rhizosphaerae*	115	115	96%	6,00E-31	67%	WP_024900284.1	
**pMC1**	5	C-5 cytosine-specific DNA methylase	*Ochrobactrum rhizosphaerae*	951	951	99%	0.0	81%	WP_024900288.1	
**pMC1**	6	hypothetical protein	*Ochrobactrum rhizosphaerae*	72.4	72.4	42%	2,00E-13	58%	WP_024899497.1	
**pMC1**	7	repressor protein C	*Ochrobactrum intermedium*	298	298	97%	2,00E-98	60%	WP_036565897.1	
**pMC1**	8	hypothetical protein	*Mesorhizobium* sp.	76.3	76.3	48%	2,00E-15	59%	ESZ60509.1	molecular chaperone
**pMC1**	9	hypothetical protein	*Hoeflea phototrophica*	190	190	96%	3,00E-58	60%	WP_007196931.1	
**pMC1**	10	hypothetical protein	*Ochrobactrum rhizosphaerae*	91.7	91.7	77%	4,00E-22	77%	WP_024900293.1	
**pMC1**	11	hypothetical protein	*chrobactrum rhizosphaerae*	162	162	98%	5,00E-49	80%	WP_024900294.1	
**pMC1**	12	hypothetical protein	*Ochrobactrum rhizosphaerae*	404	404	78%	7,00E-139	78%	WP_036568721.1	
**pMC1**	13	hypothetical protein	*Ochrobactrum rhizosphaerae*	125	125	99%	7,00E-32	41%	WP_024900296.1	chromosomal replication initiator protein DnaA. Pfam08299

**pMC1**	14	No significant similarity found								
**pMC1**	15	DNA methylase	*Ochrobactrum rhizosphaerae*	395	395	77%	6,00E-136	84%	WP_036568723.1	
**pMC1**	16	hypothetical protein	*Ochrobactrum rhizosphaerae*	146	146	97%	1,00E-42	78%	WP_024900298.1	
**pMC1**	17	hypothetical protein	*Ochrobactrum rhizosphaerae*	253	253	99%	1,00E-82	75%	WP_024900299.1	
**pMC1**	18	hypothetical protein	*Ochrobactrum anthropi*	436	436	99%	1,00E-147	58%	WP_012092184.1	replication protein Paenibacillus sp.
**pMC1**	19	hypothetical protein	*Ochrobactrum rhizosphaerae*	242	242	98%	8,00E-77	62%	WP_024899506.1	NusG antitermination factor
**pMC1**	20	No significant similarity found								
**pMC1**	21	hypothetical protein	*Ochrobactrum rhizosphaerae*	185	185	77%	2,00E-57	89%	WP_036568726.1	endonuclease
**pMC1**	22	hypothetical protein	*Sinorhizobium* sp.	81.3	81.3	85%	3,00E-18	72%	WP_037421955.1	
**pMC1**	23	hypothetical protein	*Starkeya novella*	105	105	64%	5,00E-25	51%	WP_013164853.1	resolvase helix-turn-helix domain protein
**pMC1**	24	terminase	*Sphingobium* sp.	584	584	94%	0.0	54%	WP_037530719.1	phage terminase, large subunit
**pMC1**	25	portal protein	*Ochrobactrum rhizosphaerae*	862	862	95%	0.0	97%	WP_024900305.1	
**pMC1**	26	peptidase	*Ochrobactrum rhizosphaerae*	502	502	99%	3,00E-177	95%	WP_024900306.1	
**pMC1**	27	nucleoid-structuring protein H-NS	*Ochrobactrum rhizosphaerae*	793	793	99%	0.0	90%	WP_024900307.1	
**pMC1**	28	hypothetical protein	*Ochrobactrum rhizosphaerae*	237	237	99%	1,00E-75	72%	WP_024900308.1	flagellar hook-associated protein 2
**pMC1**	29	hypothetical protein	*Ochrobactrum rhizosphaerae*	199	199	48%	1,00E-61	97%	WP_036568728.1	phage protein
**pMC1**	30	phage head-tail adapter protein	*Ochrobactrum rhizosphaerae*	213	213	73%	8,00E-68	91%	WP_024900310.1	
**pMC1**	31	hypothetical protein	*Ochrobactrum rhizosphaerae*	261	261	83%	3,00E-86	90%	WP_024900312.1	gene transfer agent
**pMC1**	32	hypothetical protein	*Ochrobactrum rhizosphaerae*	291	291	98%	3,00E-98	95%	WP_024900314.1	phage major tail protein

**pMC1**	33	hypothetical protein	*Ochrobactrum* sp.	238	238	99%	1,00E-77	85%	WP_036596028.1	
**pMC1**	34	hypothetical protein	*Ochrobactrum rhizosphaerae*	259	259	17%	2,00E-76	96%	WP_036568734.1	phage-related minor tail protein
**pMC1**	35	hypothetical protein	*Ochrobactrum anthropi*	60.1	60.1	61%	3,00E-10	76%	WP_041544905.1	
**pMC1**	36	hypothetical protein	*Ochrobactrum anthropi*	79.0	79.0	95%	1,00E-17	84%	WP_041544905.1	
**pMC1**	37	hypothetical protein	*Ochrobactrum rhizosphaerae*	394	394	99%	3,00E-136	92%	WP_024900317.1	
**pMC1**	38	hypothetical protein	*Ochrobactrum rhizosphaerae*	379	379	99%	2,00E-131	93%	WP_024900318.1	phage protein
**pMC1**	39	hypothetical protein	*Ochrobactrum rhizosphaerae*	223	223	99%	5,00E-72	80%	WP_024900319.1	phage protein
**pMC1**	40	fibronectin	*Ochrobactrum rhizosphaerae*	988	988	91%	0.0	86%	WP_024900320.1	
**pMC1**	41	hypothetical protein	*Ochrobactrum rhizosphaerae*	275	275	32%	3,00E-79	65%	WP_036568737.1	chaperone of endosialidase family protein
**pMC1**	42	hypothetical protein	*Ochrobactrum intermedium*	248	248	42%	1,00E-68	43%	WP_031353039.1	glycosyl transferase family 2
**pMC1**	43	hypothetical protein	*Mesorhizobium loti*	277	277	98%	1,00E-89	54%	WP_010915306.1	type 11 methyltransferase
**pMC1**	44	No significant similarity found								
**pMC1**	45	hypothetical protein	*Ochrobactrum rhizosphaerae*	437	437	93%	7,00E-152	85%	WP_036568757.1	membrane protein
**pMC1**	46	hypothetical protein	*Ochrobactrum intermedium*	105	105	96%	7,00E-27	58%	WP_025089589.1	conserved exported protein
**pMC1**	47	hypothetical protein	*Ochrobactrum anthropi*	138	138	91%	1,00E-39	73%	WP_029376117.1	
**pMC1**	**48**	hypothetical protein	*Agrobacterium vitis S4*	125	125	98%	3,00E-33	40%	ACM36644.1	antitoxin HicB
**pMC1**	**49**	hypothetical protein	*Devosia* sp.	89.7	89.7	97%	4,00E-21	51%	WP_035102542.1	HicA-related toxin-antitoxin protein
**pMC1**	50	hypothetical protein	*Sinorhizobium meliloti*	182	182	81%	8,00E-52	38%	WP_017274933.1	cobalamin biosynthesis protein CobW

**pMC1**	51	integrase	*Proteobacteria*	487	487	99%	7,00E-167	60%	WP_035026333.1	
**pMC2**	1	hypothetical protein	*Delftia* sp.	81.6	81.6	93%	7,00E-18	57%	WP_013803144.1	
**pMC2**	2	hypothetical protein	*Acidovorax radicis*	50.1	50.1	93%	4,00E-06	46%	WP_010465941.1	Phosphoribosylformylglycinamidine synthase
**pMC2**	3	hypothetical protein	*Burkholderia xenovorans*	192	192	94%	3,00E-59	62%	WP_011487586.1	RNA binding protein
**pMC2**	4	hypothetical protein	*Alicycliphilus denitrificans*	45.4	45.4	56%	3,00E-04	45%	WP_013516292.1	aminotransferase
**pMC2**	5	hypothetical protein	*Polaromonas* sp.	60.8	60.8	85%	7,00E-10	40%	WP_007874681.1	putative cardiotoxin-5V
**pMC2**	6	parB-like partition protein	*Acidovorax citrulli*	432	485	76%	1,00E-133	46%	WP_011794736.1	ParB-like nuclease domain
**pMC2**	7	No significant similarity found								
**pMC2**	8	No significant similarity found								
**pMC2**	9	No significant similarity found								
**pMC2**	10	No significant similarity found								
**pMC2**	11	hypothetical protein	*Delftia acidovorans*	46.2	46.2	52%	3,00E-04	38%	WP_016447887.1	signal transduction histidine kinase
**pMC2**	12	hypothetical protein	*Mesorhizobium* sp.	102	102	84%	1,00E-23	36%	WP_027142925.1	
**pMC2**	13	hypothetical protein	*Parasutterella excrementihominis*	70.9	70.9	50%	9,00E-13	45%	WP_040603707.1	putative phage repressor
**pMC2**	14	hypothetical protein	*Alcaligenes faecalis*	73.2	73.2	93%	1,00E-14	47%	WP_035267837.1	formate dehydrogenase subunit alpha
**pMC2**	15	No significant similarity found								
**pMC2**	16	hypothetical protein	*Delftia acidovorans*	173	173	89%	3,00E-51	65%	WP_016454567.1	transcriptional regulator CII
**pMC2**	17	hypothetical protein	*Acidovorax citrulli*	89.0	89.0	75%	1,00E-20	62%	WP_041827888.1	TetR family transcriptional regulator
**pMC2**	18	conjugal transfer	*Pseudomonas*	306	306	88%	4,00E-	31%	WP_041773113.1	

		protein TraC	*mendocina*				88			
**pMC2**	19	hypothetical protein	*Hylemonella gracilis*	92.8	92.8	75%	2,00E-21	57%	WP_035608219.1	putative Phage antitermination Q-like
**pMC2**	20	No significant similarity found								
**pMC2**	21	hypothetical protein	*Delftia acidovorans*	320	320	98%	3,00E-108	85%	EPD44780.1	phage terminase, small subunit
**pMC2**	22	hypothetical protein	*elftia acidovorans*	1033	1033	98%	0.0	85%	WP_016445316.1	terminase
**pMC2**	23	HK97 family phage portal protein	*Delftia* sp.	550	550	98%	0.0	65%	EZP51836.1	
**pMC2**	24	peptidase	*Delftia acidovorans*	362	362	99%	6,00E-124	80%	WP_034399341.1	
**pMC2**	25	HK97 family phage major capsid protein	*Comamonas testosteroni*	626	626	99%	0.0	85%	WP_034349600.1	
**pMC2**	26	hypothetical protein CTATCC11996_18657 Comamonas testosteroni	*Delftia* sp.	51.6	51.6	26%	9,00E-06	58%	EZP51833.1	
**pMC2**	27	No significant similarity found								
**pMC2**	28	phage head-tail adaptor	*Marinomonas phage*	43.9	43.9	92%	8,00E-04	38%	YP_006560248.1	
**pMC2**	29	bacteriophage TP901-1 ORF40-like protein	*Burkholderiales bacterium*	166	166	96%	5,00E-49	55%	BAP88849.1	
**pMC2**	30	No significant similarity found								
**pMC2**	31	hypothetical protein	*Ruegeria* sp.	99.4	99.4	81%	7,00E-23	45%	YP_613609.1	
**pMC2**	32	hypothetical protein	*Novosphingobium* sp.	43.9	43.9	86%	0.004	28%	WP_007678920.1	
**pMC2**	33	hypothetical protein	*Massilia* sp.	55.8	55.8	64%	9,00E-08	41%	WP_036176014.1	
**pMC2**	34	tail protein	*seudomonas aeruginosa*	148	148	17%	9,00E-33	40%	WP_034067959.1	
**pMC2**	35	carbohydrate-binding protein	*Acidovorax citrulli*	219	219	98%	3,00E-65	39%	WP_011794764.1	

**pMC2**	36	hypothetical protein	*Sulfurospirillum cavolei*	59.7	59.7	34%	6,00E-08	33%	WP_041963463.1	
**pMC2**	37	hypothetical protein	*elftia tsuruhatensis*	52.8	52.8	40%	2,00E-05	41%	KEH10790.1	
**pMC2**	38	hypothetical protein	*Alicycliphilus denitrificans*	55.1	55.1	69%	4,00E-07	43%	WP_013518562.1	
**pMC2**	39	hypothetical protein	*Acidovorax citrulli*	52.8	52.8	26%	1,00E-04	36%	ABM32217.1	
**pMC2**	40	No significant similarity found								
**pMC2**	41	hypothetical protein	*Massilia alkalitolerans*	71.6	71.6	45%	2,00E-12	58%	WP_027864255.1	
**pMC2**	42	hypothetical protein	*Herminiimonas* sp.	54.3	54.3	55%	1,00E-07	52%	WP_025915811.1	holin
**pMC2**	43	glycoside hydrolase	*Comamonas testosteroni*	205	205	99%	2,00E-64	69%	WP_043005020.1	
**pMC2**	44	hypothetical protein	*Alicycliphilus denitrificans*	87.8	87.8	96%	3,00E-20	50%	WP_013518568.1	
**pMC2**	45	hypothetical protein	*Delftia acidovorans*	147	147	98%	2,00E-41	63%	WP_034395290.1	
**pMC2**	46	hypothetical protein	*Nevskia ramosa*	157	157	52%	3,00E-39	53%	WP_033418158.1	neuroendocrine convertase 1
**pMC2**	47	hypothetical protein	*Alicycliphilus denitrificans*	117	117	85%	3,00E-31	77%	WP_013518572.1	
**pMC2**	48	No significant similarity found								
**pMC2**	49	No significant similarity found								
**pMC2**	50	integrase	*Comamonas testosteroni*	720	720	98%	0.0	69%	WP_034371762.1	
**pMC3**	1	No significant similarity found								
**pMC3**	2	hypothetical protein	*Bacillus cereus group*	68.2	68.2	98%	4,00E-13	100%	WP_000182548.1	
**pMC3**	3	No significant similarity found								
**pMC3**	4	No significant similarity found								

**pMC3**	5	hypothetical protein	*Bacillus cereus*	128	128	98%	1,00E-36	100%	WP_001038660.1	
**pMC3**	**6**	mobilization protein	*Bacillus cereus*	41.2	41.2	57%	0.004	83%	EPC06203.1	mobilization protein
**pMC3**	7	hypothetical protein	*Bacillus cereus*	976	976	99%	0.0	98%	WP_000901534.1	
**pMC3**	8	replication protein	*Bacillus cereus*	415	415	99%	5,00E-145	99%	WP_000170172.1	
**pMC3**	9	hypothetical protein	*Bacillus cereus*	58.5	58.5	69%	3,00E-09	62%	EDZ48787.1	
**pMC3**	10	hypothetical protein	*Bacillus cereus*	75.1	75.1	97%	4,00E-16	87%	WP_033719991.1	
**pMC3**	11	hypothetical protein	*Bacillus cereus*	109	109	98%	2,00E-29	100%	EEL58551.1	cold-shock DNA-binding domain-containing protein
**pMC3**	12	Cold shock protein	*Bacillus cereus*	134	134	98%	9,00E-39	100%	WP_000434813.1	
**pMC3**	13	hypothetical protein	*Bacillus cereus*	62.0	62.0	75%	4,00E-11	76%	WP_000903438.1	
**pMC3**	14	Barstar (barnase inhibitor) Paenibacillus curdlanolyticus YK9	*Paenibacillus curdlanolyticus*	187	187	98%	4,00E-57	53%	EFM10710.1	
**pMC3**	15	hypothetical protein	*Bacillus cereus*	186	186	98%	1,00E-58	97%	WP_002166780.1	
**pMC3**	16	hypothetical protein	*Bacillus cereus*	33.1	33.1	57%	1.4	61%	WP_000601186.1	
**pMC4**	1	hypothetical protein	*Acidovorax* sp.	57.4	57.4	81%	4,00E-09	71%	ABM40964.1	
**pMC4**	2	hypothetical protein	*Acidovorax* sp.	404	404	86%	2,00E-135	61%	ABM40963.1	zonular occludens toxin
**pMC4**	3	No significant similarity found								
**pMC4**	4	resolvase	*Comamonas testosteroni*	216	216	98%	2,00E-67	58%	WP_034378873.1	
**pMC4**	5	hypothetical protein	*Acidovorax ebreus*	54.3	54.3	54%	4,00E-07	49%	ACM33035.1	
**pMC4**	6	No significant similarity found								
**pMC4**	7	hypothetical protein	*Comamonas testosteroni*	42.4	42.4	49%	0.002	68%	WP_034357633.1	

**pMC4**	8	replication initiation factor	*Acidovorax citrulli*	322	322	86%	4,00E-104	56%	ABM32965.1	Replication initiation factor. Pfam02486
**pMC4**	9	hypothetical protein	*Ralstonia pickettii*	79.7	79.7	89%	1,00E-16	46%	WP_012435524.1	
**pMC4**	10	No significant similarity found								
**pMC4**	11	hypothetical protein	*Acidovorax* sp.	49.3	49.3	99%	2,00E-05	42%	WP_041835882.1	
**pMC4**	12	No significant similarity found								
**pMC5**	1	hypothetical protein	*Streptococcus parasanguinis*	724	724	99%	0.0	99%	ETD10351.1	cell division protein FtsK
**pMC5**	2	hypothetical protein	*Streptococcus parasanguinis*	229	229	99%	9,00E-75	99%	WP_023920331.1	
**pMC5**	3	Putative ribonucleotide transport ATP-binding protein mkl	*Streptococcus parasanguinis*	145	145	98%	4,00E-43	92%	WP_023920329.1	
**pMC5**	4	hypothetical protein	*Streptococcus agalactiae*	166	166	88%	6,00E-50	75%	WP_000711035.1	
**pMC5**	5	replication initiation factor	*Streptococcus parasanguinis*	730	730	99%	0.0	97%	WP_023920333.1	replication initiation factor. Pfam02486
**pMC5**	6	No significant similarity found								
**pMC6**	1	Relaxase	*Escherichia coli*	2439	2439	99%	0.0	99%	AEB22177.1	
**pMC6**	2	hypothetical protein	*Salmonella enterica*	258	258	99%	1,00E-85	100%	WP_001157192.1	relaxosome protein
**pMC6**	3	hypothetical protein	Escherichia coli	284	284	100%	1e-95	99%	WP_021545896.1	
**pMC6**	4	conjugal transfer protein TraL	*Salmonella enterica*	550	550	99%	0.0	100%	WP_001051252.1	relaxosome protein
**pMC6**	5	hypothetical protein	*Enterobacteriaceae*	319	319	84%	4,00E-108	100%	WP_006882065.1	
**pMC6**	6	hypothetical protein	*Enterobacteriaceae*	238	238	99%	2,00E-78	100%	WP_006882064.1	
**pMC6**	7	hypothetical protein	*Escherichia coli*	152	152	98%	4,00E-45	97%	WP_021545900.1	
**pMC6**	8	hypothetical protein	*Enterobacteriaceae*	468	468	99%	3,00E-165	99%	WP_006882056.1	

**pMC6**	9	hypothetical protein	*Enterobacteriaceae*	580	580	99%	0.0	99%	WP_000246370.1	ParB-like nuclease
**pMC6**	10	hypothetical protein	*Enterobacteriaceae*	226	226	99%	6,00E-74	100%	WP_006882053.1	
**pMC6**	11	hypothetical protein	*Escherichia coli*	105	105	98%	3,00E-28	100%	AEB22188.1	
**pMC6**	12	hypothetical protein	*Enterobacteriaceae*	134	134	98%	2,00E-38	100%	WP_006882049.1	
**pMC6**	13	hypothetical protein	*Enterobacteriaceae*	400	400	99%	7,00E-140	100%	WP_006882046.1	DNA packaging protein FI
**pMC6**	14	putative conjugal transfer protein traA	*Salmonella enterica*	652	652	99%	0.0	100%	WP_000421118.1	VirB6
**pMC6**	15	hypothetical protein	*Enterobacteriaceae*	167	167	98%	1,00E-51	100%	WP_000570802.1	toxin-antitoxin system
**pMC6**	16	hypothetical protein	*Salmonella enterica*	63.9	63.9	71%	1,00E-11	63%	WP_024139611.1	
**pMC6**	17	plasmid replication protein	*Enterobacteriaceae*	658	658	90%	0.0	99%	WP_006882108.1	Initiator Replication protein. Pfam0105
**pMC6**	18	transcriptional regulator	*Enterobacteriaceae*	339	339	99%	2,00E-116	99%	WP_000288043.1	
**pMC6**	19	hypothetical protein	*Enterobacteriaceae*	249	249	89%	3,00E-82	100%	WP_000902259.1	
**pMC6**	20	conjugal transfer protein TraB	*Enterobacteriaceae*	465	465	99%	2,00E-164	100%	WP_000241899.1	VirB1
**pMC6**	21	conjugal transfer protein TraC	*Enterobacteriaceae*	167	167	99%	8,00E-51	100%	WP_006882103.1	VirB2
**pMC6**	22	conjugal transfer protein TraD	*Enterobacteriaceae*	239	239	99%	3,00E-78	100%	WP_000796685.1	VirB3
**pMC6**	23	conjugal transfer protein TraE	*Salmonella enterica*	1678	1678	97%	0.0	99%	WP_000106190.1	VirB4
**pMC6**	24	Putative conjugal transfer protein TraF	*Escherichia coli*	426	426	99%	8,00E-148	100%	AEB22148.1	VirB5
**pMC6**	25	Putative conjugal transfer protein TraF	*Enterobacteriaceae*	116	116	98%	4,00E-32	100%	WP_001128573.1	VirB7
**pMC6**	26	conjugal transfer protein traG	*Enterobacteriaceae*	486	486	99%	1,00E-171	100%	WP_000476773.1	VirB8
**pMC6**	27	conjugal transfer protein traH	*Enterobacteriaceae*	542	542	99%	0.0	100%	WP_000722128.1	VirB9

**pMC6**	28	conjugal transfer protein traI	*Salmonella enterica*	643	643	99%	0.0	99%	WP_000999413.1	VirB10
**pMC6**	29	secretion system protein E	*Enterobacteriaceae*	736	736	99%	0.0	100%	WP_001005926.1	VirB11
**pMC6**	30	conjugal transfer protein traK	*Salmonella enterica*	1269	1269	99%	0.0	100%	WP_001177100.1	VirD4 (coupling protein, T4CP)
**pMC6**	31	arginine transportr	*Enterobacteriaceae*	204	204	99%	1,00E-64	100%	WP_000848516.1	
**pMC6**	32	hypothetical protein	*Enterobacteriaceae*	274	274	99%	8,00E-92	100%	WP_000368397.1	
**pMC6**	33	DNA topoisomerase III	*Salmonella enterica*	1448	1448	99%	0.0	99%	WP_000604074.1	
**pMC6**	34	DNA-binding protein	*Enterobacteriaceae*	217	217	99%	3,00E-69	100%	WP_000004210.1	H-NS histone family
**pMC6**	35	hypothetical protein	*Salmonella enterica*	226	226	99%	8,00E-74	99%	WP_000083087.1	
**pMC6**	36	cysteinyl-tRNA synthetase	*Pseudomonas syringae*	155	155	85%	2,00E-44	95%	WP_032698189.1	cysteinyl-tRNA synthetase
**pMC6**	37	cysteinyl-tRNA synthetase	*Pseudomonas resinovorans*	283	283	99%	8,00E-91	97%	WP_041770341.1	cysteinyl-tRNA synthetase
**pMC6**	38	hypothetical protein	*Pseudomonas aeruginosa*	132	132	97%	6,00E-38	89%	WP_033957408.1	cysteinyl-tRNA synthetase
**pMC6**	39	hypothetical protein	*Pseudomonas alcaligenes*	160	160	42%	2,00E-45	67%	WP_021220100.1	GCN5-related N-acetyltransferase
**pMC6**	40	thiol:disulfide interchange protein	*Pseudomonas oleovorans*	185	185	94%	2,00E-52	80%	WP_037051403.1	
**pMC6**	41	thiol:disulfide interchange protein	*Pseudomonas oleovorans*	520	520	89%	2,00E-174	78%	WP_037051403.1	
**pMC6**	42	histidinol phosphatase	*Pseudomonas oleovorans*	126	162	94%	6,00E-33	84%	WP_037051219.1	
**pMC6**	43	GntR family transcriptional regulator	*Pseudomonas oleovorans*	610	610	66%	0.0	80%	WP_037051217.1	
**pMC6**	44	histidinol phosphatase	*Pseudomonas oleovorans*	397	397	98%	6,00E-135	77%	WP_037051219.1	

Table S5. List of antibiotic resistance genes identified in the plasmid sequence dataset.

GenDB ID	Description (best BLASTp hit at NCBI)	Antibiotic resistance class	Gene name	Organism	Max score	Total score	Query cover	E value	Max ident	Accession number
AP_369	family drug resistance transporter	multidrug	*emrD*	*Pseudomonas fluorescens*	708	708	99%	0.0	83%	YP_005208868.1
BJP_10004	Multidrug resistance efflux pump	multidrug	*mexH*	*Stenotrophomonas maltophilia*	228	228	93%	2,00E-70	74%	WP_005415800.1
BJP_10347	antibiotic ABC transporter permease	multidrug		*Serratia marcescens*	103	103	94%	4,00E-25	100%	WP_016929317.1
BJP_10349	antibiotic ABC transporter permease	multidrug		*Serratia marcescens*	404	514	98%	7,00E-138	100%	WP_016929317.1
BJP_10488	multidrug transporter	multidrug	*opmD*	*Variovorax paradoxus*	155	155	92%	2,00E-40	44%	WP_020723353.1
BJP_10538	beta-lactamase/D-alanine carboxypeptidase	β-lactams	*bl1_ampC*	*Escherichia coli*	622	622	99%	0.0	100%	NP_418574.1
BJP_10562	Multidrug translocase MdfA, partial	multidrug	*mdfA*	*Escherichia coli*	320	320	99%	7,00E-109	100%	WP_001319791.1
BJP_10749	streptomycin 3''-kinase	aminoglycosides	*aph33ib*	*Xanthomonas campestris*	548	548	98%	0.0	100%	YP_364056.1
BJP_10828	multidrug resistance protein MdtH	multidrug	*mdtH*	*Serratia marcescens*	102	102	98%	2,00E-24	100%	WP_004929633.1
BJP_1085	bleomycin resistance protein	bleomycin		*Enterobacter asburiae*	163	163	99%	2E-48	65%	YP_004827035.1
BJP_10930	multidrug transporter	multidrug		*Escherichia coli*	246	246	91%	2,00E-81	100%	WP_001442852.1
BJP_10953	streptomycin 3''-kinase	aminoglycosides	*aph33ib*	*Xanthomonas campestris*	548	548	98%	0.0	100%	YP_364056.1

BJP_10956	tetracycline resistance protein TetA	tetracyclines	*tetA*	*Paenibacillus elgii*	209	209	96%	4,00E-61	47%	WP_010492684.1
BJP_10957	glyoxalase/bleomycin resistance protein/dioxygenase	bleomycin		*Serratia marcescens*	256	256	99%	1,00E-85	100%	YP_007406930.1
BJP_10964	macrolide export ATP-binding/permease MacB	macrolides	*macB*	*Delftia acidovorans*	522	522	99%	1,00E-178	84%	WP_016452930.1
BJP_1449	glyoxalase/bleomycin resistance protein/dioxygenase family protein	bleomycin		*Pseudomonas fluorescens*	648	648	99%	0.0	97%	YP_006326734.1
BJP_1470	tetracycline resistance protein, class G (TETA(G))	tetracyclines	*tetA(G)*	*Acinetobacter baumannii*	667	667	99%	0.0	99%	YP_001715369.1
BJP_1472	tetracycline repressor protein class G	tetracyclines	*tetA(G)*	*Acinetobacter baumannii*	400	400	99%	9,00E-140	100%	YP_001715370.1
BJP_1473	chloramphenicol and florfenicol resistance protein (CmlA)	phenicols	*cmlA9*	*Acinetobacter baumannii*	755	755	99%	0.0	100%	YP_001715371.1
BJP_1477	TetR family transcriptional regulator	tetracyclines	*tetR*	*Leucobacter chromiiresistens*	384	384	99%	1,00E-133	100%	WP_010154754.1
BJP_1479	tetracycline resistance protein	tetracyclines	*tet33*	*Leucobacter chromiiresistens*	728	728	99%	0.0	99%	WP_010154755.1
BJP_1564	aminoglycoside phosphotransferase	aminoglycosides		*Kribbella flavida*	181	181	67%	4,00E-51	51%	YP_003379039.1
BJP_1577	multidrug transporter	multidrug		*Ochrobactrum anthropi*	509	509	99%	2,00E-178	98%	WP_010660379.1
BJP_1578	MFS transporter	multidrug		*Ochrobactrum anthropi*	924	924	99%	0.0	94%	WP_010660378.1
BJP_1773	beta-lactamase	β-lactams	*bl3_cphA*	*Serratia fonticol*	382	382	90%	3,00E-	77%	AAF09244.1

BJP_1786	class B beta-lactamase	β-lactams	*bl3_l*	*uncultured bacterium*	166	166	98%	131 6,00E-48	62%	AGD93225.1
BJP_1946	multidrug transporter	multidrug		*Hafnia alvei*	296	296	98%	2,00E-92	52%	WP_004092579.1
BJP_1947	Putative Na+-driven multidrug efflux pump, partial	multidrug		*Moritella sp.*	75.5	75.5	98%	3,00E-16	57%	WP_006031386.1
BJP_2046	multidrug efflux system protein	multidrug	*mdtL*	*Escherichia coli*	723	723	97%	0.0	100%	NP_418166.1
BJP_2274	aminoglycoside 3'-phosphotransferase	aminoglycosides	*aph3ib*	*uncultured bacterium*	296	296	99%	5,00E-99	100%	YP_112402.1
BJP_2281	multidrug transporter MatE	multidrug	*matE*	*Variovorax paradoxus*	452	452	90%	2,00E-152	67%	WP_018906131.1
BJP_2342	efflux protein	multidrug		*Burkholderia gladioli*	241	241	89%	5,00E-72	54%	YP_004362548.1
BJP_2845	multidrug resistance efflux transporter conferring overexpression resistance to norfloxacin and enoxacin	multidrug	*mdtH*	*Escherichia coli*	685	685	97%	0.0	100%	NP_415583.4
BJP_2917	multidrug resistance outer membrane protein MdtQ	multidrug	*mdtQ*	*Escherichia coli*	592	592	99%	0.0	100%	YP_001731081.1
BJP_3020	multidrug transporter	multidrug	*mdtF*	*Escherichia coli*	434	434	99%	6,00E-142	99%	WP_000024891.1
BJP_3282	multiple antibiotic resistance protein MarC	multidrug	*marC*	*Escherichia coli*	438	438	99%	4,00E-154	99%	WP_001527063.1

BJP_3283	DNA-binding transcriptional repressor of multiple antibiotic resistance	multidrug		*Escherichia coli*	291	291	99%	7,00E-99	99%	NP_416047.4
BJP_3284	Multiple antibiotic resistance protein marA	multidrug	*marA*	*Shigella flexneri*	267	267	99%	1,00E-89	100%	YP_005727252.1
BJP_3285	multiple antibiotic resistance protein MarB	multidrug	*marB*	*Escherichia coli*	117	117	98%	1,00E-32	96%	YP_006133711.1
BJP_3342	multidrug RND transporter, membrane fusion protein MexP	multidrug	*mexE*	*Pseudomonas fluorescens*	493	493	98%	3,00E-171	83%	YP_006324484.1
BJP_3394	antibiotic transport system permease	multidrug		*Delftia acidovorans*	132	132	99%	5,00E-33	70%	WP_016445623.1
BJP_3395	antibiotic transport system permease	multidrug		*Delftia acidovorans*	500	500	93%	3,00E-173	82%	WP_016453267.1
BJP_3397	beta-lactamase	β-lactams	*bl2c_pse1*	*Acidovorax* sp.	101	101	98%	1,00E-23	48%	WP_007855123.1
BJP_3434	antibiotic transport system permease	multidrug		*Delftia acidovorans*	224	224	96%	3,00E-69	82%	WP_016453268.1
BJP_3453	metallo-beta-lactamase	β-lactams	*bl3_cphA*	*Acidovorax* sp.	500	500	97%	6,00E-175	75%	GAD21457.1
BJP_3557	Multidrug resistance protein, partial [Escherichia coli	multidrug		*Escherichia coli*	308	308	99%	3,00E-105	99%	YP_006139558.1
BJP_3558	multidrug resistance protein Y [Escherichia coli	multidrug		*Escherichia coli*	907	907	99%	0.0	99%	WP_001613551.1
BJP_3829	beta-lactamase-like	β-lactams	*bl3_l*	*Polaromonas* sp.	406	406	96%	2,00E-137	71%	YP_552021.1
BJP_4008	beta-lactamase	β-lactams	*mdtK*	*Burkholderia* sp.	637	637	92%	0.0	58%	WP_008345909.1

BJP_4048	hydrophobe/amphiphile efflux-1 (HAE1) family transporter	multidrug		*Delftia* sp.	1455	1455	99%	0.0	84%	YP_004488748.1
BJP_4120	tetracycline resistance regulatory protein TetR	tetracyclines	*tetR*	*Aeromonas salmonicida*	213	213	96%	2,00E-67	100%	NP_387462.1
BJP_4122	tetracycline resistance protein, partial	tetracyclines	*tetC*	*Pasteurella dagmatis*	249	249	83%	5,00E-81	100%	WP_005765649.1
BJP_4123	tetracycline resistance protein, class C, partial	tetracyclines	*tetC*	*Escherichia* sp.	428	428	99%	2,00E-150	100%	WP_002431094.1
BJP_4183	multidrug transporter	multidrug		*Escherichia coli*	160	160	99%	4,00E-48	99%	WP_001410377.1
BJP_4744	glyoxalase/bleomycin resistance protein/dioxygenase	bleomycin		*Serratia proteamaculans*	199	199	93%	2,00E-61	56%	YP_001478533.1
BJP_4808	beta-lactamase	β-lactams	*bl1_pao*	*Pandoraea* sp.	403	403	98%	8,00E-136	61%	WP_010803889.1
BJP_4809	glutathione transferase	fosmidomycin	*fosA*	*Comamonas testosteroni*	190	190	99%	5,00E-59	67%	WP_003068211.1
BJP_4998	beta-lactamase	β-lactams	*bl1_ampC*	*Streptomyces* sp.	210	210	86%	4,00E-60	41%	WP_008743065.1
BJP_5116	outer membrane factor of efflux pump	multidrug	*mdtP*	*Serratia marcescens*	791	791	99%	0.0	99%	YP_007407670.1
BJP_5279	acriflavine resistance protein E	acriflavine	*acrA*	*Escherichia coli*	707	707	99%	0.0	99%	WP_001626061.1
BJP_5281	multidrug efflux system protein, partial	multidrug	*acrB*	*Escherichia coli*	89.7	89.7	93%	8,00E-22	100%	WP_001320883.1
BJP_5319	bleomycin resistance protein	bleomycin		*Pusillimonas noertemannii*	394	394	99%	8,00E-135	67%	WP_017522678.1

BJP_5515	macrolide transporter subunit MacA, partial	macrolides	*macA*	*Escherichia coli*	189	189	99%	5,00E-58	99%	WP_000746444.1
BJP_5614	Bcr/CflA subfamily drug resistance transporter	multidrug		*Cupriavidus necator*	139	139	84%	3,00E-37	74%	WP_006576966.1
BJP_5700	multidrug transporter	multidrug		*Planctomyces maris*	211	211	97%	9,00E-62	54%	WP_002644159.1
BJP_6026	glyoxalase/bleomycin resistance protein/dioxygenase superfamily protein	bleomycin		*Burkholderia cenocepacia*	320	320	97%	8,00E-107	81%	YP_002230299.1
BJP_6093	fosmidomycin resistance protein	fosmidomycin	*rosA*	*Enterobacter aerogenes*	434	434	97%	1,00E-146	73%	YP_004592799.1
BJP_6101	glyoxalase/bleomycin resistance protein/dioxygenase	bleomycin		*Pseudomonas* sp.	182	182	97%	2,00E-53	56%	WP_009619919.1
BJP_6173	beta-lactamase	β-lactams	*bl1_asbA*	*Serratia marcescens*	363	363	98%	2,00E-122	99%	WP_016927773.1
BJP_6253	beta-lactamase domain-containing protein	β-lactams	*bl3_cphA*	*Polaromonas naphthalenivorans*	413	413	98%	2,00E-141	70%	YP_980965.1
BJP_6622	multidrug resistance protein MdtO	multidrug	*mdtO*	*Escherichia coli*	258	258	99%	1,00E-85	100%	WP_001544272.1
BJP_6623	efflux transporter, RND family, MFP subunit	multidrug	*mdtN*	*Escherichia coli*	551	551	99%	0.0	99%	WP_001638638.1
BJP_6895	acriflavin resistance protein	acriflavine	*mexF*	*Variovorax paradoxus*	508	508	98%	6,00E-165	64%	YP_002945489.1
BJP_7098	chloramphenicol resistance protein	phenicols	*cmlA6*	*Klebsiella pneumoniae*	701	701	99%	0.0	99%	YP_001338812.1
BJP_7385	TetR family transcriptional regulator	tetracyclines	*tetR*	*Paenibacillus polymyxa*	265	265	97%	8,00E-87	64%	YP_003948162.1

BJP_7397	beta-lactamase:copper amine oxidase-like protein	β-lactams	*bl1_asbA*	*Acetivibrio cellulolyticus*	233	233	93%	3,00E-65	32%	WP_010248813.1
BJP_749	penicillin-binding protein	β-lactams	*bl1_och*	*Ochrobactrum intermedium*	573	573	99%	0.0	92%	WP_006470559.1
BJP_7501	beta-lactamase	β-lactams		*Cupriavidus taiwanensis*	217	217	99%	2,00E-67	71%	WP_018004145.1
BJP_7527	chloramphenicol acetyltransferase	phenicols	*catA16*	*Streptococcus pneumoniae*	183	183	97%	2,00E-55	61%	CAO82974.1
BJP_7974	multidrug transporter	multidrug		*Escherichia coli*	160	160	99%	4,00E-48	99%	WP_001410377.1
BJP_7982	multidrug ABC transporter ATP-binding protein, partial	multidrug		*Escherichia coli*	218	218	97%	6,00E-69	98%	WP_020240856.1
BJP_8071	glyoxalase/bleomycin resistance protein/dioxygenase	bleomycin		*Delftia acidovorans*	304	304	98%	2,00E-102	76%	YP_001561072.1
BJP_8089	multidrug transporter	multidrug	*emre*	*Serratia marcescens*	139	139	99%	4,00E-40	100%	WP_016927446.1
BJP_8090	small multidrug resistance protein	multidrug	*emrE*	*Serratia proteamaculans*	222	222	98%	3,00E-72	91%	YP_001478995.1
BJP_8121	Macrolide export ATP-binding/permease protein MacB, partial	macrolides	*macB*	*Escherichia coli*	744	744	99%	0.0	100%	WP_000316535.1
BJP_8267	glyoxalase	bleomycin		*Serratia marcescens*	314	314	99%	8,00E-107	100%	WP_016928790.1
BJP_8309	multidrug transporter	multidrug	*mdtM*	*Escherichia coli*	517	517	99%	0.0	99%	WP_020236418.1
BJP_8388	beta-lactamase	β-lactams		*Spirosoma spitsbergense*	227	227	77%	5,00E-65	40%	WP_020607745.1
BJP_8406	membrane protein	macrolides	*macA*	*Escherichia coli*	372	372	99%	4,00E-	99%	WP_001384391.1

								128		
BJP_8407	ABC transporter ATP-binding protein	macrolides	*macB*	*Escherichia coli*	424	424	98%	1,00E-146	99%	WP_000188121.1
BJP_8592	acriflavin resistance protein A, partial	acriflavine	*acrA*	*Escherichia coli*	483	483	99%	6,00E-170	100%	YP_668452.1
BJP_8593	multidrug efflux system protein AcrB	multidrug	*acrB*	*Salmonella enterica*	83.2	83.2	96%	3,00E-19	95%	WP_001717341.1
BJP_8654	fosmidomycin resistance protein	fosmidomycin	*rosA*	*Shigella sonnei*	417	417	98%	8,00E-143	100%	YP_309479.1
BJP_8685	multidrug resistance protein MdtN	multidrug	*mdtN*	*Serratia marcescens*	146	146	97%	3,00E-40	98%	WP_004941492.1
BJP_8992	TetA(41)	tetracyclines	*tetA(41)*	*Serratia marcescens*	396	396	92%	8,00E-134	97%	AAP93922.1
BJP_8993	TetR family transcriptional regulator	tetracyclines	*tetR*	*Serratia marcescens*	187	187	96%	8,00E-58	96%	YP_007406306.1
BJP_9086	bicyclomycin/multidrug efflux system	bacitracin	*bcr*	*Serratia marcescens*	524	524	99%	0.0	100%	YP_007407161.1
BJP_9089	macrolide export ATP-binding/permease MacB	macrolides	*macB*	*Delftia acidovorans*	183	183	75%	3,00E-51	82%	WP_016452930.1
BJP_9091	macrolide-specific efflux protein MacA	macrolides	*macA*	*Delftia acidovorans*	318	318	97%	8,00E-104	72%	WP_016450742.1
BJP_9128	glyoxalase	bleomycin		*Serratia marcescens*	646	646	99%	0.0	99%	WP_016930047.1
BJP_9174	aminoglycoside resistance protein	aminoglycosides	*aac6ib*	*Alcanivorax pacificus*	279	279	98%	4,00E-90	56%	WP_008736168.1
BJP_9307	ABC transporter ATP-binding protein	macrolides	*carA*	*Serratia marcescens*	471	471	99%	1E-166	99%	WP_016928844.1
BJP_9313	TetR family transcriptional regulator	tetracyclines		*Serratia proteamaculans*	329	329	90%	9,00E-112	84%	YP_001479369.1

BJP_9390	Fosmidomycin resistance protein	fosmidomycin	*rosA*	*Serratia marcescens*	121	121	98%	8,00E-31	99%	WP_016928828.1
BJP_9514	multidrug efflux system protein MdtO	multidrug	*mdtO*	*Serratia marcescens*	614	614	99%	0.0	99%	WP_004941490.1
BJP_9677	metal-dependent hydrolases of the beta-lactamase superfamily I	β-lactams		*uncultured bacterium*	308	308	99%	8,00E-102	62%	AGC71534.1
BJP_9755	glyoxalase/bleomycin resistance protein/dioxygenase	bleomycin		*Cupriavidus metallidurans*	75.1	75.1	91%	1,00E-14	48%	YP_585917.1
BJP_9756	glyoxalase/bleomycin resistance protein/dioxygenase	bleomycin		*Cupriavidus* sp.	251	251	90%	4,00E-80	70%	WP_008651425.1
BJP_9759	Bcr/CflA subfamily drug resistance transporter	multidrug	*emrD*	*Cupriavidus necator*	207	207	91%	8,00E-61	59%	WP_006576966.1
BJP_9772	hydrophobe/amphiphile efflux-1 (HAE1) family transporter	multidrug	*acrB*	*Delftia* sp.	167	167	98%	5,00E-46	94%	YP_004488748.1
BJP_9824	bleomycin resistance protein	bleomycin		*Serratia plymuthica*	149	149	95%	2,00E-44	91%	WP_006319297.1
BJP_9888	bleomycin resistance protein	bleomycin		*Serratia plymuthica*	149	149	95%	2,00E-44	91%	WP_006319297.1

Table S6. List of metal resistance genes present in the plasmid sequence dataset.

GenDB ID	Description (best BLASTp hit at NCBI)	Organism	Max score	Total score	Query cover	E value	Max ident	Accession number	Metal(s) resistance
BJP_756	zinc efflux system	*Mesorhizobium* sp.	469	469	99%	1,00E-163	82%	WP_006329619.1	zinc
BJP_774	RND family efflux transporter MFP subunit	*Acidovorax* sp.	238	238	64%	2,00E-71	77%	YP_986110.1	cobalt, zinc
BJP_775	outer membrane efflux protein	*Acidovorax* sp.	492	492	97%	3,00E-168	73%	YP_986109.1	cobalt, zinc
BJP_776	cobalt-zinc-cadmium resistance protein	*Cupriavidus metallidurans*	74.7	74.7	98%	1,00E-14	42%	YP_145592.1	cobalt, zinc, carmium
BJP_791	Cd(II)/Pb(II)-responsive transcriptional regulator	*Burkholderiales bacterium*	197	197	99%	2,00E-61	64%	WP_009552846.1	carmium, lead
BJP_1604	tellurite, selenium methyltransferase, SAM-dependent; tellurite, selenium resistance protein	*Escherichia coli*	409	409	99%	1,00E-143	100%	NP_415947.1	tellurite, selenium
BJP_1605	tellurite resistance protein TehA	*Escherichia coli*	620	620	99%	0.0	100%	WP_000222935.1	tellurite
BJP_1810	MerR protein	*Pseudomonas* sp.	296	296	99%	2,00E-100	100%	NP_862494.1	mercury
BJP_1811	MerF protein	*Pseudomonas* sp.	207	207	99%	2,00E-66	99%	NP_862493.1	mercury
BJP_1812	MerP protein	*Pseudomonas* sp.	133	133	71%	3,00E-38	100%	NP_862492.1	mercury
BJP_1813	meruric reductase	*Plasmid pMCBF1*	179	179	98%	3,00E-51	100%	AAY97939.1	mercury

BJP_1814	mercuric reductase	*Klebsiella pneumoniae*	1038	1038	99%	0.0	99%	EQA20972.1	mercury
BJP_1815	MerD protein	*Pseudomonas* sp.	182	182	99%	2,00E-56	100%	NP_862489.1	mercury
BJP_1816	MerE protein	*Pseudomonas* sp.	157	157	98%	3,00E-48	100%	NP_862488.1	mercury
BJP_2103	MerR	*Pseudomonas alcaligenes*	251	251	99%	2,00E-83	100%	YP_025338.1	mercury
BJP_2104	mercuric transport protein	*Pseudomonas stutzeri*	92.4	92.4	84%	2,00E-21	100%	YP_001173902.1	mercury
BJP_2105	Mercuric transport protein periplasmic component precursor	*Thiomonas* sp.	125	125	98%	5,00E-35	85%	YP_003622579.1	mercury
BJP_2192	MerR family transcriptional regulator	*Idiomarina loihiensis*	277	277	99%	3,00E-93	99%	YP_155021.1	mercury
BJP_2193	CDF family heavy metal/H(+) antiporter	*Burkholderia xenovorans*	561	561	99%	0.0	99%	YP_552434.1	heavy metal
BJP_3085	arsenic transporter	*Ochrobactrum anthropi*	109	109	94%	3,00E-27	98%	WP_010660310.1	arsenic
BJP_3083	ArsR family transcriptional regulator	*Ochrobactrum anthropi*	145	145	98%	4,00E-42	94%	WP_010660309.1	arsenic
BJP_3082	ArsR family transcriptional regulator	*Ochrobactrum anthropi*	194	194	91%	6,00E-61	97%	WP_010660309.1	arsenic
BJP_3081	ArsR family transcriptional regulator	*Ochrobactrum* sp.	224	224	99%	2,00E-73	92%	WP_007881800.1	arsenic
BJP_3505	cation diffusion facilitator family transporter	*Delftia acidovorans*	441	441	99%	7,00E-152	75%	YP_001561495.1	cobalt, zinc, carmium

BJP_4019	C4-dicarboxylate transporter/malic acid transport protein	*Leptothrix cholodnii*	226	226	85%	5,00E-67	52%	YP_001792807.1	tellurite
BJP_4103	RND transporter	*Acidovorax* sp.	322	322	85%	2,00E-103	60%	WP_008903848.1	
BJP_4404	arsenical resistance protein ArsH	*Afipia clevelandensis*	358	358	96%	4,00E-122	75%	WP_002713267.1	arsenic
BJP_4405	ArsC2	*Ochrobactrum tritici*	262	262	99%	2,00E-87	94%	ABF48395.1	arsenic
BJP_4406	arsenic transporter	*Ochrobactrum anthropi*	172	172	99%	5,00E-50	99%	WP_010660310.1	arsenic
BJP_4486	MerR family transcriptional regulator	*Pseudomonas mandelii*	216	216	99%	7,00E-70	91%	WP_019582817.1	mercury
BJP_5212	zinc/iron permease	*Polaromonas naphthalenivorans*	213	213	88%	1,00E-63	69%	YP_981719.1	zinc, iron
BJP_5275	cobalt-zinc-cadmium resistance protein CzcC	*Delftia acidovorans*	121	121	73%	2,00E-29	54%	WP_016448635.1	cobalt, zinc, carmium
BJP_5274	two component heavy metal response transcriptional regulator	*Delftia acidovorans*	358	358	98%	2,00E-122	77%	YP_001562121.1	heavy metal
BJP_5551	arsenite transporter	*Escherichia coli*	347	347	99%	3,00E-119	99%	YP_006129959.1	arsenic
BJP_5552	Putative arsenate reductase-like protein	*Escherichia coli*	105	105	48%	1,00E-27	100%	P0CF86.1	arsenic
BJP_5636	nickel transporter subunit	*Escherichia coli*	543	543	99%	0.0	100%	NP_417937.1	nickel
BJP_5637	nickel ABC transporter ATP-binding protein	*Escherichia coli*	318	318	99%	2,00E-107	100%	WP_001187280.1	nickel
BJP_5726	nickel/cobalt efflux system rcnA	*Escherichia coli*	479	479	99%	9,00E-169	99%	WP_001685159.1	nickel, cobalt

BJP_5737	heavy metal translocating P-type ATPase	*Polaromonas naphthalenivorans*	170	170	74%	5,00E-46	65%	YP_984001.1	heavy metal
BJP_5741	MerR family transcriptional regulator	*Ramlibacter tataouinensis*	201	201	98%	4,00E-63	69%	YP_004618893.1	mercury
BJP_5772	C4-dicarboxylate ABC transporter	*Pseudomonas* sp.	448	448	99%	2,00E-154	89%	WP_008059900.1	tellurite
BJP_5874	heavy metal efflux pump, CzcA family protein	*Escherichia coli*	593	593	99%	0.0	99%	WP_001740261.1	heavy metal
BJP_7145	ArsR family transcriptional regulator	*Ochrobactrum* sp.	213	213	99%	1,00E-68	88%	WP_007881800.1	arsenic
BJP_7147	arsenic transporter	*Ochrobactrum anthropi*	109	109	94%	4,00E-27	96%	WP_010660310.1	arsenic
BJP_7474	heavy metal efflux protein	*Acidovorax* sp.	213	213	91%	2,00E-65	81%	ABE73718.1	heavy metal
BJP_7559	ArsR family transcriptional regulator	*Limnohabitans* sp.	148	148	72%	5,00E-43	71%	WP_019429746.1	arsenic
BJP_7932	nickel transporter ATP-binding protein NikD, partial	*Escherichia coli*	161	161	97%	6,00E-49	100%	WP_001319468.1	nickel
BJP_7931	nickel ABC transporter permease	*Escherichia coli*	488	488	99%	7,00E-172	99%	WP_001008967.1	nickel
BJP_7930	nickel transporter permease NikB	*Shigella flexneri*	169	169	98%	2,00E-49	100%	YP_690824.1	nickel
BJP_8188	mercuric transport periplasmic protein	*Acidobacterium capsulatum*	96.3	96.3	98%	1,00E-23	55%	YP_002754327.1	mercury
BJP_8187	mercury transporter MerT	*Oceanibaculum indicum*	56.6	56.6	89%	5,00E-08	34%	WP_008946214.1	mercury
BJP_8384	putative transcriptional regulator MerR	*uncultured bacterium*	183	183	98%	6,00E-57	100%	NP_858038.1	mercury

BJP_8597	outer membrane efflux protein	*Alicycliphilus denitrificans*	712	712	99%	0.0	82%	YP_004388576.1	copper
BJP_8917	zinc import ATP-binding protein ZnuC	*Escherichia coli*	307	307	99%	3,00E-103	100%	WP_001571550.1	zinc
BJP_8954	merR family transcriptional regulator	*Pseudomonas* sp.	112	112	98%	8,00E-30	89%	YP_007030358.1	mercury
BJP_8992	merR family transcriptional regulator	*Pseudomonas* sp.	112	112	98%	8,00E-30	89%	YP_007030358.1	mercury
BJP_10150	iron transporter FeoB	*Acidovorax* sp.	239	239	84%	3,00E-72	76%	WP_020227043.1	iron
AP_187	zinc ABC transporter	*Paracoccus* sp.	589	589	94%	0.0	94%	WP_010398614.1	zinc
AP_227	ArsR	*Sinorhizobium* sp.	211	211	86%	7,00E-68	99%	YP_007974287.1	arsenic
AP_229	ArsC	*Sinorhizobium* sp.	363	363	99%	4,00E-126	100%	YP_007974288.1	arsenic
AP_230	ArsC	*Sinorhizobium* sp.	293	293	99%	3,00E-99	100%	YP_007974289.1	arsenic
AP_231	ArsB	*Sinorhizobium* sp.	593	593	99%	0.0	100%	YP_007974290.1	arsenic
AP_234	ArsH	*Sinorhizobium* sp.	457	457	99%	2,00E-161	100%	YP_007974293.1	arsenic
AP_235	ArsR	*Sinorhizobium* sp.	190	190	99%	6,00E-60	100%	YP_007974294.1	arsenic
AP_601	MerR transcriptional regular	*uncultured bacterium*	279	279	99%	6,00E-94	92%	ADD63292.1	mercury
AP_602	mercuric transport protein	*Shigella flexneri*	176	176	99%	4,00E-54	94%	NP_052882.1	mercury
AP_603	mercury uptake protein	*Delftia acidovorans*	128	128	71%	3,00E-36	98%	AAP88280.1	mercury
AP_604	mercury transporter MerC	*Ralstonia* sp.	227	227	98%	1,00E-	96%	WP_004636756.1	mercury

						73			
AP_605	mercuric reductase	*uncultured bacterium*	1023	1023	99%	0.0	97%	AFN66362.1	mercury
BJP_676	ArsR family transcriptional regulator	*Mesorhizobium alhagi*	169	169	92%	1,00E-51	73%	WP_008834809.1	arsenic
BJP_4060	ferric uptake regulator family protein	*Escherichia coli*	299	299	99%	2,00E-101	99%	WP_001708323.1	iron
BJP_8916	high-affinity zinc uptake system membrane protein znuB	*Escherichia coli*	347	347	98%	3,00E-117	99%	WP_001413405.1	zinc
BJP_4781	heavy metal sensor histidine kinase	*Pseudomonas* sp.	485	485	98%	6,00E-166	67%	WP_020797496.1	heavy metal
BJP_7694	heavy metal sensor signal transduction histidine kinase	*Comamonas testosteroni*	206	206	96%	2,00E-60	59%	YP_003278516.1	heavy metal
BJP_8034	ferric uptake regulator family protein	*Verminephrobacter eiseniae*	204	204	73%	3,00E-64	78%	YP_998128.1	iron
AP_31	metal transport system ATP-binding protein	*Sinorhizobium fredii*	179	179	71%	2,00E-52	71%	YP_006397726.1	zinc, manganese
BJP_7226	ArsC	*Sinorhizobium sp.*	293	293	99%	3,00E-99	100%	YP_007974289.1	arsenic
AP_188	copper oxidase	*Ochrobactrum rhizosphaerae*	851	851	9,90E-01	0.0	100%	WP_024899901.1	copper

Table S7. Genes potentially involved in degradation of specific pesticides as identified by BLASTp analyses.

Substrate	Gene(s)	Enzyme(s)	Accesion number/ Reference
Methyl viologen	*smv*A	Major facilitator superfamily transporter	AAL20492/ WP_003234942
Linuron	*hil*A	hydrolase	AGF25452
	*lib*A	hydrolase	AEO20132
	*puh*A	hydrolase	EU851877
	*puh*B	hydrolase	EU851876
2,4-D	*tfd*A	dioxygenase, alpha-ketoglutarate-dependent	YP_006963028
	*tfd*B	2,4-dichlorophenol hydroxylase	YP_006963026
	*cad*RABKC	transcriptional regulator, 2,4-D oxygenase large subunit of oxygenase, 2,4-D oxygenase small subunit of oxygenase, 2,4-D transporter, 2,4-D oxygenase ferredoxin	AB062679
1,4-Dichlorobenzene	*tcb*A	chlorobenzene dioxygenase	WP_032488666
	*tcb*B	cis-chlorobenzene dihydrodiol dehydrogenase	ABR26229
	*tcb*C	catechol 1,2-dioxygenase	AAD13625
Dibenzofuran	*dfd*A cluster	dibenzofuran 4,4a-dioxygenase alpha and beta subunits and ferredoxin	AB373750
	*dfd*BC cluster	two hypothetical proteins, tetR-type regulator, 2,2',3-trihydroxybiphenyl 1,2-dioxygenase, 2-hydroxy-6-(2-hydroxyphenyl)-6-oxo-2,4 hexadienoic acid hydrolase	AB373749

	*bph*BCD cluster	dihydrodiol dehydrogenase, 2,3-dihydroxybiphenyl dioxygenase, 2-hydroxy-6-phenylhexa-2,4-dienoic acid hydrolase	AJ539227
Endosulfan	*esd*	FMNH2-dependent mono-oxygenase	AF537302
	*dsz*A	monooxygenase	AAU14820
	*dsz*B	Substrate binding domain of 2'-hydroxybiphenyl-2-sulfinate desulfinase	AAU14821
	*dsz*C	sulfur acquisition oxidoreductase	AAU14822
3-Fluorobenzoate	*ben*A	benzene 1,2-dioxygenase	WP_043251427
	*xyl*L	1,6-dihydroxycyclohexa-2,4-diene-1-carboxylatedehydrogenase	CDF83450
4-Fluorobenzoate	*ben*A	benzene 1,2-dioxygenase	WP_043251427
	*xyl*Z	toluate 1,2-dioxygenase	CDF83449
	*cat*A3	catechol 1,2-dioxygenase	CDF83454
	*cat*B	muconate cycloisomerase 1	CDF83452
	*clc*D	dienelactone hydrolase	CDF83124
Parathion	*opd*	organophosphate-degrading protein	YP_009089179
Atrazine	*tri*A, *atz*A, *atz*B, *atz*C, *atz*D, *trz*B, *thc*, *apobec*	complete pathway	Database constructed by Fang *et al.* (Fang, *et al.*, 2014)
Hexachlorocyclohexane (HCH)	*dha*, *cbd*, *rdt*, *mog*, *rdg*, *dhc*, *dcn*, *dog*, *ccd*, *dhg*	complete pathway	Database constructed by Fang *et al.*, (Fang, *et al.*, 2014)
Dichlorodiphenyltrichloroethane (DDT)	*dhc*, *ods*, *rrat*, *cpo*, *rdh*, *doa*, *sds*, *ort*, *dhg*, *hdl*, *hdg*	complete pathway	Database constructed by Fang *et al.*, (Fang, *et al.*, 2014)

Table S8. List of putative genes involved in degradation of xenobiotic* and aromatic hydrocarbon compounds identified in the plasmid sequence dataset.

Substrate	GenDB ID	Description (best BLASTp hit at NCBI)	Organism	Max score	Total score	Query cover	E value	Max ident	Accession number	Observations
**2,4-dichlorophenoxyacetic acid**	BJP_2678	2,4-dichlorophenoxyacetate dioxygenase	*Burkholderia dolosa*	234	234	99%	1,00E-71	44%	WP_006764062.1	*tfd*A gene
	BJP_155	taurine catabolism dioxygenase	*Acidovorax* sp.	384	384	97%	2E-130	62%	GAD20643.1	*tfd*A gene
	BJP_2488	phenol 2-monooxygenase	*Acinetobacter* sp.	539	539	97%	0.0	61%	KEC84934.1	*tfd*B gene
	BJP_10655	Alpha-ketoglutarate-dependent taurine dioxygenase	*Achromobacter xylosoxidans*	326	326	88%	2E-108	68%	YP_008028310.1	*tfd*A gene
**Atrazine**	BJP_9495	chlorohydrolase	*Paenibacillus* sp.	150	150	98%	2E-41	96%	ETT37468.1	*atz*A gene
**3-Fluorobenzoate**	BJP_2294	oxidoreductase	*Variovorax* sp.	764	764	98%	0.0	85%	WP_028248695.1	benzoate 1,2-dioxygenase, subunit alpha
	BJP_2293	aromatic 1,2-dioxygenase subunit beta	*Variovorax paradoxus*	263	263	99%	9E-87	83%	WP_019654664.1	
	BJP_10023	6-chlorohydroxyquinol-1,2-dioxygenase	*Acidovorax* sp.	484	484	98%	4E-170	83%	WP_007855039.1	
**Dibenzofuran**	BJP_4909	2,3-dihydroxy-2,3-dihydrophenylpropionate dehydrogenase	*Escherichia coli*	550	550	100%	0.0	99%	P0CI32.1	2,3-dihydroxy-2,3-dihydrophenylpropionate dehydrogenase
	BJP_6277	glyoxalase	*Methylocella silvestris*	407	407	98%	4,00E-137	64%	WP_012590495.1	2,3-dihydroxybiphenyl 1,2-dioxygenase
	AP_378	2-hydroxy-6-oxo-6-phenylhexa-2,4-dienoate hydrolase	*Pseudomonas* sp.	508	508	100%	3E-179	86%	KFJ90076.1	2-hydroxy-6-oxo-6-phenylhexa-2,4-dienoate hydrolase
**Parathion**	BJP_3668	phosphotriesterase homology protein	*Escherichia coli*	608	608	100%	0.0	99%	WP_000007005.1	

**methyl viologen**	BJP_8262	hypothetical protein	alpha proteobacterium	489	489	96%	4,00E-167	99%	WP_017502939.1	MFS transporter, methyl viologen resistance protein SmvA
	BJP_3584	membrane protein	*Enterobacter* sp.	771	771	99%	0.0	100%	YP_008098281.1	MFS transporter, methyl viologen resistance protein SmvA
	BJP_4968	major facilitator superfamily protein	*Comamonas testosteroni*	204	204	95%	3,00E-59	65%	YP_003277211.1	methyl viologen resistance protein
**Xylene/ toluene/ bencene**	BJP_5604	4-oxalocrotonate decarboxylase	*Pseudomonas putida*	538	538	99%	0.0	99%	NP_542860.1	4-Oxalocrotonate Tautomerase
	BJP_5605	ABC transporter, permease protein	*Pseudomonas putida*	125	125	98%	8,00E-36	100%	NP_542859.1	
	BJP_5606	regulatory protein	*Pseudomonas putida*	294	294	99%	2,00E-97	100%	NP_542858.1	
	BJP_8726	4-oxalocrotonate decarboxylase	*Burkholderia* sp.	358	358	99%	2,00E-121	79%	ACO92645.1	
	BJP_5748	major facilitator transporter	*Burkholderia terrae*	362	362	87%	2,00E-119	76%	WP_007588851.1	benzoate transport protein BenK
	BJP_5073	Rieske (2Fe-2S) region	*Dechloromonas aromatica*	105	105	88%	1,00E-26	54%	YP_287016.1	Toluene-4-monooxygenase effector protein complex
	BJP_9384	ferredoxin--NAD(+) reductase FAD/NAD(P)-binding protein	*Cupriavidus metallidurans*	223	223	95%	2,00E-66	41%	YP_583463.1	Toluene-4-monoxygenase electron transfer component
	BJP_10390	hypothetical protein	*Ramlibacter tataouinensis*	280	280	82%	3,00E-92	72%	YP_004617916.1	toluene tolerance family protein
	bjp_1643	benzyl alcohol dehydrogenase	*Pseudomonas* sp.	325	435	99%	7E-108	97%	WP_028624487.1	*tdh* gene
	bjp_1644	benzyl alcohol dehydrogenase	*Pseudomonas putida*	281	281	99%	7E-94	98%	WP_004575908.1	*tdh* gene
	bjp_1645	benzaldehyde dehydrogenase	*Pseudomonas putida*	936	936	99%	0.0	98%	NP_542888.1	*xyl*C gene
	bjp_1646	xylene monooxygenase	*Pseudomonas* sp.	727	727	99%	0.0	94%	WP_028624485.1	*xyl*M gene

bjp_1647	xylene monooxygenase electron transfer component	*Pseudomonas putida*	707	707	99%	0.0	99%	YP_003617172.1	*xil*A gene
bjp_1648	alcohol dehydrogenase	*Pseudomonas* sp.	686	686	99%	0.0	99%	WP_028624483.1	

***Reference:** Fang, H., *et al*., Metagenomic analysis reveals potential biodegradation pathways of persistent pesticides in freshwater and marine sediments. *Sci Total Environ*. **470-471**:983-92 (2014)

Table S9. Transposon and IS identified in the plasmid dataset.

IS name	Family	Origin
ISUnCu4	IS30	Plasmid QKH54
TnAs2	Tn3	*Aeromonas salmonicida*
ISStma17	IS3	*Stenotrophomonas maltophilia*
ISSso4	IS21	*Shigella sonnei*
ISSso4	IS21	*Shigella sonnei*
ISRssp4	IS30	*Roseovarius sp.*
IS2020	IS6	*Brucella melitensis*
ISEc1	IS3	*Escherichia fergusonii*
ISSfl3	IS66	*Shigella flexneri*
TnShfr1	Tn3	*Shewanella frigidimarina*
IS869	IS5	*Agrobacterium tumefaciens*
IS869	IS5	*Agrobacterium tumefaciens*
ISPa16	IS4	*Pseudomonas aeruginosa*
ISCte2	IS3	*Comamonas testosteroni*
ISKpn25	ISL3	*Klebsiella pneumoniae*
ISMex9	IS110	*Methylobacterium extorquens*
ISSba14	Tn3	*Shewanella baltica*
ISKpn26	IS5	*Klebsiella pneumoniae*
ISHne5	IS110	*Hyphomonas neptunium*
ISRtr2	IS3	*Corynebacterium jeikeium*
ISRtr2	IS3	*Corynebacterium jeikeium*
ISRssp4	IS30	*Roseovarius sp.*
ISVsa3	IS91	*Vibrio salmonicida*
ISYps3	Tn3	*Yersinia pseudotuberculosis*
ISYps3	Tn3	*Yersinia pseudotuberculosis*
IS609	IS200/IS605	*Escherichia coli*
IS609	IS200/IS605	*Escherichia coli*
ISPa38	Tn3	*Pseudomonas aeruginosa*
ISMex40	IS5	*Methylobacterium extorquens*
ISRsp9	IS6	*Rhizhobium sp.*
ISMex22	Tn3	*Methylobacterium extorquens*
ISXc4	Tn3	*Xanthomonas campestris*
ISAba30	IS200/IS605	*Acinetobacter baumannii*
ISPre1	IS5	*Pseudomonas resinovorans*
ISXca1	IS3	*Xanthomonas campestris*
ISPa42	Tn3	*Pseudomonas aeruginosa*
ISPa42	Tn3	*Pseudomonas aeruginosa*
ISXc4	Tn3	*Xanthomonas campestris*

ISPpu12	ISL3	*Pseudomonas putida*
ISPpu12	ISL3	*Pseudomonas putida*
ISPpu12	ISL3	*Pseudomonas putida*
ISPst9	ISL3	*Pseudomonas stutzeri*
ISRel8	IS66	*Rhizobium etli*
ISRel8	IS66	*Rhizobium etli*
ISPsy24	IS3	*Pseudomonas syringae*
ISXax1	IS256	*Xanthomonas axonopodis*
TnAs1	Tn3	*Aeromonas salmonicida*
ISSwi1	Tn3	*Salmonella wien*
TnAs2	Tn3	*Aeromonas salmonicida*
ISThsp9	Tn3	*Salmonella wien*
ISPsy30	Tn3	*Pseudomonas syringae*
ISXc4	Tn3	*Xanthomonas campestris*
ISAzo2	IS91	*Weeksella zoohelcum*
ISMex22	Tn3	*Methylobacterium extorquens*
ISMtsp16	IS481	*Methylobacterium sp.*
IS15DIV	IS6	*Salmonella typhimurium*
ISAav1	IS21	*Acidovorax avenae*
ISAav1	IS21	*Acidovorax avenae*
ISRso21	ISL3	*Ralstonia solanacearum*
ISMmg1	IS110	*Magnetospirillum magnetotacticum*
ISAtu3	IS3	*Agrobacterium tumefaciens*
ISSpo6	IS630	*Silicibacter pomeroyi*
ISMmg1	IS110	*Magnetospirillum magnetotacticum*
ISVsa17	ISNCY	*Aliivibrio salmonicida*
IS1394	IS30	*Pseudomonas alcaligenes*
ISRel10	ISNCY	*Rhizobium etli*
ISVsa17	ISNCY	*Aliivibrio salmonicida*
IS4	IS4	
ISAzs29	Tn3	*Azospirillum sp.*
ISGme8	IS110	*Geobacter metallireducens*
ISPps1	IS91	*Pseudomonas huttiensis*
IS693	IS66	*Sinorhizobium medicae*
ISAzs17	Tn3	*Azospirillum sp.*
IS1071	Tn3	*Alcaligenes sp*
ISRsp12	Tn3	*Rhizhobium sp.*
ISCARN95	IS21	Metagenomic data
ISCARN24	IS21	Metagenomic data
ISPlu15	ISNCY	*Photorhabdus luminescens*
ISShes11	Tn3	*Shewanella sp.*
ISRsp18	IS5	*Rhizobium sp.*

ISEc11	IS110	*Escherichia coli*
ISWpi13	IS110	*Wolbachia pipientis*
ISAzo28	IS110	*Azoarcus sp.*
IS1492	IS110	*Pseudomonas putida*
ISKpn1	IS3	*Klebsiella pneumoniae*
ISKpn1	IS3	*Klebsiella pneumoniae*
ISRle11	IS1182	*Rhizobium leguminosarum*
ISRme12	IS3	*Ralstonia metallidurans*
IS150	IS3	*Escherichia coli*
ISAzvi6	IS4	*Azotobacter vinelandii*
ISEc26	ISAs1	*Escherichia coli*
ISMdi9	IS110	*Methylobacterium dichloromethanicum*
ISSpr1	IS3	*Serratia proteamaculans*
ISPsy9	IS3	*Pseudomonas syringae*
ISJs1	IS481	*Janibacter sp.*
IS1271	IS4	*Enterobacter agglomerans*
ISPfl1	IS110	*Pseudomonas fluorescens*
IS421	IS4	*Escherichia coli*
ISSba14	Tn3	*Shewanella baltica*
ISPpu15	IS66	*Pseudomonas putid*
ISCte5	IS5	*Comamonas testosteroni*
ISStma6	IS110	*Stenotrophomonas maltophilia*
IS401	IS3	*Burkholderia cepacia*
ISAav4	IS3	*Acidovorax avenae*
ISPsy11	IS3	*Pseudomonas syringae*
ISPsy11	IS3	*Pseudomonas syringae*
ISHne2	IS481	*Hyphomonas neptunium*
ISPsy28	IS3	*Pseudomonas syringae*
ISThsp9	Tn3	*Salmonella wien*
ISPpu10	IS110	*Pseudomonas putida*
IS5D	IS5	*Escherichia coli*
ISAch1	IS5	*Achromobacter sp.*
ISPpu10	IS110	*Pseudomonas putida*
ISMet1	ISKra4	*Methylocystis sp.*
TnAs1	Tn3	*Aeromonas salmonicida*
ISPpa9	IS6	*Paracoccus pantotrophus*
ISMex24	ISL3	*Methylobacterium extorquens*
ISSod9	Tn3	*Shewanella oneidensis*
IS903	IS5	*Escherichia coli*
ISPpu4	IS3	*Pseudomonas putida*
